# Structural and functional analysis **of** *Escherichia coli* membrane disruption **by** Ib-M peptides

**DOI:** 10.1371/journal.pone.0334029

**Published:** 2025-10-08

**Authors:** Ana Elvira Farfán-García, Indira Paola Hernández-Peñaranda, Oscar G. Gómez-Duarte, Edgar Javier Rincón-Barón, Gerardo Andrés Torres-Rodríguez, Johanna Marcela Flórez-Castillo

**Affiliations:** 1 Facultad de Ciencias Médicas y de la Salud, Instituto de Investigaciones Masira, Universidad de Santander, Bucaramanga, Santander, Colombia; 2 Corporación de Ciencias Básicas Biomédicas, Universidad de Antioquia, Medellín, Antioquia, Colombia; 3 Facultad de Ciencias Exactas, Naturales y Agropecuarias, Universidad de Santander, Bucaramanga, Santander, Colombia; 4 Division of Pediatric Infectious Diseases and Immunology, Department of Pediatrics, Levine Children’s Hospital at Advantage Health and Wake Forest University School of Medicine, Charlotte, North Carolina, United States of America; 5 Unidad de Microscopía Electrónica, Universidad del Cauca, Popayán, Cauca, Colombia; University of Buea, CAMEROON

## Abstract

Antimicrobial resistance represents a critical global public health challenge, leading to increased mortality and morbidity due to the ineffectiveness of current antibiotics against bacterial infections. Antimicrobial peptides (AMPs) offer a promising alternative for treating bacterial infections because of their broad-spectrum activity, biocompatibility, and rapid bactericidal action. Recent studies have demonstrated that Ib-M peptides exhibit bactericidal activity against pathogenic *Escherichia coli* clinical isolates. The objective of this study was to evaluate the mechanisms by which Ib-M peptides destabilize and disrupts *E. coli* membranes. We showed by dilutions assays that Ib-M peptides had a minimum inhibitory concentration (MIC) of 12.5 µM against *E. coli.* Electron microscopy studies confirmed that Ib-M peptides were directly implicated in *E. coli* membrane disruption, altered bacterial shape and subsequent disintegration. To understand bacterial membrane interaction with Ib-M peptides at the molecular level, we evaluated structure-function relationships using circular dichroism spectroscopy and *in silico* simulations. These studies demonstrated the strong amphipathic, hydrophobic and cationic properties of Ib-M peptides. At sublethal concentrations, these peptides interacted with bacterial lipopolysaccharides (LPS), leading to outer and inner membrane permeation and cytoplasmic membrane depolarization. This effect was transient at sublethal Ib-M peptides concentrations, as evidenced by the recovery of bacterial growth in *lag* phase kinetics. At higher concentrations, there was high depolarization of cytoplasmic membrane, disruption of outer membrane and inner membranes and irreversible bacterial lysis. When mammalian cells were exposed Ib-M1 peptide cytotoxic effect was only reached when MIC was increased 10-fold. In conclusion, Ib-M peptides inhibited *E. coli* growth by disrupting bacterial membranes via interactions with LPS and increased membrane permeation yet, they have low cytotoxicity on mammalian cells. This study highlights the mechanisms of action on Ib-M peptides as antimicrobials and paves the way for further research on the clinical use of these peptides as antimicrobial agents against multidrug resistant bacterial infections.

## Introduction

The spread of antimicrobial resistance (AMR) in Gram-negative bacteria, especially *Escherichia coli*, has exacerbated the global burden of infectious diseases due to increased hospitalizations, mortality, sequelae, and the resulting rise in healthcare expenses [1–5].

Multidrug-resistant *E. coli* have a significant impact on global public health due to the rapid dissemination of bacterial genes encoding extended-spectrum β-lactamases (ESBLs), carbapenemases, efflux pumps, and porin mutants [6,7].

In 2024, the World Health Organization (WHO) placed *E. coli* resistant to carbapenem and third-generation cephalosporins at the top of the warning list of pathogens [8]. To address the impact of multidrug resistant (MDR) bacterial infections, research on alternative antimicrobial products, including antimicrobial peptides (AMP), is of critical importance.

AMPs have emerged as promising candidates for the treatment of bacterial infections because of their rapid biological action, broad-spectrum activity, synergy with antibiotics, valuable immunomodulatory properties, and biocompatibility [9–14].

The AMPs are a diverse group of small bioactive proteins comprised of 12–45 amino acids with cationic properties that work as first line of defense against pathogens in living organisms [15–17]. Furthermore, some AMPs can be anionic (AAMPs) [18,19]. The action of AMPs on bacteria is determined by their size, charge, hydrophobicity, secondary structures, and amphiphilic characteristics [20–24].

Ib-AMP4 is a native peptide that plays an important role in the defense of germinated seeds of *Impatiens balsamina* plants against fungal and Gram-positive bacteria [25]. Flórez et al. designed six analogous peptides (Ib-M1, Ib-M2, Ib-M3, Ib-M4, Ib-M5, and Ib-M6) [26] based on the amino acid sequence of the Ib-AMP4 peptide [25]. Substitution of four cysteines (C) of the native Ib-AMP4 peptide with methionine (M) residues in Ib-M peptides, along with the addition of arginine (R) and tryptophan (W) to the sequence, increased the net charge and hydrophobicity; which promoted a better bactericidal effect against *E. coli* K12 and low cytotoxicity in red blood cells [26].

Recent evidence has demonstrated promising antibacterial activity of Ib-M peptides against clinical and reference strains of enterohemorrhagic *E. coli*. For bactericidal action, these peptides required concentrations between 1.6 to 12.5 µM, and induced cytotoxicity in Vero cells within a range of 197.5 to > 400 µM [27]. Additionally, in bactericidal assays, the Ib-M1 peptide at the minimum inhibitory concentration (MIC) reduced the bacterial population by 95% after 4 hours of exposure. In a separate study, the Ib-M6 peptide exhibited activity against fourteen pathogenic and non-pathogenic clinical isolates of *E. coli*, with MICs ranging from 0.7 to 25 µM [28]. Regarding Gram-positive microorganisms, we demonstrated that Ib-M peptides displayed antimicrobial activity against *Staphylococcus aureus* ATCC 25923 and *Candida albicans* ATCC 10231 between 25 µM to 100 µM (unpublished results).

Although the biological activity of Ib-M1, Ib-M2, and Ib-M6 peptides and their low cytotoxicity in mammalian cells are well-recognized, the mechanism by which these peptides interact with the *E. coli* membrane to exert bactericidal activity remains unknown. With the goal of reaching a better understanding of Ib-M peptides mechanism of action at the molecular level, we conducted studies to confirm bactericidal effect, to observe membrane damage, and to evaluate secondary structures and physicochemical properties to identify the structure-function relationships of Ib-M peptides and bacterial targets. We excluded Ib-M3, Ib-M4, and Ib-M5 peptides from these studies since they exhibited lower biological activity against clinical *E. coli* isolates [27,28]. This lower activity is likely due to finding from *in silico* analysis demonstrating lower normalized hydrophobic moments (0.19 to 0.38), smaller hydrophobic face of Ib-M3 (only methionine and tryptophan), and absent hydrophobic face of Ib-M4 (unpublished results). Based on the above, the objective of this study was to evaluate the mechanisms by which Ib-M peptides destabilize and disrupt *E. coli* membranes at the structure and function and molecular levels.

## Materials and methods

### Bacterial strains, cell lines, and culture conditions

*E. coli* (Migula) Castellani and Chalmers (ATCC 25922) was obtained from the American Type Culture Collection (Manassas, VA, USA). *E. coli* ATCC 25922 is a standard strain widely used in microbiological and antibiotic sensitivity studies, it was selected as a model to evaluate the mechanisms of action of peptides Ib-M1, Ib-M2 and Ib-M6. *E. coli* ML35 (ATCC 43827) was utilized for inner membrane (IM) permeability assays. Bacteria were cultivated in Luria-Bertani (LB) medium (Sharlau, Sharlab, S.L., Barcelona, Spain) (1% peptone, 0.3% beef extract, and 0.5% NaCl) at 37° C for 18 h unless otherwise specified.

Tissue culture cells NCM460 (normal human colon mucosal epithelial cells) ATCC CVCL-0460, SW480 ATCC CCL-228, and Caco2 (colorectal adenocarcinoma cells) ATCC® HTB-37™ were obtained from ATCC (Manassas, VA, USA) and cultured under standard conditions using Dulbecco’s Modified Eagle Medium (DMEM) high glucose (Sigma-Aldrich Co., St. Louis, MO, USA) in a humidified incubator with an atmosphere of 95% air and 5% CO_2_ at 37° C. DMEM was supplemented with 10% heat-inactivated fetal bovine serum (FBS, Invitrogen Ltd, Paisley, UK), 2 mM L-glutamine, 1 mM sodium pyruvate, sodium bicarbonate, 1% non-essential amino acids, 100 units/mL penicillin, and 100 μg/mL streptomycin (Sigma-Aldrich Co., St. Louis, MO, USA).

### Peptide synthesis

The peptides Ib-M1, Ib-M2, and Ib-M6 were obtained from GenScript® (Piscataway, NJ, USA) using solid-phase 9-fluorenyl methoxycarbonyl (Fmoc) chemistry with C-terminal amidation and the free N-terminus. Peptide purity (≥ 95%) and molecular weight were verified by high-performance liquid chromatography (HPLC) and mass spectrometry (MS) analyses, respectively (S1 Table, S1 and S2 Fig). The Ib-M peptide identities and values obtained from theoretical and observed weights by mass spectrometry were consistent (S1 Table, S1 Fig). Similarly, the HPLC retention times showed that the Ib-M6 peptide exhibited the highest hydrophobicity, based on the longest retention time observed; followed by Ib-M1 and Ib-M2 (S1 Table, S2 Fig). The sequences, net charges, and numbers of amino acids are summarized in S1 Table. Solubility assessments were conducted for each peptide. The powdered peptides were stored at −20° C until use and, then were dissolved in Tris-HCl buffer (10 mM, pH 7.4) at a concentration of 2 mM. Aliquots were stored at −80° C.

### Antibacterial assays

The MIC of the Ib-M1, Ib-M2, and Ib-M6 peptides against *E. coli* ATCC 25922 were evaluated using the Müller-Hinton broth (MHB) (Merck, Darmstadt, Germany) microdilution method, in accordance with the Clinical and Laboratory Standards Institute (CLSI) (M07-A9- 2012) [29].

*E. coli* was cultured in LB broth for 16–18 hours at 37° C. Bacterial suspensions were adjusted to an 0.5 OD_600nm_ (1 × 10^8^ colony-forming unit (CFU/mL). The bacterial suspension was then diluted 100-fold in MHB to obtain 1 × 10^6^ CFU/mL, and it was combined with serial dilutions of Ib-M1, Ib-M2, and Ib-M6 peptides to obtain 5 × 10^5^ CFU/mL and peptide concentrations ranging from 100 to 3.1 μM. The plate was incubated at 37° C with constant agitation at 120 rpm for 24 h, with endpoint measurements taken using a microplate reader (Varioskan Lux, Thermo Scientific, MA, USA) and unaided naked eyes. The MIC was reported as the lowest peptide concentration at which no visible bacterial growth was observed after incubation. In addition, inhibition of the growth kinetics of *E. coli* ATCC 25922 was evaluated upon treatment with Ib-M peptides with absorbance measurements taken every 2 h for 24 h [27]. Streptomycin was used as a positive control at concentrations from 50 µM to 1.5 µM, while MHB without bacteria and a bacterial growth control without compounds were utilized as negative controls. In addition, the MIC of Polymyxin B (PMB) (Santa Cruz Biotechnology, Inc. Dallas, TX, USA) was established, which was used as a control in morphological and biophysical experiments.

Following the MIC determination of the Ib-M1, Ib-M2, and Ib-M6 peptides, the minimum bactericidal concentration (MBC) was established. For this purpose, 10 μL aliquots from wells exhibiting no bacterial growth were inoculated onto 24-well plates containing LB agar (Sharlau, Sharlab, S.L., Barcelona, Spain) and were incubated at 37° C for 24 h. The MBC was the lowest concentration of peptides that eliminated ≥ 99.9% of the bacteria, in accordance with the method established by CLSI [29].

### Sensitivity of Ib-M peptides to salts

The sensitivity of the Ib-M peptides to salts, and their effect on antibacterial activity against *E. coli* ATCC 25922 were evaluated using MIC assays [29,30]. For these assays, in 96-well U-bottom plates, final concentrations of salts (150 mM NaCl, 4.5 mM KCl, 1 mM MgCl_2,_ and 2 mM CaCl_2_) (Merck, Darmstadt, Germany); Ib-M1, Ib-M2, and Ib-M6 peptides (50 µM to 3.1 µM); and bacteria (5 × 10^5^ CFU/mL of *E. coli* ATCC 25922) were combined in MHB. The procedure previously described for MIC determination was employed, with absorbance measurements taken every 2 h for 24 h. The MBC was determined from wells where no growth was observed, as previously described. Wells containing *E. coli* ATCC 25922 and Ib-M1, Ib-M2, or Ib-M6 peptides at concentrations of 4 × MIC, 2 × MIC, 1 × MIC, and 0.5 × MIC in MHB without salts, and wells containing MHB without bacteria served as controls.

### Scanning electron microscopy (SEM)

SEM was used to obtain 3D images of Ib-M-exposed bacteria to identify morphology changes. *E. coli* inoculum was adjusted to 2 × 10^8^ CFU/mL. Ib-M peptides at final concentrations (200 µM Ib-M1, 100 µM Ib-M2, and 200 µM Ib-M6) were combined in a 1:1 ratio with the *E. coli* inoculum. Each treatment was incubated at 37° C with constant agitation at 120 rpm for 30, 60, or 120 min. Following incubation, the samples were centrifuged, washed twice with Phosphate Buffer Saline (PBS) (0.1 M, pH 7.2), and fixed overnight at 4° C with 4% glutaraldehyde. After two washes and centrifugation at 4500 rpm for 5 min, the samples were fixed with 1% osmium tetroxide (OsO_4_); then washed with water and gradually dehydrated using ethanol (10%−100%). Hexamethyldisilazane (HDMS) with ethanol 1:1 (v/v), followed by pure HDMS, were utilized for sample desiccation as a substitute for critical point drying, incorporating minor modifications [31]. Subsequently, the samples were mounted on stubs, air-dried, and coated with gold. Controls included untreated and PMB-treated bacteria (1.5 µM). SEM was performed on the samples using a Quanta FEG 650 at 30 kV in a high vacuum.

### Transmission electron microscopy (TEM)

TEM was used to obtain 2D images of bacteria exposed to Ib-M to evaluate morphological changes at the membrane and cytosol levels. Specimens for TEM were prepared in accordance with a previously described protocol, incorporating minor modifications [31,32]. The preparation of the bacterial inoculum and its exposure to the Ib-M peptides and their respective controls were conducted as previously described for SEM. Following 60 minutes of bacterial-peptides incubation treatment, the specimens were washed three times in 0.1 M PBS (pH 7.2). Subsequently, the bacterial pellets were fixed in 2.5% glutaraldehyde in 0.1 M PBS (pH 7.2) for 72 h and washed in PBS as described. A secondary fixation was then performed in 2% OsO_4_, followed by similar washing. The cell suspension obtained from each specimen was embedded in a 3% agar base according to the Dykstra and Reuss procedure and subsequently dehydrated with a series of ethanol concentrations, culminating in absolute ethanol [32]. The alcohol was then removed, and the specimens were embedded in London Resin Co. (LR) White Resin and polymerized under ultraviolet (UV) light for 12 h. Each sample was sliced into 70–80 nm ultrathin sections, then counterstained with aqueous uranyl acetate and lead citrate. Finally, each specimen was examined using a JEOL JEM 1200 EX Transmission Electron Microscope. Controls comprised untreated and PMB-treated bacteria (1.5 µM).

### Cytotoxicity assays

The cytotoxicity of the Ib-M1, Ib-M2, and Ib-M6 peptides was evaluated using a colorimetric MTT assay in the cell lines NCM-460, Caco2, and SW480. The assay measures the reduction of 3-(4,5-dimethylthiazol-2-yl)-2,5-diphenyltetrazolium bromide (MTT) (Sigma-Aldrich Co., St. Louis, MO, USA) to water-insoluble purple formazan crystals. Cells were treated with peptide concentrations ranging from 1.5 to 50 μM, following a previously described protocol [13]. Each cell line was seeded in flat-bottom 96-well plates at a density of 2 × 10^4^ cells/mL, with the plates then incubated at 37° C with 5% CO_2_ until 90% confluency was achieved. Subsequently, the cells were treated with 100 μL of Ib-M peptides at two-fold dilutions from 200 to 1.5 μM for 24 h under the same conditions. Following incubation, 20 μL of 5 mg/mL MTT in 0.01 M PBS (Sigma-Aldrich Co., St. Louis, MO, USA) (pH 7.2) was added to each well, and the plates were again incubated for 4 h. Thereafter, the medium containing MTT was removed, and the formazan crystals that accumulated intracellularly and on the cell surface were dissolved in 100 μL dimethyl sulfoxide (DMSO) (Merck, Darmstadt, Germany). Finally, MTT reduction was detected as changes in absorbance measured at 570 nm with spectrophotometry (Varioskan Lux, Thermo Scientific, MA, USA). The percentage of cellular toxicity was calculated using the following Equation 1:


$$\%\;Cytotoxicity=\left[\text{1}-\left(\frac{Absorbance\;of\;cells\;with\;peptide}{Absorbance\;of\;cells\;without\;peptide}\right)\right]x\;\text{1}\text{0}\text{0}$$ (1)

Subsequently, the inhibitory concentration (IC_50_), corresponding to a survival rate of 50% of cells exposed to the peptide, was calculated. The higher the peptide IC_50_ the lower the cytotoxicity. The selectivity index (SI) value was obtained from the ratio between the IC_50_ values of the non-tumor cell and that of the MIC against *E. coli* ATCC 25922 [33,34]. The higher the selectivity index, the higher the bactericidal effect and the lower the cytotoxic effect on human cells. IC_50_ values of each peptide in the non-tumor cell line and the IC_50_ value in the tumor line were also calculated [35].

### Physicochemical properties analysis and *in silico* molecular modeling

The Heliquest online server (https://heliquest.ipmc.cnrs.fr/cgi-bin/ComputParams.py) was utilized to generate helical wheel projections [36]. Physicochemical properties were calculated using the Database of Antimicrobial Activity and Structure of Peptides (DAASP) server (https://dbaasp.org/tools?page=property-calculation) [37,38], and the Antimicrobial Peptide Calculator and Predictor (APD3) (https://aps.unmc.edu/prediction) [39]. To illustrate the distribution of amino acid residues, the chemical structure was rendered using Pepdraw (https://pepdraw.com/). The secondary structures of Ib-M1, Ib-M2, and Ib-M6 peptides were modeled employing PepFold 3.5 software (https://mobyle.rpbs.univ-paris-diderot.fr/cgi-bin/portal.py#forms::PEP-FOLD3) [40]. The optimal models were selected based on quality criteria, which encompassed the Mol-Probity Score, Ramachandran favored percentage (%), and clash score, following the Swiss Model (https://swissmodel.expasy.org/assess) [41] (S2 Table). The molecular structure and electrostatic potential surface of these models were visualized utilizing Discovery Studio Server 2021 by Accelrys Inc. (https://discover.3ds.com/discovery-studio-visualizer-download) and UCSF ChimeraX (https://www.cgl.ucsf.edu/chimerax/) [42,43].

### Deconvolution of Circular Dichroism (CD) data

The secondary structure content of the Ib-M peptides was determined using the Beta Structure Selection (BeStSel) software (http://bestsel.elte.hu/) [44]. The parameters obtained from the deconvolution process were compared with the CD spectra previously published by our group [27]. The data acquired via CD from 190 to 250 nm were subjected to a deconvolution process to obtain the molar extinction [45]. Subsequently, the molar extinction data and wavelength were input online into BeStSel (https://bestsel.elte.hu/index.php) to calculate the secondary structure content of the peptides, including β-sheet, α-helix, disordered, and turns [44–46].

### Simulations of Ib-M peptide positioning on the bacterial membrane

Subsequently, 3D simulations of Ib-M peptides obtained using PEP-FOLD 3.5 were employed to calculate peptide positioning in membrane (PPM), using the online PPM 3.0 tool (https://opm.phar.umich.edu/ppm_server3_cgopm) [47,48]. The Protein Data Bank (PDB) files of the peptides were used as input files to calculate their angle of interaction, transfer free energy and the penetration depth penetration in the outer membrane (OM) Gram-negative.

### LPS displacement cadaverine assay

The binding capacity of Ib-M peptides to the LPS of *E. coli* O111:B4 was evaluated using the BODIPY-TR cadaverine (BC) displacement assay with modifications [49]. In 50 mM Tris buffer (pH 7.4), 5 µg/mL BC (Thermo-Fisher Scientific, Whaltam, MA, USA) and 50 µg/mL *E. coli* LPS O111:B4 (Sigma-Aldrich Co., St. Louis, MO, USA) were mixed. The product (BC + LPS) was incubated at room temperature. Subsequently, 50 µL of the mixture and 50 µL of Ib-M1, Ib-M2, and Ib-M6 peptides in two-fold dilutions from 100 µM were added up to 0.79 µM (final) in black 96-well plates. PMB diluted from 50 to 0.79 µM was utilized as a positive control, and the BC/LPS mixture as the negative control. Fluorescence units were measured using a spectrophotometer (Varioskan Lux, Thermo Scientific, MA, USA) every minute for one hour at 580 nm excitation and 620 nm emission. The arbitrary fluorescence units (a. u.) thus obtained were utilized to determine the percentage of displacement according to Equation 2:


$$Displacement\;percentage=\frac{\left(F_{obs}-F_{\text{0}}\right)}{\left(F_{\text{1}\text{0}\text{0}}-F_{\text{0}}\right)}x\;\text{1}\text{0}\text{0}$$ (2)

where *F*_*obs*_ is the BC fluorescence intensity after treatment with a given peptide concentration; *F*_*0*_ is the initial fluorescence of BC treated with LPS in the absence of peptides; and *F*_*100*_ is the fluorescence of BC with LPS after addition of PMB at 10 µg/mL.

### Time-dependent permeabilization of the outer membrane (NPN Uptake Assay)

The N-phenyl-1-naphthylamine (NPN) (Merck, Darmstadt, Germany) uptake assay [50,51] with modifications for this study, was utilized to evaluate the effect of the Ib-M peptides on the outer membrane permeability of *E. coli* ATCC 25922. For this assay, *E. coli* ATCC 25922 colonies were harvested in buffer (5 mM HEPES, 5 mM glucose, pH 7.4) (Sigma-Aldrich Co., St. Louis, MO, USA), followed by two centrifugations. The resultant pellet was resuspended in the same buffer and adjusted to 2 × 10^8^ CFU/mL. In black 96-well flat-bottom plates, 50 µL of Ib-M peptides diluted in buffer (200–12.5 µM) were added, followed by 100 µL of the bacterial suspension (final concentration 1 × 10^8^ CFU/mL). Finally, 50 µL of NPN at final concentration of 60 µM was added to each well. The fluorescence of NPN as a function of time was monitored at two-minute intervals for one hour (excitation λ = 350 nm, emission λ = 420 nm) using a spectrophotometer (Varioskan Lux, Thermo Scientific, MA, USA). PMB served as a positive control, while HEPES + NPN + *E. coli* was employed as a negative control. NPN uptake percentage was calculated using the following Equation 3 [52]:


$$NPN\;uptake\;(\%)=\frac{\left(F_{obs}-F_{\text{0}}\right)}{\left(F_{\text{1}\text{0}\text{0}}-F_{\text{0}}\right)}x\;\text{1}\text{0}\text{0}$$ (3)

where *F*_*obs*_ is the observed fluorescence of peptides at a given concentration; *F*_*0*_ is the initial fluorescence of NPN without peptides; and *F*_*100*_ is the fluorescence of NPN upon addition of 12.5 µM PMB.

### *E. coli* inner membrane permeability

To determine whether the peptides permeabilized the inner membrane of *E. coli*, the exposure of the impermeable substrate O-nitrophenyl-β-D-galactopyranoside (ONPG) to bacterial cytoplasmic β-galactosidase was evaluated with brief modifications [53]. *E. coli* ML35p was utilized for this experiment due to its constitutive production of intracytosolic β-galactosidase and lack of expression of the permease transporter *lac* [53]. In this assay, *E. coli* ML35p cells were cultured in LB, followed by seeding on LB agar and incubation for an additional 24 h at 37° C. The colonies were then suspended in PBS at pH 7.4, harvested by centrifugation, and washed. The cell concentration was adjusted to 1 × 10^6^ CFU/mL. Subsequently, 100 µL of Ib-M peptides (ranging from 0.5 × MIC to 16 × MIC) and 50 µL of ONPG (final concentration 1.5 mM) were added to each well of a 96-well plate, followed by the addition of the bacterial inoculum. The permeability dynamics were monitored via ONPG substrate hydrolysis and O-nitrophenol production at 415 nm every 5 minutes over 8 h using spectrophotometry (Varioskan Lux, Thermo Scientific, MA, USA). Bacteria treated with PMB and streptomycin were used as positive and negative controls for permeabilization, respectively.

### Cytoplasmic transmembrane potential measurement

The effect of Ib-M peptides on the cytoplasmic membrane potential of *E. coli* was investigated using the lipophilic potentiometric dye 3,3’-dipropylthiadicarbocyanine iodide (DisC3 [5]) (Sigma-Aldrich Co., St. Louis, MO, USA) with modifications for this study [54,55]. For this assay, *E. coli* ATCC 25922, cultured for 3 h until an OD_600nm_ of 0.5, were harvested by centrifugation at 3000 rpm for 10 min, and the pellet was resuspended in energizing buffer (5 mM HEPES and 20 mM glucose, pH 7.4) and adjusted to OD_600nm_ 0.05 (2.5 × 10^7^ CFU/mL). Subsequently, EDTA (Bio-Rad, CA, USA) was added to a final concentration of 0.2 mM and incubated for 15 min at room temperature with agitation at 120 rpm. Thereafter, DisC_3_ [5] (Merck, Darmstadt, Germany) was introduced to a final concentration of 3.2 µM and incubated for 90 min. Then, KCl was introduced to achieve a final concentration of 200 mM, and the mixture was incubated for an additional 20 min. In black 96-well plates, 100 µL of bacterial suspension and control (0.5% Triton X-100 as a positive depolarizing control and *E. coli* without peptides as a negative control) were subjected to excitation at 620 nm and emission at 670 nm using a Varioskan Lux fluorescent spectrophotometer (Varioskan Lux, Thermo Scientific, MA, USA). After a 10-minute period for fluorescence stabilization, 100 µL of Ib-M peptides, ranging in concentration from 1.5 to 100 µM, were added and measured for 60 min at one-minute intervals.

### Statistical analysis

The data were analyzed using GraphPad Prism software v10.2.1 (GraphPad Software, Boston, Massachusetts USA, www.graphpad.com). Differences among groups were determined by one-way analysis of variance (ANOVA) followed by Tukey’s multiple comparisons test or paired Student’s t-test (two-tailed). The results were considered statistically significant at a value p < 0.05. All experiments were repeated at least three times, and the results are presented as mean ± SD. IC_50_ values were determined based on the dose-response curve.

## Results

### Ib-M peptides inhibit *E. coli* growth and are bactericidal

The three peptides analyzed (Ib-M1, Ib-M2, and Ib-M6) all exhibited the same MIC of 12.5 µM against *E. coli* ATCC 25922. MBC ranged from 13.9 to 26.4 µM (Table 1). MBC analysis determined that peptides Ib-M6 and Ib-M1 required between 1.8 and 2.1 times the MIC concentration to eliminate 99.9% of the bacteria, in contrast to Ib-M2, which demonstrated minimal variation between the MBC and MIC concentrations demonstrating it increased potency (Table 1). The peptides exhibited potent antimicrobial activity comparable to that of streptomycin, which is known to possess relatively strong antimicrobial activity against Gram-negative bacteria.

**Table 1:** Antimicrobial activity of Ib-M peptides against *E. coli* ATCC 25922.

Peptide	MIC µM Mean ± SD	MBC µM Mean ± SD
Ib-M1	12.5 ± 0	26.4 ± 9.8
Ib-M2	12.5 ± 0	13.9 ± 4.2
Ib-M6	12.5 ± 0	23.6 ± 4.2

MIC: Minimum inhibitory concentration. MBC: Minimum bactericidal concentration. The assays were performed in triplicate with three biological replicates. The MIC of streptomycin used as a biological activity control in *E. coli* was 6.2 µM.

**Fig 1:**
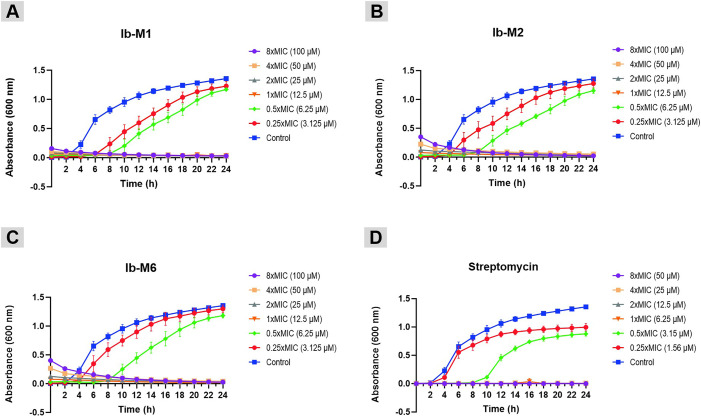
Growth kinetics of *E. coli* ATCC 25922 treated with Ib-M peptides. **(A)** Ib-M1. **(B)** Ib-M2. **(C)** Ib-M6. **(D)** Streptomycin was used as positive control. Data represent the mean ± SD of three independent experiments performed in triplicate. The MIC was recorded after 24 h of incubation and the MBC was registered after 18 **h.**

The growth kinetics of *E. coli* exposed to Ib-M concentrations ranging from 3.1 to 100 µM are presented in Fig 1. During the preparation of these assays, turbidity was observed in MHB when peptides were added at concentrations greater than 6.2 μM. Consequently, at the initiation of the experiments, an absorbance of 0.2 was observed in the kinetics of *E. coli* treated with the Ib-M1 peptide, and an absorbance of approximately 0.5 with the Ib-M2 and Ib-M6 peptides. However, the turbidity gradually decreased and after eight hours, these values remained constant (Fig 1).

As illustrated in Fig 1, *E. coli* growth was inversely proportional to the peptide’s concentration. Growth inhibition observed throughout the exposure period at concentrations of 12.5 µM and above. The three peptides demonstrated sub-inhibitory activity at 6.2 µM for up to ten hours post-exposure, during which time streptomycin, utilized as a positive control, also exerted its activity at a concentration of 3.1 µM (Fig 1). Additionally, at a sub-inhibitory concentration of 3.1 μM (0.25 × MIC), the Ib-M1 activity stayed stable for six hours, and then diminished, whereas Ib-M2 and Ib-M6 activity decreased after four hours.

### The antimicrobial activity of Ib-M against *E. coli* is maintained in the presence of K^+^, Na^+^ and Mg^+2^ cations

The Ib-M peptides maintained their inhibitory activity at approximately 1 or 2.2 times the MIC in the presence of K^+^, Na^+^, and Mg^+2^. In contrast, the Ib-M1 and Ib-M6 peptides exposed to Ca^+2^ exhibited decreased activity against *E. coli* ATCC 25922, resulting in an increase in their MIC of up to four times (Table 2). In comparison, the MIC value of Ib-M2 against *E. coli* in the presence of Ca^+2^ doubled (Table 2).

**Table 2:** Sensitivity of Ib-M peptides to physiological salts.

Peptide	MIC *E. coli* 25922 Control µM ± SD	MBC *E. coli* 25922 Control µM ± SD	NaCl (150 mM)		KCl (4.5 mM)		MgCl_2_ (2 mM)		CaCl_2_ (1 mM)	
			MIC µM ± SD	MBC µM ± SD	MIC µM ± SD	MBC µM ± SD	MIC µM ± SD	MBC µM ± SD	MIC µM ± SD	MBC µM ± SD
**Ib-M1**	12.5 ± 0	26.4 ± 9.8	25 ± 0	41.7 ± 12.5	15.6 ± 5.65	25 ± 0	25 ± 0	25 ± 0	50 ± 0	50 ± 16.7
**Ib-M2**	12.5 ± 0	13.9 ± 4.2	20.1 ± 6.27	23.3 ± 4.4	20.8 ± 12.5	26.4 ± 14.6	25 ± 0	23.6 ± 4.2	25 ± 0	37.5 ± 21.7
**Ib-M6**	12.5 ± 0	23.6 ± 4.2	26.4 ± 5.89	25 ± 0	27.1 ± 7.2	27.1 ± 7.2	27.8 ± 8.33	30.6 ± 11.0	50 ± 0	50 ± 25.7

MIC: Minimum inhibitory concentration. MBC: Minimum bactericidal concentration. Data were expressed as the mean in µM ± SD of three biological replicates (n = 9).

The MBCs ranged from 1.9 to 4 times the MIC value in the presence of the evaluated salts. The peptide with the highest efficacy when exposed to K^+^, Na^+^, and Mg^+2^ was Ib-M2, with values between 23.3 and 26.4 µM. When comparing MBC values between cations exposed and unexposed peptides revealed that the change did not exceed two dilutions (Table 2). These findings suggest that Ib-M peptides maintain their biological activity against *E. coli* despite exposure to cations.

### Ib-M peptides are associated with bacterial membrane disruption and alteration on bacterial ultrastructure

**Fig 2:**
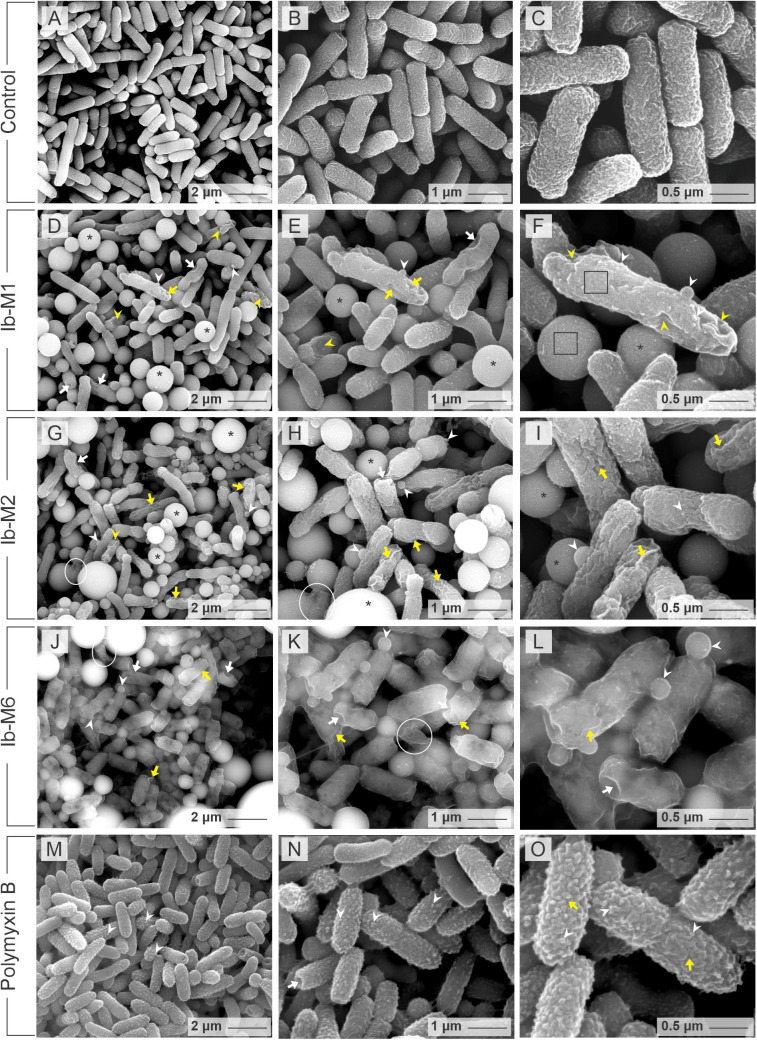
SEM morphology of Ib-M-treated *E. coli.* **(A-C)** *E. coli* without treatment. **(D-F)** Ib-M1. (**G-I**) Ib-M2. **(J-L)** Ib-M6. **(M-O)** *E. coli* treated with PMB. The alterations are indicated as follows: large bulges or spherical elements (asterisk), bulges in formation (white arrowhead), collapsed cells (yellow arrowhead), pores (white arrow), invaginations and deep wrinkles (yellow arrow), debris of cell membranes (white circles), and unchanged surface cells (black squares). All images are representative of three biological replicates with similar results.

The surface morphological alterations and ultrastructural changes observed in *E. coli* ATCC 25922 upon treatment with Ib-M1, Ib-M2, and Ib-M6 peptides and untreated bacterial controls were evaluated by transmission and scanning electron microscopy. During the preparation of bacterial cells for microscopy, we observed that following bacterial incubation with the Ib-M peptides and subsequent centrifugation, it was evident that the bacterial pellets acquired a brown color, in contrast to untreated bacteria or control PMB-treated bacteria, which showed a whitish color. Untreated *E. coli* ATCC 25922 cells exhibited a characteristic rod-like shape and intact membrane structure (Fig 2A, 2B, 2C, S3A, and S3B).

In contrast, marked alterations in bacterial size and shape were observed in cells exposed to the Ib-M1, Ib-M2, and Ib-M6 peptides at all exposure times (Fig 2D-L, S3C-H Fig). These changes were significantly different from those observed in cells treated with PMB, which was utilized as a positive control for membrane alterations in *E. coli* (Fig 2M-O and S3I and S3J Fig).

**Fig 3:**
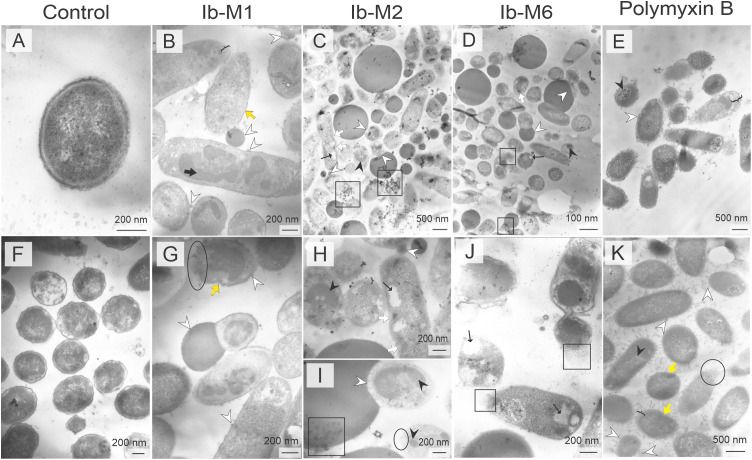
TEM morphology of Ib-M-treated *E. coli.* **(A, F)** *E. coli* without treatment. **(B, G)** Ib-M1. (**C, H, and I**) Ib-M2. **(D, J)** Ib-M6. **(E, K)** *E. coli* treated with PMB. The alterations are indicated as follows: cytoplasmic retraction (black bracket), bulges (white arrowhead), invagination (yellow arrow), electron-dense material (black arrow and arrowhead), lysed cells (black oval), cytoplasmic vacuoles (thin black arrow), cellular debris-lipid material (black square), and pore (white arrow). All images are representative of three biologically independent assays performed with similar results.

Exposed *E. coli* cells were smaller than unexposed cells (Fig 2D-L), exhibiting loss of their bacillary appearance, and some of them with elongated shape with lengths of 3–5 µm (Fig 2D). Additionally, spherical bacterial bulges of various sizes were identified along the bacterial cell surfaces and apical bacterial ends. The bulge surface structure was similar to that of bacterial outer membrane (Fig 2D–L). Small, medium, and large bulges were observed; with sizes ranging from less than 200 nm, 200–800 nm, and >800 nm, respectively (Fig 2D–L). The presence of bacterial bulges were consistent across all treatments with Ib-M peptides (Fig 2D-L) and they were observed in both SEM and TEM micrographs. Furthermore, Ib-M-treated cells exhibited an irregular cellular surface appearance, with pronounced invaginations (Fig 2D–L). Additionally, the bacterial cells displayed visible pore formation and contracted cell membranes (Fig 2D, 2E, 2G, and 2H). Cell membrane debris was also observed (Fig 2G, 2H, 2J, and 2K). In contrast, cells treated with PMB exhibited small blisters of uniform shape distributed across the bacterial surface (Fig 2M–O). The aforementioned alterations were also observed after 30 and 120 min of antimicrobial peptide exposure. Although, at 120 min, the damage was more pronounced in Ib-M-treated cells (S3D, S3F, and S3H Fig), as compared to PMB-treated cells (S3I and S3J Fig). TEM studies showed that the untreated *E. coli* controls maintained morphology, rod size, membrane structure and dense internal structure (Fig 3A and F). In contrast, bacteria treated with Ib-M peptides exhibited numerous alterations in the membranes and cytoplasm ultrastructure (Fig 3B–D and 3G–J).

At the evaluated concentrations, Ib-M-treated bacterial membranes displayed roughening and protrusions (Fig 3B–D and 3G–J), as well as increased separation of the cytoplasmic and outer membranes, as evidenced by the light surrounding each cell or blurred appearance (Fig 3B, 3G, 3H, and 3I). A loss of membrane continuity was observed along with its disruption, characterized by bulges of varying sizes, some of which were separated from the cell, while others remained attached (Fig 3B-D and 3G-J). The bulges exhibited similar electron densities (Fig 3B-D and 3G-J). Additionally, bacterial membranes had pores in all Ib-M peptide treatments (Fig 3C, 3D, and 3H). In the bacterial cytoplasm; electron-dense material, vacuoles of various sizes, retraction of the cytoplasm, loss of cytoplasmic content, and sparse cytoplasmic distribution were observed (Fig 3B-J). Bacterial clumping was also noted. In contrast, PMB-treated bacteria exhibited only small blisters on their outer membrane surfaces (Fig 3E and 3K).

### Ib-M1 peptide has low cytotoxic effect on human cell lines

Among all three peptides evaluated, the Ib-M1 peptide demonstrated the lowest cytotoxicity against the non-tumor cell line NCM460, as determined by the MTT assay. The IC_50_ was 120 µM, which corresponds to a SI of 9.6 (Table 3). SI was defined as the ratio of the IC_50_ (NCM460) to the *E. coli* MIC, and it is an important parameter to determine when considering potential applications. This index was also calculated as the ratio of the IC_50_ of non-tumor cell line to tumor cell line.

**Table 3:** Cytotoxic concentrations of Ib-M peptides in mammalian cells calculated based on MTT assays.

Peptide	NCM460		SW480		Caco2	
	Mean IC_50_ (µM) ± SD	SI	Mean IC_50_ (µM) ± SD	SI	IC_50_ (µM) ± SD	SI
Ib-M1	120 ± 8.7	9.6	> 200	–	106 ± 3.5	1.1
Ib-M2	38.6 ± 3.2	3.0	78 ± 4.0	0.4	29.3 ± 0.3	1.3
Ib-M6	39.3 ± 3.5	3.1	54.1 ± 3.6	0.7	27 ± 1.6	1.5

IC_50_: Inhibitory concentration 50. SI: Selectivity index. Selectivity index calculation: non-tumor cell line [IC_50_]/MIC *E. coli* ATCC 25922 (12.5 µM). Non-tumor cell line [IC_50_]/tumor cell line IC_50_. The data correspond to the average IC_50_ of the biological replicates.

**Fig 4:**
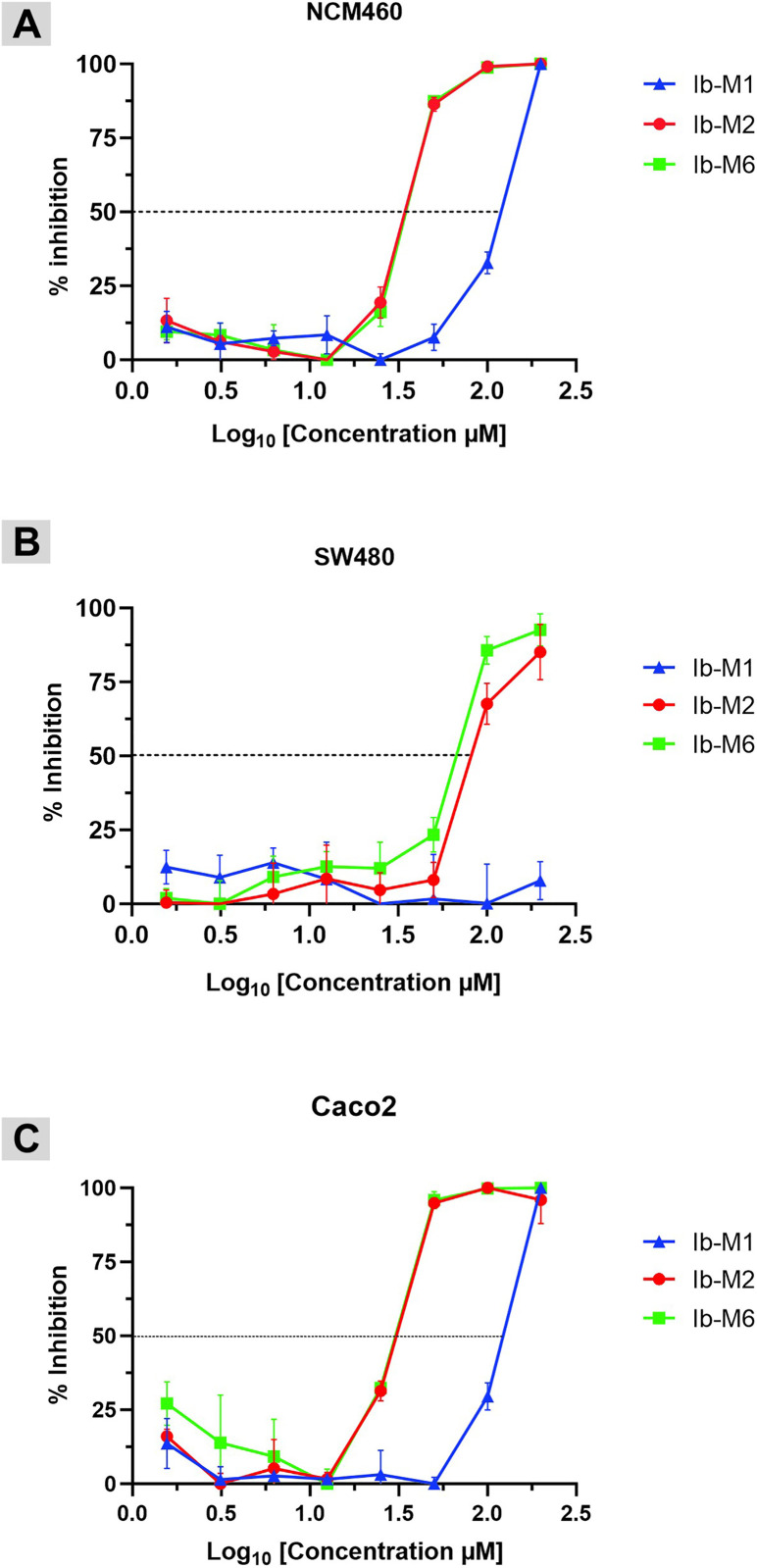
Cytotoxic activity of Ib-M peptides in mammalian cells. The cytotoxic activities of the peptides were measured in **(A)** NCM460 non-cancerous, **(B)** SW480, and **(C)** Caco2 cancer cell lines using MTT assay. Triton X-100 (1%) was utilized as a positive control for cytotoxicity. The data represent the mean ± SD of a representative assay (n = 3). The dotted line corresponds to the IC_50_ value or 50% cytotoxicity, which defines the pharmacological potency of the molecule.

In contrast, the concentrations of peptides Ib-M2 and Ib-M6 required to inhibit the metabolic activity of these cells were 3.1 times higher than the MIC against *E. coli* ATCC 25922 (12.5 µM). The IC_50_ values of the peptides Ib-M2 and Ib-M6 in Caco2 cells were less than 30 µM, while those in SW480 cells ranged between 54 and 78 µM (Table 3). When the SW480 cell line was treated with the Ib-M1 peptide, it was not possible to obtain the IC_50_ at the evaluated concentrations; and for the Caco2 cell line, the required concentration exceeded 100 μM (Table 3). Ib-M2 and Ib-M6 demonstrated an SI less than 1.0 in SW480 cells, and all three peptides were active in Caco2 cells with values between 1.13 and 1.45. The SI of these peptides indicates higher cytotoxicity against tumor cells compared to non-tumor cells (Table 3). The SI of 9.6 obtained for Ib-M1 suggests that this peptide may be selective for *E. coli*, as it exhibits low cytotoxicity to both non-tumor and tumor cells (Table 3). Conversely, the SI values obtained with the other two peptides in these cells were 3.1 times lower, supporting the assertion regarding Ib-M1. Comparison of the IC_50_ values obtained following the application of the Ib-M peptides to NCM460 and Caco2 cells revealed significant differences between the Ib-M1 peptide and Ib-M2 (p* *< 0.0001) and Ib-M6 (p* *< 0.0003), but not between Ib-M2 (p* =* 0.9940) and Ib-M6 (p* =* 0.7523 for each cell line). The paired t-test analysis of the treatment of SW480 cells with the Ib-M2 and Ib-M6 peptides yielded a statistically significant difference (p* = *0.0050). The Ib-M peptides exhibited a dose-dependent cytotoxic effect, with greater inhibition of the metabolic activity of the mammalian cells observed at higher peptide concentration values. Fig 4 illustrates the cytotoxic effect of the peptides on the three cell lines after normalization and adjustment of concentrations to their logarithms.

The IC_50_ of the NCM460 and Caco2 cells was achieved by Ib-M1 peptide at concentrations of Log_10_ [2.07 µM] and Log_10_ [2.02 µM], respectively (Fig 4A and 4C). For SW480 cells, no cytotoxicity was observed at the evaluated concentrations (Fig 4B). In contrast, cytotoxicity among NCM460 cells was observed at Log_10_ [1.58 µM] and Log_10_ [1.59 µM] concentrations of Ib-M2 and Ib-M6 peptides, respectively (Fig 4A).

### The Ib-M peptides have hydrophobic, amphipathic, and cationic properties

To have an insight into the mechanisms of antimicrobial activity at the molecular level, the Ib-M peptides were evaluated for their unique physico-chemical properties. All peptides analyzed had hydrophobic, amphipathic, and cationic properties based on *in silico* predicted analysis. With respect to the index of polarity, the Ib-M1 and Ib-M6 peptides had higher polarity based on an index of 1.5 compared to 1.22 for Ib-M2 (Table 4).

**Table 4:** Distribution of amino acids in Ib-M peptides.

Peptide	Hydrophobic face	Polar residues n/%	Non-polar residues n/%	^a^Residues index P/NP	Charged residues number	Non-charged residues number	Aromatic residues number	Special residues number
Ib-M1	MWP	12/60	8/40	1.50	7 (R), 1(E)	4 (G)	3 (W)	1(P)
Ib-M2	MWP	11/55	9/45	1.22	7 (R), 1(E)	3 (G)	4 (W)	1(P)
Ib-M6	MWWW	12/60	8/40	1.50	7 (R), 1(E)	4 (G)	4 (W)	0

Data were obtained using Heliquest software. ^a^Index calculated between polar (P) and nonpolar (NP) residues. M: methionine; W: tryptophan; P: proline.

Peptides Ib-M1 and Ib-M2 exhibited the same hydrophobic face, with MWP residues in positions 7, 18, and 11. In contrast, Ib-M6 exhibited MWWW residues at positions 16, 9, 2, and 20 (Table 4). A detailed analysis of the amino acid composition of the peptides in Table 4 illustrates the number of equivalent charged residues between the peptides, with seven R and one E (glutamic acid) residue. The three peptides exhibited percentages of polar residues between 55 and 60%, and non-polar residues between 40 and 45% (Table 4). The hydrophobic radii of these peptides, obtained from https://aps.unmc.edu/prediction, corresponded to 35, 40, and 40% for each peptide (Ib-M1, Ib-M2, and Ib-M6, respectively). Additionally, predictions of the physicochemical parameters of the Ib-M peptides were obtained from the Heliquest, DBAASP, and Pepcalc servers (Table 5). Ib-M6 demonstrated higher values for the hydrophobic and normalized moment (Table 5).

**Table 5:** Physicochemical properties of the Ib-M peptides.

Peptide	Hydrophobicity <H > ^a^	Normalized Hydrophobicity	Hydrophobic moment µH^a^	Normalized Hydrophobic Moment	Isoelectric Point	Penetration Depth	Tilt Angle	Propensity to Disordering	Linear Moment	Angle Subtended by the Hydrophobic Residues	Amphiphilicity Index^b^	Propensity to PPII coil
Ib-M1	0.234	−0.13	0.179	0.44	12.50	23	134	−0.57	0.19	60	1.96	0.94
Ib-M2	0.346	−0.24	0.174	0.38	12.50	17	99	−0.58	0.21	60	2.31	0.97
Ib-M6	0.310	−0.07	0.429	0.50	12.50	17	99	−0.56	0.22	60	2.31	0.88

^a^Hydrophobicity values <H> indicate the total hydrophobicity (sum of all residue hydrophobicity indices) divided by the number of residues. The hydrophobic moment (μM) was used to analyze the amphipathicity of the peptides using https://heliquest.ipmc.cnrs.fr/cgi-bin/ComputParams.py. ^b^The amphiphilicity index was obtained by dividing the total amphiphilicity of the sequence (sum of the amphiphilicity of each residue according to the index database (AAindex)) by the total residues [56,57]. Physicochemical properties were calculated using https://dbaasp.org/tools?page=property-calculation and https://pepcalc.com/notes.php?al. PPII: polyproline II.

The Ib-M peptides exhibited an isoelectric point of 12.5 and a hydrophobic residue angle of 60°. The calculated amphiphilicity index reflects the peptide’s ability to interact with both hydrophobic and hydrophilic environments. The physicochemical analysis revealed amphiphilicity index values ranging from 1.96 to 2.31. Additionally, the Ib-M peptides showed a propensity to form polyproline II (PPII) helices, with Ib-M2 and Ib-M1 displaying the highest values, 0.97 and 0.94, respectively (Table 5). These PPII structures are stabilized by intramolecular cation-π and π-π interactions between aromatic (W) and cationic (R) residues, potentially contributing to helix stability. Notably, the peptides showed no tendency to aggregate *in vitro* (aggregation score = 0) (Table 5). The chemical structure drawn at https://pepdraw.com/ illustrates the characteristics and distribution of the residues (S4 Fig). Ib-M1, Ib-M2 and Ib-M6 peptides contain seven R residues with an array of spatial distributions. The guanidinium group of R is important for interactions with negatively charged groups or π-electrons-rich aromatic molecules (S4 Fig). In Ib-M1, a P residue was observed between two G; while in Ib-M2, it was located between an R and a G (S4A and S4B Fig). The Ib-M6 peptide shares structural similarity with Ib-M2, differing mainly in the position of the central R residue and the C-terminal end (S4B and S4C Fig). The C-termini of the three peptides are amidated to enhance structural stability (S4 Fig). The asymmetric distribution of polar and nonpolar residues results in imperfect amphipathicity, which may contribute to the biological activity of these peptides.

### The Ib-M peptides secondary structure contains two α-helices

**Fig 5:**
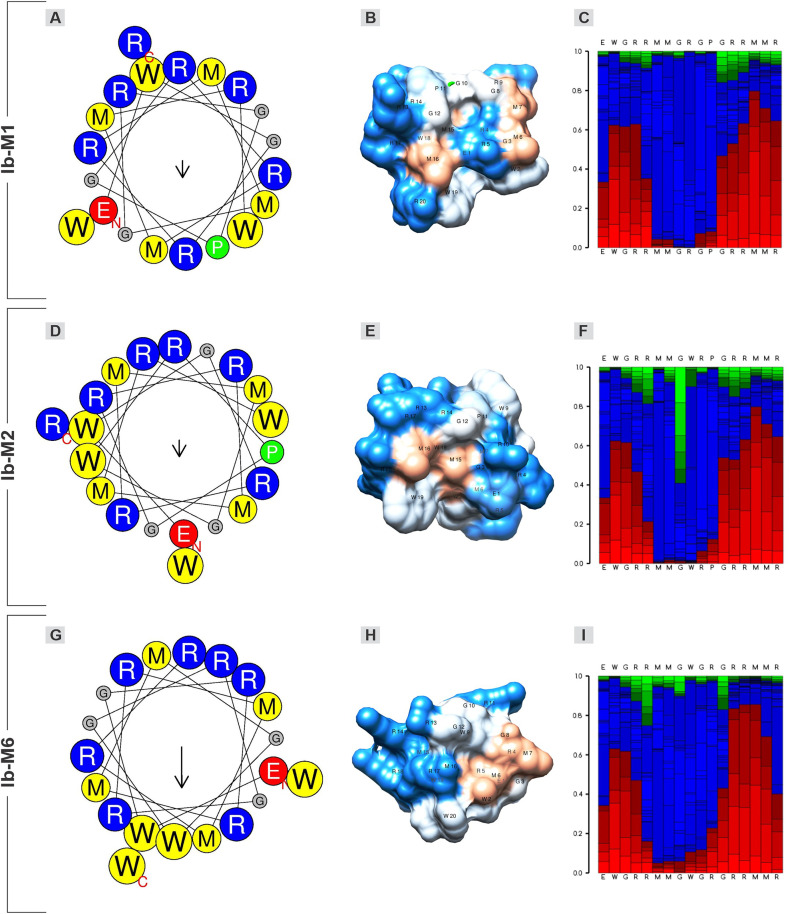
Helical wheel, electrostatic potential surface, and local structure prediction of Ib-M peptides. The simulations of each peptide are presented in horizontal rows. (**A, D, G**) Net wheels were plotted utilizing the Heliquest. Blue: positively charged residues. Red: negatively charged residues. Yellow: hydrophobic residues. Gray: noncharged residues. Green: **P.** The hydrophobic moment (µH) of the Ib-M peptides is indicated at the center of each wheel. The longest arrow indicates the highest hydrophobic moment. In the figure: Ib-M6 > Ib-M1 > Ib-M2 (0.429, 0.179, 0.174, respectively). (**B, E, H**) Electrostatic surface predictions. Positive (hydrophilic), hydrophobic, and neutral residues are indicated by blue, orange, and light gray, respectively. (**C, F, I**) Prediction of the local structure profile of the peptides. Red: helical. Green: extended. Blue: coil.

The α-helical wheel plot of the Ib-M peptides was predicted using the online tool available at https://heliquest.ipmc.cnrs.fr/cgi-bin/ComputParams.py. The calculation of mean hydrophobic moments, which serves as a numerical estimate of peptide structure amphipathicity, indicated similarities in the direction of this vector among the three peptides, but different magnitudes for Ib-M6 (Fig 5A, 5D, and 5G). The helical wheel projections facilitated the visualization of an interrupted amphipathic configuration from the spatial separation of the polar residues by a hydrophobic core (Fig 5A, 5D, and 5G).

**Fig 6:**
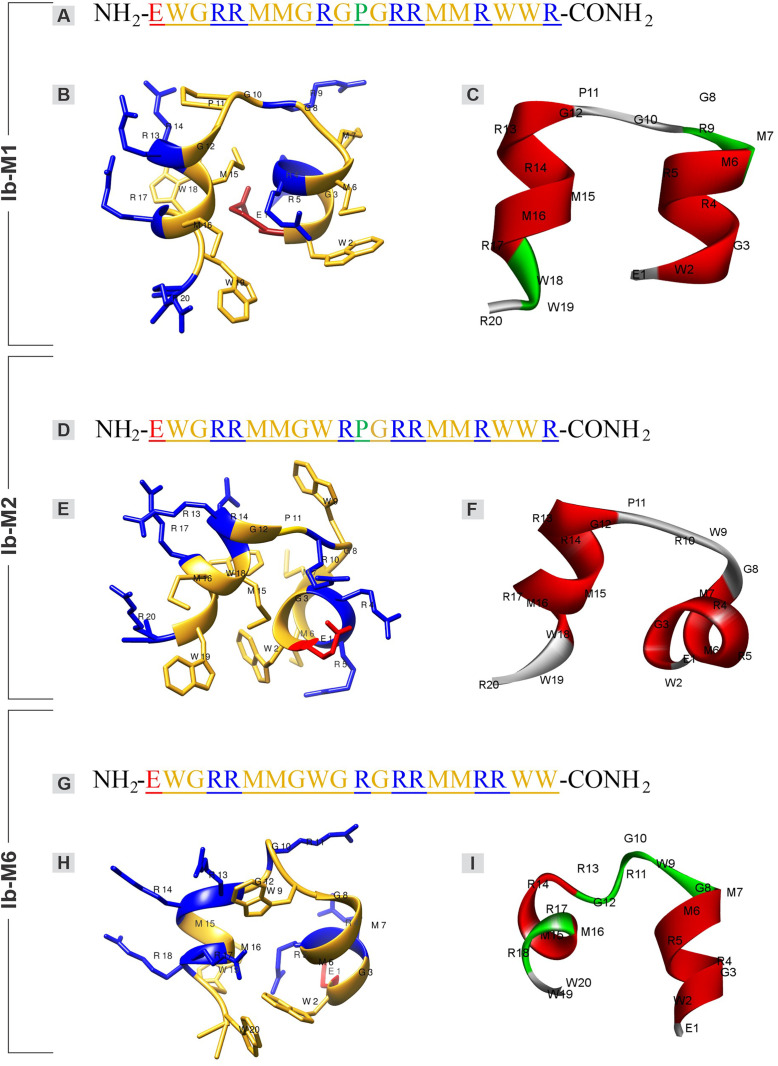
The secondary structure of the Ib-M peptides. The amino acid sequences and the three-dimensional structure projections of Ib-M peptides were visualized using Chimera USFC and Discovery Studio. (**A, B, and C**) Ib-M1. (**D, E, and F**) Ib-M2. (**G, H, and I**) Ib-M6. Left column: Blue: positively charged polar residues **(R)**; Yellow: hydrophobic (W, M, P) and neutral polar **(G)**; Red: negatively charged polar, glutamic acid **(E)**. Right column: Red: α helix; Green: twist or turn; Gray: coil.

This configuration facilitated an imperfect amphipathic structure, as illustrated in the electrostatic potential surface distribution, wherein Ib-M peptides exhibited patches of cationicity distributed asymmetrically (Fig 5B, 5E, and 5H). Despite the similarity in the peptide sequences, the distribution of the electrostatic potential surface differed. Furthermore, the surface model revealed that Ib-M6 possessed the most defined hydrophobic face, which was distinctly separated from the hydrophilic face (Fig 5B, 5E, and 5H). The local structure calculated in PEP-FOLD3.5 also demonstrated the secondary structure distribution of the peptides (Fig 5C, 5F, and 5I). Five predictive models of the three-dimensional structure of the Ib-M peptides were obtained from the PEP-FOLD3.5 server (https://mobyle.rpbs.univ-paris-diderot.fr/cgi-bin/portal.py#forms::PEP-FOLD3). The optimal model for each peptide structure was selected based on the MolProbity score, clash score, and percentage of residues in favored regions of the Ramachandran plot (S2 Table), using the Structure Assessment web server on SWISS-MODEL (https://swissmodel.expasy.org/assess). The Ramachandran plot assesses protein conformation based on backbone angles, while MolProbity offers detailed all-atom stereochemical validation. The Ib-M peptides were found to be composed of a non-polar (hydrophobic) core comprised of W, M, G (neutral), and/or P, with positively charged residues (R) surrounding the hydrophobic core (Fig 6A, 6D, and 6G).

The secondary structure simulation revealed two α-helices separated by coils or turns in all three peptides (Fig 6B, 6E, and 6H). The first α-helix initiated at the N-terminus and it was comprised of five amino acids in Ib-M1 and six in the other two peptides (Fig 6B, 6E, and 6H). In Ib-M1, the first α-helix was separated by a turn and a coil composed of M7, G8, R9, G10, and P11 (Fig 6B and 6C). The coil of Ib-M2 was composed of G8, W9, R10, and P11 (Fig 6E and 6F), and it differed slightly from Ib-M1. In Ib-M6, the middle segment consisted of a coil formed by G8, W9, G10, R11, and G12; which separated the first and second α-helices (Fig 6H and 6I). The P11 residue, present in Ib-M1 and Ib-M2, contributes to the division of the sequence into two α-helices. This residue disrupts the helix secondary structure because its secondary amino group generates a turn (Fig 6B and 6E). In Ib-M6, this turn was also observed and attributed to the G residues at positions 8, 10, and 12 (Fig 6H). The second α-helix of peptides Ib-M1 and Ib-M2, towards the C-terminus, is composed of residues G12, R13, R14, M15, M16, and R17, whereas in Ib-M6, this α-helix is shorter (residues 13–16). Spirals or turns were observed at the C-termini of all three peptides (Fig 6C, 6F, and 6I).

### Circular Dichroism (CD)

Predictions of helix content using the BeStSeL program indicated that Ib-M1 exhibited the highest α-helix content (73.9% in sodium dodecyl sulfate -SDS), followed by Ib-M6 (51.5%) and Ib-M2 (40.3%) (Table 6). In a simulated membrane environment using trifluoroethanol (TFE), the majority of the peptides demonstrated α-helical structures, albeit with a lower proportion compared to the SDS environment. Specifically, the α-helix content of Ib-M1 was 4.2%, which was lower than that of Ib-M2 (15.6%) and Ib-M6 (8.2%) (Table 6). These findings were consistent with the CD spectra previously obtained by our research group [27].

**Table 6:** Predictions of helix content (%) in Ib-M peptides with BeStSeL.

Peptides	30 mM SDS			30% TFE			10 mM Tris-HCl			Water		
Structure	Ib-M1	Ib-M2	Ib-M6	Ib-M1	Ib-M2	Ib-M6	Ib-M1	Ib-M2	Ib-M6	Ib-M1	Ib-M2	Ib-M6
^a^Helix-1	73.9	36.8	38.6	0	6.7	5.2	0	0	0	0	0	0
Helix-2	0	3.6	12.9	4.2	8.9	3	4.3	6.4	0	2	4.1	1.2
Anti-1	0	0	0	3	4.8	0	2.1	0	6.9	0	0	2.6
Anti-2	3.1	7.4	0	12.8	10.4	9.4	17.7	11.9	13.1	11.5	5.9	6.2
Anti-3	0	0	0	10.6	19.5	18.3	25	22.9	35.4	26.4	24.7	24
Parallel	0	0	0	7	3.9	1.1	0	0	0	0	0	0
Turn	0	6.9	7.5	8.3	15.8	15.4	8.9	18	24.7	11	17.5	20
^b^Others	23.1	45.4	41	54	30.1	47.6	42	40.8	19.9	49.1	47.8	46

^a^Helix-1, helix-2, anti-1, anti-2, and anti-3 indicate regular α-helices, distorted α-helices, left-twisted β-strands, relaxed β-strands, and right-twisted β-strands, respectively.

^b^Others: 3–10 helix, Pi-helix, β-bridge, folds, loops, irregular, and invisible regions of the structure. Predictions were performed using BeStSel software (https://bestsel.elte.hu/index.php).

For water, the predictions ranged from 1.2% for Ib-M6 to 4.1% for Ib-M2. In Tris-HCl, the results were 4.3% for Ib-M1 and 6.4% for Ib-M2 (Table 6). These correlated with the *in silico* simulation results obtained using PEP-FOLD3.5, where the interactions between amino acids to form hydrogen bonds were predicted (Fig 6). These results suggest that Ib-M peptides can acquire a stable three-dimensional secondary structure in the presence of the hydrophobic *E. coli* cell membrane. Furthermore, the helicity formed in SDS can be attributed to an increase in cation-π interactions between the R and W residues of the peptides.

### Ib-M peptides interact with bacterial membranes

The PPM 3.0 server provides the inclination angle and number of residues of the peptides embedded in the bacterial cell membranes. Table 7 summarizes the parameters obtained from the simulations of Ib-M peptides in PPM 3.0. The positively charged Ib-M peptides interacted with the anionic lipids of the cytoplasmic membrane with inclination angles ranging from 13° to 100°, with Ib-M1 and Ib-M2 exhibiting the most vertical orientation.

**Table 7:** Parameters obtained from Ib-M simulations in PPM 3.0.

Peptide	Depth/hydrophobic thickness (Å)	ΔG transfer (Kcal/mol)	Tilt angle (°)	Membrane-embedded residues (hydrophobic core)	Residues	Total
Ib-M1	3.8 ± 0.9	−4.4	72° ± 11°	2, 3, 6	W, G, M	3
Ib-M2	3.3 ± 0.8	−4.5	100 ± 7°	2, 9	W, W	2
Ib-M6	3.7 ± 1.7	−2.9	13° ± 16°	9	W	1

Simulations were performed using the PPM 3.0 online server (https://opm.phar.umich.edu). Gibbs Transfer Energy (ΔG) is required for the transfer of peptides from aqueous solutions to membranes. W: Tryptophan; G: Glycine; M: methionine.

**Fig 7:**
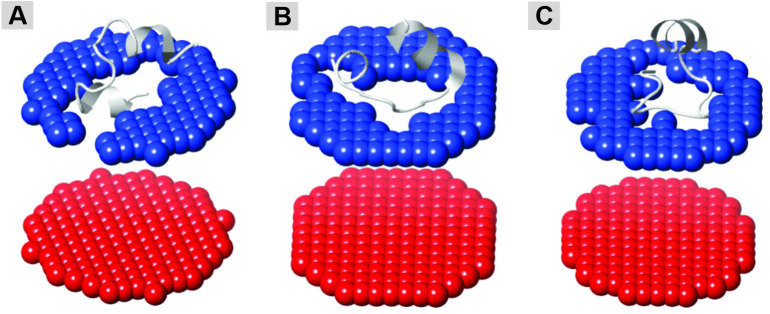
Predicted orientation of Ib-M peptides in the membranes of Gram-negative bacteria. Vertical images of the peptide orientations were obtained using Jmol 3D, a visualization tool from the OPM database of the PPM 3.0 server. **(A)** Ib-M1. **(B)** Ib-M2. **(C)** Ib-M6.

The Ib-M1 peptide interacted with the membrane at an orientation of 72 ± 11°, with residues of W, G, and M (positions 2, 3, and 6) incorporated into the hydrophobic core of the membrane at a depth of 3.8 ± 0.9 Å and a ΔG transfer of −4.4 Kcal/mol (Table 7). For Ib-M2, the inclination was 100 ± 7°, and the incorporated residues were W2 and W9 (Table 7). Conversely, Ib-M6 exhibited the lowest inclination, at 13 ± 16°, and only W9 was incorporated into the membrane (Table 7); with this W9 residue being part of the hydrophobic face, as evidenced by helical wheel simulations using the Heliquest software (Fig 5G). The observed antibacterial activities of these peptides are likely correlated with the findings of these predictions. Peptide inclination angles and disruptions occurring in the membrane when peptides interacted with phospholipids are indicated (Fig 7). Prediction of the orientation of Ib-M peptides revealed that only the termini of each peptide interacted with the membrane and were incorporated into the simulated membrane.

### Ib-M peptide interaction with LPS and outer membrane is associated with membrane disruption

**Fig 8:**
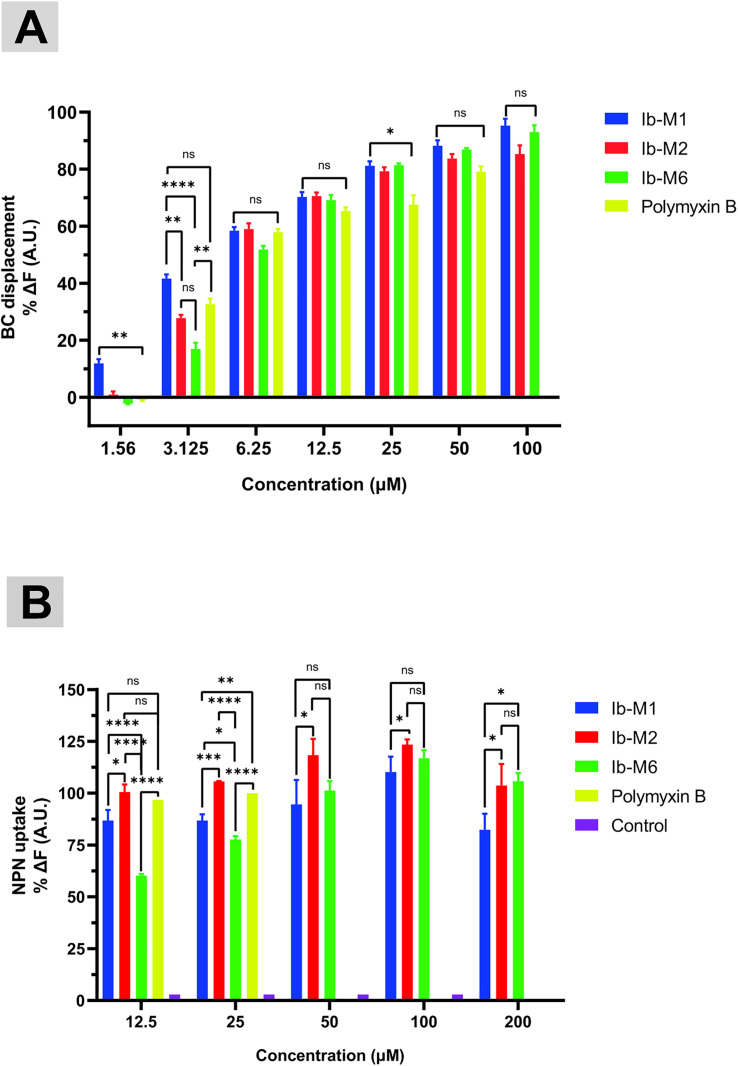
Interaction of Ib-M peptides with LPS and permeabilization of the *E. coli* outer membrane. (A) Determination of BODIPY-TR cadaverine displacement from LPS by Ib-M peptides after 60 min of exposure. PMB was utilized as a positive control. Values are expressed as mean ± SD of three independent assays. Statistical analysis was performed using ANOVA with Tukey’s multiple comparison test. (B) NPN fluorescence measurement upon exposure of *E. coli* ATCC 25922 to Ib-M1 peptide. PMB was used as the positive control at a concentration of 12.5 µM. The negative control was comprised of bacteria without the addition of peptides. Measurements were obtained at minute 60. The mean ± SD of a representative assay is presented. Significant differences were established and are denoted as *p < 0.05, **p < 0.01, ***p < 0.001, ****p < 0.0001; ns indicates no significance.

Addition of Ib-M peptides to the LPS/BC mixture displaced BC, resulting in an increase in fluorescence, which demonstrates effective competition of peptides with BC for high binding to LPS (Fig 8A).

As shown in Fig 8A, at 12.5 µM (1 × MIC), Ib-M peptides displaced approximately 70% of BODIPY-TR cadaverine from LPS. When the concentration of Ib-M peptides was increased to 100 µM (8 × MIC), the displacement percentages were 95.3%, 93%, and 85.3% for Ib-M1, Ib-M6, and Ib-M2, respectively. These findings suggest that Ib-M peptides bind to LPS in a concentration-dependent manner, with binding strength comparable to that of PMB, which was utilized as a positive control (p* *> 0.05). Furthermore, at 25 and 50 µM peptide concentrations, Ib-M peptides bound to LPS at rates exceeding 88.1%, whereas PMB exhibited a binding rate of only 79% under identical conditions. However, significant differences were observed when comparing the effects of Ib-M peptides upon LPS to those of PMB at 25 µM (p* *< 0.05) (Fig 8A). At sub-MIC concentrations of 6.2 and 3.1 µM, Ib-M peptides displaced BC with fluorescence intensities ranging from 16.9% to 59% (Fig 8A). S5 Fig shows a comparison of the peptides at their respective concentrations. As depicted in Fig 8B, treatment of *E. coli* ATCC 25922 with Ib-M peptides at concentrations ranging from 12.5 to 200 µM resulted in a significant increase in NPN fluorescence intensity after 60 minutes, whereas the fluorescence of *E. coli* treated with streptomycin remained unaltered. Ib-M2 was associated with the highest permeability within the 12.5 to 100 µM range, with significant differences compared to Ib-M1 (p* *< 0.05). Cells treated with Ib-M6 demonstrated a dose-dependent increase in NPN fluorescence from 12.5 to 100 µM (Fig 8B). The increase in NPN fluorescence intensity after exposure to the MIC values (100, 200, and 200 µM) of Ib-M2, Ib-M6, and Ib-M1 peptides was 123.4%, 105.8%, and 82.3%, respectively. These results indicate that Ib-M peptides exhibit an outer membrane permeabilizing ability in *E. coli*, similar to the effect of PMB at 12.5 and 25 µM. However, at 200 µM, the Ib-M1 peptide slightly reduced the outer membrane permeabilizing effect (Fig 8B). At a sub-MIC concentration of 50 µM, NPN uptake by cells treated with Ib-M peptides was slightly lower than at 100 µM (Fig 8B). At sub-MIC concentrations (12.5 and 25 µM), Ib-M1 and Ib-M2 showed similar permeabilization effects. However, with Ib-M6, NPN fluorescence intensity increased from 60.3% to 77.8%. Fluorescence measurements taken at two-minute intervals revealed a sustained trend of membrane permeabilization throughout the exposure period (S6A-C Fig). However, at the tested peptide concentrations, a slight reduction in permeabilization was observed for up to 20 minutes, after which fluorescence levels remained stable for the next 40 minutes, similar to the behavior of PMB (S6D Fig). Streptomycin, utilized as a negative control, did not induce fluorescence levels above those of untreated cells (S6E Fig).

### Exposure of *E. coli* cells to Ib-M peptides results in permeabilization and changes in the inner membrane potential

**Fig 9:**
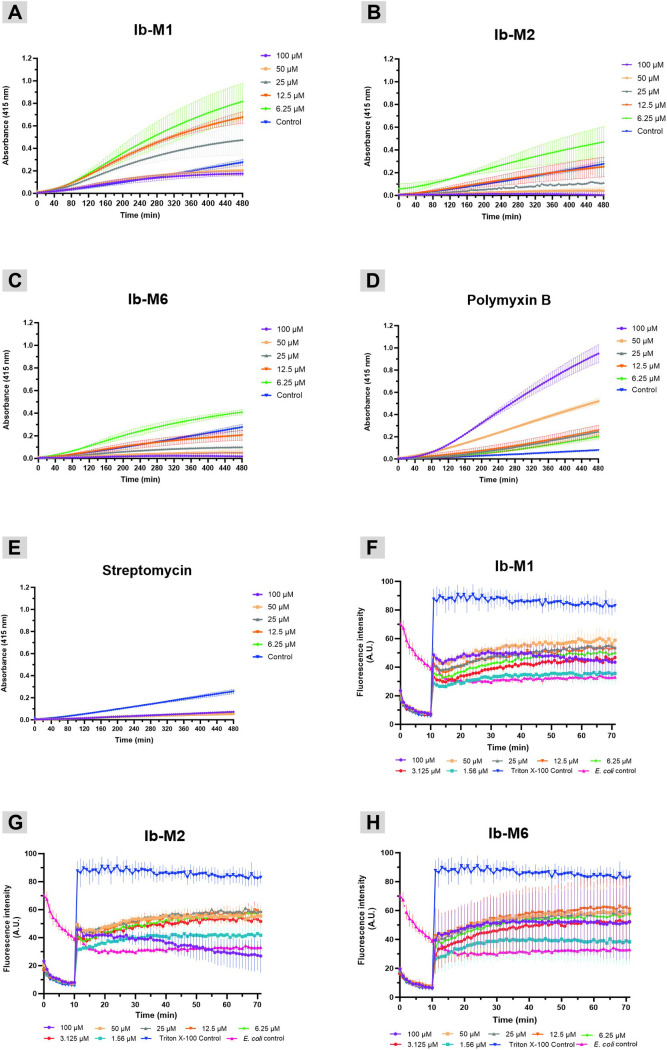
*E. coli* inner membrane permeability and inner membrane potential changes. Inner permeabilization of *E. coli* ML35. (**A**) Ib-M1. (**B**) Ib-M2. (**C**) Ib-M6. Measurements were obtained every five min for 8 h. PBS without the addition of ONPG served as the negative control. (**D**) PMB and (**E**) streptomycin were employed as the positive and negative controls, respectively. Time-dependent cytoplasmic membrane depolarization of *E. coli* ATCC25922 induced by (**F**) Ib-M1, (**G**) Ib-M2, and (**H**) Ib-M6; as determined using the membrane potential-sensitive fluorescent dye, DiSC_3_ [5]. Dye release was monitored by measuring fluorescence at an excitation wavelength of 620 nm and an emission wavelength of 670 nm. Triton X-100 was used as the positive control. Data are expressed as mean ± SD of three independent assays.

Upon treatment of *E. coli* ML35 with Ib-M1 peptide, membrane permeabilization commenced 60 minutes post-exposure, as evidenced by an increase in β-galactosidase activity. Maximum permeability was observed at a ½ MIC concentration of 6.2 μM, followed by permeability at the MIC value of 12.5 μM, with minimal permeabilization observed at 25 μM (Fig 9A, 9B, and 9C). No permeabilizing effect was detected at other concentrations up to 8 hours post-exposure.

**Fig 10:**
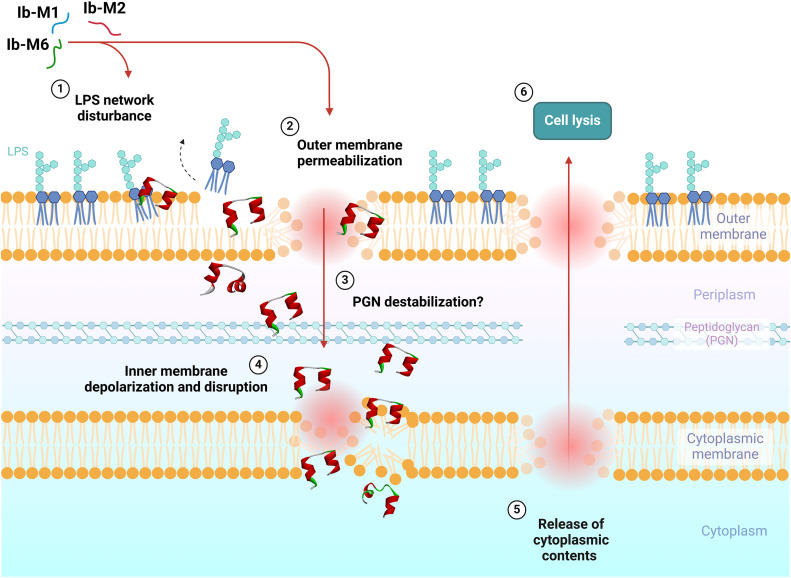
Proposal action mechanisms of Ib-M peptides. Schematic illustration depicting the impact of Ib-M1, Ib-M2, and Ib-M6 on the cell membranes of Gram-negative bacteria. Ib-M peptides demonstrate disruptive effects on bacterial outer and inner membranes.

*E. coli* treated with Ib-M2 and Ib-M6 peptides exhibited comparable effects at ½ MIC (6.2 μM). However, at concentrations exceeding 12.5 μM, the absorbance values were either equivalent to or lower than those of the control (Fig 9B and 9C), indicating a diminished permeabilizing effect compared to that of Ib-M1. Anticipated results were observed in the control treatments. Bacteria treated with PMB (positive control) demonstrated permeabilization at concentrations ranging from 6.2 to 100 μM (Fig 9D), whereas no β-galactosidase activity was detected in bacteria exposed to streptomycin (negative control) (Fig 9E). Ib-M peptides induced depolarization of the inner membrane of *E. coli* ATCC 25922 after ten minutes of fluorescence stabilization at concentrations ranging from 100 μM to 1.5 μM. Fluorescence increased approximately three- to five-fold during the initial 30 minutes and remained relatively stable for the next 40 min (Fig 9F, 9G, and 9H). Depolarization of the *E. coli* cytoplasmic membrane induced by Ib-M peptides was less pronounced than that observed with Triton X-100, which served as the positive control. Moreover, exposure to 100 μM Ib-M peptides resulted in a gradual decrease in fluorescence, commencing at approximately 30 min and persisting until the conclusion of the measurement period. This effect was most pronounced for Ib-M2, where the fluorescence diminished below that of untreated *E. coli* at 55 minutes (Fig 9G). We illustrated our perspectives using a schematic representation, wherein Ib-M1, Ib-M2, and Ib-M6 cause LPS destabilization. Ib-M peptides can directly penetrate both membranes and affects the outer and inner membrane. These findings highlighted the action mechanism of Ib-M peptides in *E. coli* ATCC 25922 (Fig 10).

## Discussion

To the best of our knowledge, this is the first study reporting evidence on the possible mechanism of action of Ib-M1, Ib-M2, and Ib-M6 peptides against *E. coli* ATCC 25922. This study confirms the antimicrobial and bactericidal activity of Ib-M peptides on microdilution assays, and it demonstrated by electron microscopy studies the severe disruption of bacterial membranes leading to bacterial demise. Furthermore, studies at the molecular level, including elucidation of physicochemical properties and secondary structure of these peptides, provide clues on the possible role of these peptides not only in the outer and inner bacterial membranes permeabilization but, perhaps more importantly, in the interactions of these peptides with LPS and the subsequent disruption of the outer membrane of the *E. coli* ATCC 25922 bacterial model.

The strong antimicrobial activity exhibited by Ib-M1, Ib-M2, and Ib-M6, as evidenced by low MIC values (12.5 µM), is consistent with previously reported AMPs that are rich in cationic and hydrophobic residues [20,58–60]. MIC and MBC results demonstrated that the Ib-M peptides maintained anti-*E. coli* activity by inhibiting its growth in a dose-dependent manner. Growth kinetics confirmed that Ib-M peptides act as potent bactericidal agents with sustained efficacy for over 24 h. These findings confirm prior work from our group, on the antibacterial activity of Ib-M peptides against both reference and clinical *E. coli* strains [27,28]*.* Their high hydrophobicity, per HPLC retention times (10.692–14.436 min), further supports their ability to interact with lipid bilayers, an essential feature of membranolytic peptides [61–63].

A key aspect of AMPs is their ability to retain their antibacterial activity under physiological salt conditions. In our study, Ib-M peptides maintained their bactericidal effects in the presence of Na ⁺ , K ⁺ , and Mg² ⁺ , which is in contrast with studies reporting decrease AMP efficacy exposed to these cations [64]. This salt tolerance may be attributed to the high R residues density, that may help preserve peptide–membrane interactions under ionic stress. In addition to R residues, the strategic position of W residues may facilitate the peptide’s passage into the membrane hydrophobic core, thereby counteracting the effects of these cations [65]. On the other hand, Ca² ⁺ impairs the activity of Ib-M1 and Ib-M6, likely due to the strong Ca^+2^ binding affinity for LPS and membrane-stabilizing properties [65,66]. These findings highlight the importance of ionic conditions on the predicted *in vivo* antibacterial efficacy of these peptides.

Regarding cytotoxicity, Ib-M1 exhibited minimal toxicity in non-tumor human colon cells, with an IC_50_ of 120 µM and a selectivity index of 9.6, suggesting a favorable therapeutic window. These findings are consistent with those reported by Prada [27] in Vero cells Notably, Ib-M1 showed no toxicity in Caco2 tumor cells, even at concentrations above 106 µM, further reinforcing its selectivity for bacterial over mammalian membranes. In contrast, Ib-M2 and Ib-M6, displayed greater cytotoxicity in non-tumor cells. This observation aligns with previous studies that have linked high W residues content with increased hydrophobicity and enhanced membrane disruption in mammalian cells [67]. Overall, these results underscore the importance of balancing antimicrobial potency and host cell selectivity in peptide design [68].

Morphological analysis using SEM and TEM confirmed extensive damage to bacterial membranes after *E. coli* exposure to Ib-M peptides, including bulge formation, inner membrane retraction, cytoplasmic leakage, and cell lysis. Similar features were reported in *E. coli* treated with other antimicrobial peptides [24,69,70]. Notably, Ib-M peptides induced larger and more extensive bulges than PMB, suggesting a distinct mechanism of action [71,72]. Additional alterations, such as surface depressions, pore formation, and bacterial aggregation, further support membrane disruption as a primary antibacterial action [24,69,70]. The peptides seem to exert their bactericidal action by disrupting both the outer and inner membranes and affecting intracellular organization in similarly fashion as reported for Trp-rich AMPs [69]. Although bactericidal cytoplasmatic changes suggest the possibility of secondary intracellular targets, this remains speculative and warrants further investigation.

To understand the structure and function relationship involved in the Ib-M peptides antibacterial activity against *E. coli* ATCC 25922, our studies identified physicochemical properties, particularly cationicity and hydrophobicity as critical features directly associated with antibacterial activity against *E. coli* in corroboration with previous reports with other AMPs against *E. coli* [67,69,73,74]. It is well established that peptides with a net positive charge between +4 and +8 exhibit optimal cationicity for bactericidal activity [75,76]. Ib-M peptides displayed a net charge of +6, owing to the presence of seven R residues and a one E residue, placing them within this favorable range [75]. This balance of positive charge promotes electrostatic interactions with negatively charged bacterial membrane components, such as LPS. Supporting this, previous studies have shown that increasing the number of R residues in peptides such as Parasin R and WR12, enhances their membrane-permeabilizing and antibacterial effects [77,78]. Based on these reports and our current study, we hypothesize that the cationic nature of Ib-M peptides facilitates their interaction with LPS and the phospholipid bilayers of the bacterial membranes. The side chains of R residues, which contain guanidinium groups (C(NH_2_)_3_^+^), remain protonated even within the deepest membrane layers, enabling strong electrostatic interactions [79]. Moreover, R residues can form bidentate hydrogen bonds with lipid phosphate groups, promoting negative membrane curvature, thinning, and ultimately, membrane disruption [79,80].

These mechanistic insights were supported by microscopy analyses, which revealed substantial membrane deformation and lysis following treatment with Ib-M1, Ib-M2, and Ib-M6. Secondary structure prediction using PEP-FOLD3.5 revealed that W residues at positions 2 and 9 (N-terminal) and 18–20 (C-terminal) among Ib-M peptides. W residues can interact with both lipid headgroups and water molecules at the membrane interface via hydrogen bonding and, in fact, molecular simulations show that W residues are inserted into bacterial membranes. Our observations are consistent with those of previous studies on QL17 peptide analogs, where the presence of W in IL15.3 was found to be critical for membrane-disruptive activity [81]. Additionally, their planar indole rings support quadrupolar moments and cation-π interactions [82,83]. These interactions are further enhanced when W and R residues are distributed along the peptide sequence, promoting membrane insertion and subsequent bilayer disruption [80,84–86].

CD spectroscopy revealed that Ib-M peptides transitioned from disordered states in aqueous environments to α-helical conformations under membrane-mimicking conditions. Ib-M peptides adopted a higher α-helical content in SDS ranging from 40.3% (Ib-M2) to 73.9% (Ib-M1) than in TFE (4.2–15.6%), which agrees with previous studies on cationic and amphipathic peptides such as AR-23 and gp41w [87,88]. This behavior can be explained by the fact that SDS micelles provide an anionic interface that promotes the electrostatic attraction of positively charged residues, such as R, and facilitates W interfacial anchoring, thereby stabilizing a functional amphipathic helix [85,89]. These results are consistent with secondary structure predictions obtained through PEP-FOLD and support their membranolytic potential [61,87,90]. In contrast, TFE acts as a co-solvent that enhances intramolecular hydrogen bonding and promotes generalized helicity but lacks the ability to reproduce the specific lateral interactions of membranes, thus attenuating the amphipathic character of many antimicrobial peptides (AMPs) [91].

Additionally, helical wheel projections showed that polar residues interrupted the hydrophobic face of the peptides. This feature may contribute to the potential antibacterial activity observed in the three peptides and the low cytotoxicity of the Ib-M1 peptide. Ib-M peptides primarily consist of two short α-helices connected by a kink region (α–hinge–α motif) induced by P residues in Ib-M1 and Ib-M2. This structural arrangement is consistent with the AMP fowlicidin 2 analogs, specifically Fowl-2 (1–18 residues) and Fowl-2 (15–31 residues), which also feature two α-helical segments separated by a central kink and retain substantial antibacterial activity against *E. coli* [60]. Similar to these peptides, P residues in Ib-M1 and Ib-M2 contributes to conformational flexibility, potentially enhancing insertion into bacterial membranes [85,90]. A comparable structural characteristic was observed in Ib-M6, where the GWGRG motif is thought to facilitate its ability to interact with and disrupt bacterial membranes. Previous studies have shown that amidation can reduce structural rigidity by inducing polyproline II conformations, C-terminal amidation of Ib-M peptides may enhance and stabilize their secondary structure and may contribute to membrane penetration and overall antimicrobial activity [92].

The outer membrane is a distinctive structural feature of Gram-negative bacteria, and, among many functions, it may protect bacteria against antimicrobial peptides. It is composed of a phospholipid bilayer in the inner leaflet and LPS in the outer leaflet [93]. In this study, we demonstrated that Ib-M peptides competitively displaced the cadaverine probe in the BC displacement assay, indicating their affinity for the lipid A component of LPS [49]. The positively charged residues of Ib-M peptides likely interact with the bis-anionic di-glucosamine backbone of lipid A [49]. These results suggest that Ib-M peptides can bind to the same Mg^2+^-binding sites on LPS as PMB and other cationic peptides [59,94,95]. This interaction is known to destabilizes the LPS network, cause local membrane thinning and disrupt the bacterial outer membrane [22,30,96]. These findings support the hypothesis that Ib-M peptides in *E. coli* utilize a self-promoted uptake pathway. By disrupting the Mg^2+^ bridges between adjacent LPS molecules, Ib-M peptides facilitate their translocation across the outer membrane [97,98].

Previous studies have shown that the ability of peptides to displace divalent cations from *E. coli* LPS correlates with increased outer membrane permeability [97–99]. In line with these findings, mechanistic analyses confirmed that Ib-M1, Ib-M2, and Ib-M6 peptides induced a dose-dependent increase in NPN fluorescence, indicating rapid OM permeabilization in *E. coli*, as previously reported for other peptides [59,96,100]. Notably, this effect was most pronounced in bacteria treated with Ib-M2.

Once in the periplasmic space, Ib-M peptides can interact with the inner membrane (IM). The kinetics of IM permeability in *E. coli* following exposure to Ib-M peptides revealed a delay in membrane permeabilization after peptide exposure, similar to that observed in bacteria treated with cecropin P1 [101]. Alterations in IM permeability were detected between 60 minutes and eight hours post-exposure. These findings suggest that delayed signal development may reflect ongoing peptide-membrane interactions rather than immediate permeability [101]. A similar effect was reported for cecropin A, which exhibited a linear activity-dependent increase in IM permeabilization without reaching a plateau, consistent with the progressive membrane-disruptive action observed for Ib-M peptides [102]. For Ib-M2 and Ib-M6, IM permeability was only observed at 6.2 µM concentration. In contrast, Ib-M1 showed an inverted kinetic profile, with maximum permeability at 6.2 µM, followed by reduced permeability at 12.5 and 25 µM. This phenomenon may result from continuous peptide insertion facilitated by high OM permeability, leading to inner membrane saturation, increased membrane tension, and subsequent disruption and permeabilization of the membrane.

Bacteria maintain an electrochemical proton gradient across their cytoplasmic membrane, known as the proton motive force (PMF), which is essential for survival. PMF comprises two components: an electric potential (ΔΨ) and a transmembrane proton gradient (ΔpH) [103]. Our findings suggest that the PMF may be a target in the mechanism of action of Ib-M peptides against *E. coli* ATCC 25922. In this study, following outer membrane permeabilization, Ib-M peptides reached the cytoplasmic membrane, where they induced permeabilization and depolarization. The depolarization levels were comparable among the three peptides. At a MIC of 12.5 µM, a rapid increase in cytoplasmic membrane depolarization was observed, consistent with previous reports [100,104]. However, at higher concentrations, this tended to decline, particularly in cells treated with the Ib-M2 peptide, suggesting the involvement of compensatory membrane dynamics or concentration-dependent fortifying.

Based on these findings, we propose a model to explain the mechanism of action of the antimicrobial effect of Ib-M peptides on *E. coli.* We propose that at sublethal concentrations, Ib-M peptides engage in electrostatic interactions with LPS on the outer membrane via R residues [81,100]. These interactions are thought to induce α-helical conformational changes and enhance amphipathicity, enabling peptide penetration into the outer membrane [61,90]. At these concentrations, Ib-M peptides caused transient and reversible membrane disruption, which often resulted in delayed bacterial growth recovery. As peptide concentrations increase, they induce significant outer membrane permeabilization, bacterial outer membrane disruption and peptide entry into the inner membrane [96]. These actions lead to rapid depolarization and destabilization, ultimately resulting in irreversible membrane disruption and subsequent bacterial cell death [96].

## Conclusion

This study provides critical information on the structure and function of Ib-M peptides that give light into the mechanism of antimicrobial action at the molecular level. First, microdilution assays confirm the ability of Ib-M peptides of not only inhibiting *E. coli* and but also having bactericidal effect. Also, electron microscopy studies showed the severe alteration of bacterial morphology and specifically the disruption of bacterial membranes. Furthermore, studies on the peptides sequence composition and distribution of amino acids in Ib-M peptides- particularly those contributing to hydrophobicity, cationicity, and amphipathicity-were directly associated with their ability to interact with bacterial membranes and exert antibacterial effect. Rich in R and W, the Ib-M peptides exhibited secondary structures consisting of two α-helices separated by a central hinge region, which conferred conformational flexibility. Their bactericidal activity was strongly linked to interactions with, and neutralization of, bacterial LPS, as well as significant outer membrane disruption. In addition, Ib-M peptides permeabilized the inner membrane, disrupted membrane potential, and likely induced cell death by impairing the PMF.

Collectively, our findings reveal the possible mechanisms of action of Ib-M antibacterial peptides against *E. coli* ATCC 25922 and provide strong scientific evidence supporting their therapeutic potential for combating bacterial pathogens. Furthermore, this study offers new perspectives on the structure-function relationship of novel antimicrobial peptides. We anticipate that future research will expand on these findings to support the development of Ib-M peptides as promising candidates for treating infections caused by antimicrobial-resistant Gram-negative bacteria.

## Supporting information

S1 Table Characteristics of synthesis of Ib-M peptides. (PDF)

S2 Table Criteria for peptide models were obtained from the SWISS-MODEL server. The shaded models were selected based on their best scores.
(PDF)

S1 Fig Spectra of Spectrometry Mass of Ib-M peptides. **(A)** Ib-M1. **(B)** Ib-M2. **(C)** Ib-M6. MS measurements were carried out in positive reflectron mode with the following conditions: mass range between 150 and 2000 m/z; electrospray ionization (ESI) interface, nebulizing gas flow of 1.5 L/min; solvent and block temperature of 250° C and 200° C, respectively; interface bias +4.5 Kv secant gas flow, 5 L/min; and flow time, 0.2 mL/min. A 50% water/50% methanol (v/v) buffer solution was used. The injection volumes were 0.1 for Ib-M1 and Ib-M6, and 0.2 for Ib-M2.
(TIF)

S2 Fig Spectra of HPLC of Ib-M peptides. **(A)** Ib-M1. **(B)** Ib-M2. **(C)** Ib-M6. M1, M2, and M6 correspond to each one of the analyzed peptides. Retention time (tR) was determined by analytical HPLC using buffer A (0.065% trifluoroacetic acid (TFA) in 100% water (v/v)), and elution was performed at a gradient of 5–95% in buffer B (0.05% TFA in 100% v/v acetonitrile) for 35 min at a flow rate of 1.0 mL/min. The total flow rate was 1 mL/min, and the measurements were performed at 220 nm.
(TIF)

S3 Fig Scanning electronic microscopy of *E. coli* treated with Ib-M peptides. **(30 and 120 minutes). (A, B)** *E. coli* without treatment. **(C, D)** Ib-M1. **(E, F)** Ib-M2. **(G, H)** Ib-M6. **(I, J)** PMB. The alterations are indicated as follows: large bulges or spherical elements (asterisk), bulges in formation (white arrowhead), collapsed cell (white circle), pores (white arrow), wrinkles (yellow arrow), deep invaginations (white squares) and unchanged surface cells (black squares). All images are representative of three biological replicates with similar results.
(JPG)

S4 Fig Chemical structure of Ib-M peptides. **(A)** Ib-M1. **(B)** Ib-M2. **(C)** Ib-M6.
(JPG)

S5 Fig *E. coli* LPS displacement. (JPG)

S6 Fig Outer membrane permeability kinetics. **(A)** Ib-M1. **(B)** Ib-M2. **(C)** Ib-M6. **(D)** PMB. **(E)** Streptomycin.
(JPG)

S1 Raw Images This corresponding to uncropped images of Fig 2. (ZIP)

S2 Raw Images This corresponding to uncropped images of S3 Fig. (ZIP)

S3 Raw Images This corresponding to uncropped images of Fig 3. (ZIP)

S1 File Raw data for Tables 1, 2, and 3, and Figs 1 and 4. (XLSX)

S2 File Raw data for Table 6. (XLSX)

S3 File Raw data for Fig 8, S5 Fig, and S6 Fig. (XLSX)

S4 File Raw data for Fig 9. (XLSX)
