# Supplementary material for: Structural and functional analysis of Escherichia coli membrane disruption by Ib-M peptides
Source: PLoS One. 2025 Oct 8;20(10):e0334029. doi: 10.1371/journal.pone.0334029 (PMC12507217; doi:10.1371/journal.pone.0334029)
Supplement: S1 Table — (PDF) [file pone.0334029.s001.pdf]

Peptide	Amino acid Sequence	Number amino acids	Net charge	Theoretical MW[a] (Da)	Observed MW[a] (Da)	% HPLC purity	HPLC t_R[b] (min)
**Ib-M1**	EWGRRMMGRGPGRRMMRWWR	20	+6	2648.19	2649.0	≥98.1	10.906
**Ib-M2**	EWGRRMMGWRPGRRMMRWWR	20	+6	2777.35	2777.5	99.4	10.692
**Ib-M6**	EWGRRMMGWGRGRRMMRRWW	20	+6	2737.29	2737.8	98.0	14.436

Ib-M1, Ib-M2, and Ib-M6 (23) were designed based on the sequence information of the native Ib-AMP4 (24). [a]MW. Molecular Weight. Da. Daltons. Glutamic acid (E), Tryptophan (W), Glycine (G), Arginine (R), Methionine (M), Proline (P).
