# Supplementary material for: Structural and functional analysis of Escherichia coli membrane disruption by Ib-M peptides
Source: PLoS One. 2025 Oct 8;20(10):e0334029. doi: 10.1371/journal.pone.0334029 (PMC12507217; doi:10.1371/journal.pone.0334029)
Supplement: S2 Table — The shaded models were selected based on their best scores. (PDF) [file pone.0334029.s002.pdf]

**Ib-M1 models**	**MolProbity Score**	**Clash Score**	**Ramachandran Favoured %**	**Ramachandran Outliers**	**Rotamer Outliers %**	**C-Beta Deviations**	**Bad Bonds**	**Bad Angles**	**Cis/Twisted Prolines/Non-Prolines**
1	1.20	0	82.35	5.88 (11PRO)	0	1 (1GLU)	0/187	0/244	
2	1.09	0	88.24	5.88 (3GLY)	0	1(20ARG)	0/187	0/244	
3	1.65	2.72	88.24	0	0	1 (1GLU)	0/187	1/244	
4	1.28	0	76.47	0	0	0	0/187	1/244	
5	1.06	2.72	100	0	0	0	0/187	1/244	
**Ib-M2 models**	**MolProbity Score**	**Clash Score**	**Ramachandran Favoured %**	**Ramachandran Outliers**	**Rotamer Outliers %**	**C-Beta Deviations**	**Bad Bonds**	**Bad Angles**	**Cis/Twisted Prolines/Non-Prolines**
1	1.64	2.60	88.24	5.88 (2TRP)	0	0	1/199	0/262	
2	1.87	5.19	88.24	0	0	0	0/199	0/262	
3	1.44	2.60	94.12	0	0	2(20ARG, 1GLU)	0/199	1/262(20 ARG)	
4	1.82	2.60	76.47	11.76% (9TRP, 12 GLY)	0	1(1GLU)	1/ 199(9TRP)	1/262(1 GLU)	
5	1.28	0	76.47	11.76% (11 PRO, 12GLY)	0	1 (20 ARG)	1/199(2 TRP)	2/ 262(20ARG)	
**Ib-M6 models**	**MolProbity Score**	**Clash Score**	**Ramachandran Favoured %**	**Ramachandran Outliers**	**Rotamer Outliers %**	**C-Beta Deviations**	**Bad Bonds**	**Bad Angles**	**Cis/Twisted Prolines/Non-Prolines**
1	2.54	2.65	64.71	0	6.25% A15 MET	0	0/195	1/255(1GLU)	1/19(10 GLY,11 ARG)
2	2.01	2.65	52.94	17.65% (10 GLY, 9TRP, 2 TRP)	0	0	1/195 (9TRP)	0/255	
3	2.58	2.65 (9 TRP,10GLY)	58.82	17.65% (2 TRP, 9TRP, 12GLY)	6.25% A20 TRP	0	1/195(19 TRP)	1/255(20TRP)	
4	1.65	2.65	88.24	0	0	2 (1GLU, 13ARG)	0/195	1/255(1GLU)	
5	1.20	0	82.35	11.76% (10 GLY, 9TRP)	0	0	0/195	2/255(9TRP,8 GLY,9TRP)	
