# Supplementary figures and images for: Structural and functional analysis of Escherichia coli membrane disruption by Ib-M peptides
Source: PLoS One. 2025 Oct 8;20(10):e0334029. doi: 10.1371/journal.pone.0334029 (PMC12507217; doi:10.1371/journal.pone.0334029)

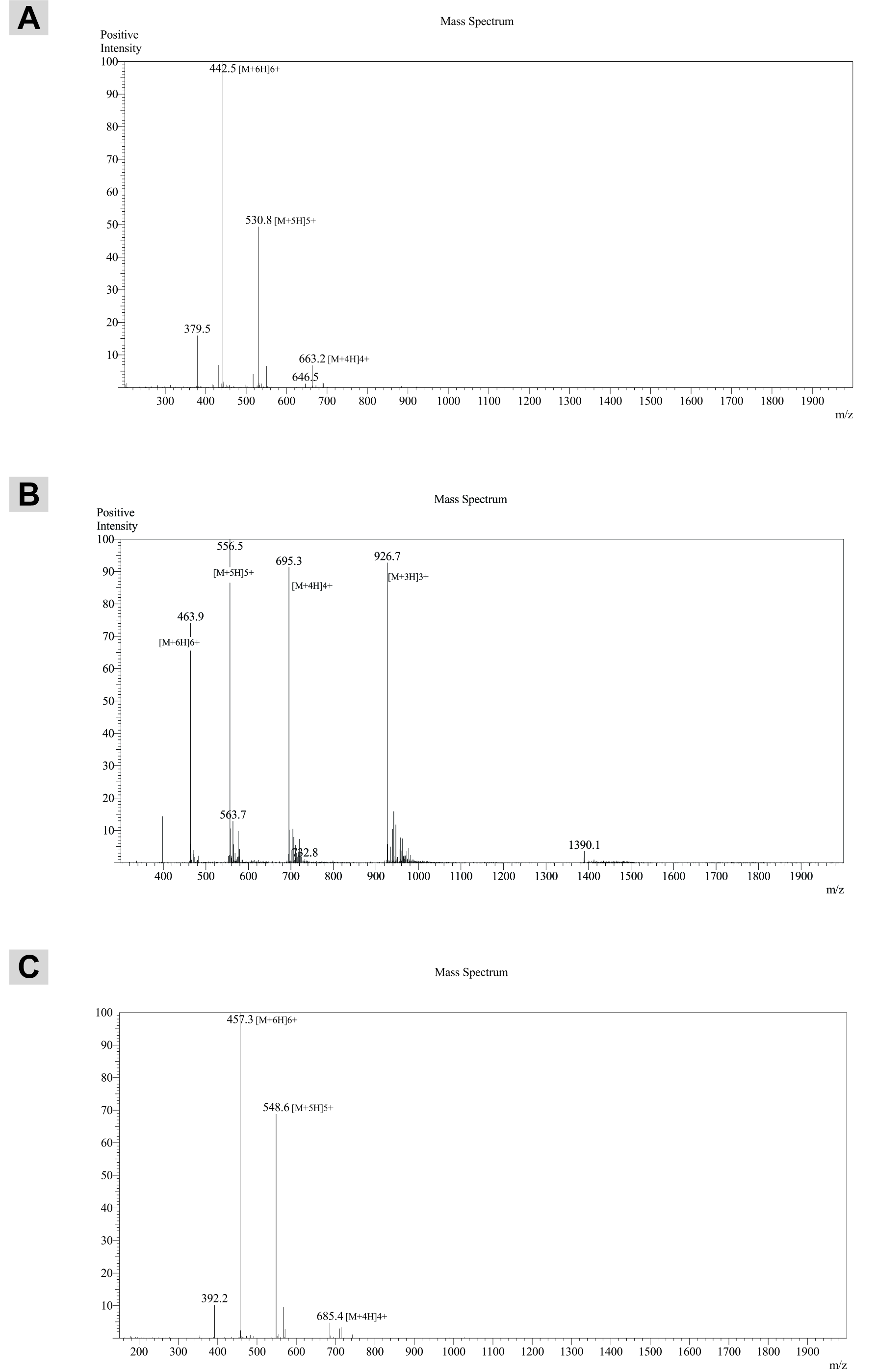

Supplement: S1 Fig — (A) Ib-M1. (B) Ib-M2. (C) Ib-M6. MS measurements were carried out in positive reflectron mode with the following conditions: mass range between 150 and 2000 m/z; electrospray ionization (ESI) interface, nebulizing gas flow of 1.5 L/min; solvent and block temperature of 250° C and 200° C, respectively; interface bias +4.5 Kv secant gas flow, 5 L/min; and flow time, 0.2 mL/min. A 50% water/50% methanol (v/v) buffer solution was used. The injection volumes were 0.1 for Ib-M1 and Ib-M6, and 0.2 for Ib-M2. (TIF) [file pone.0334029.s003.tif]

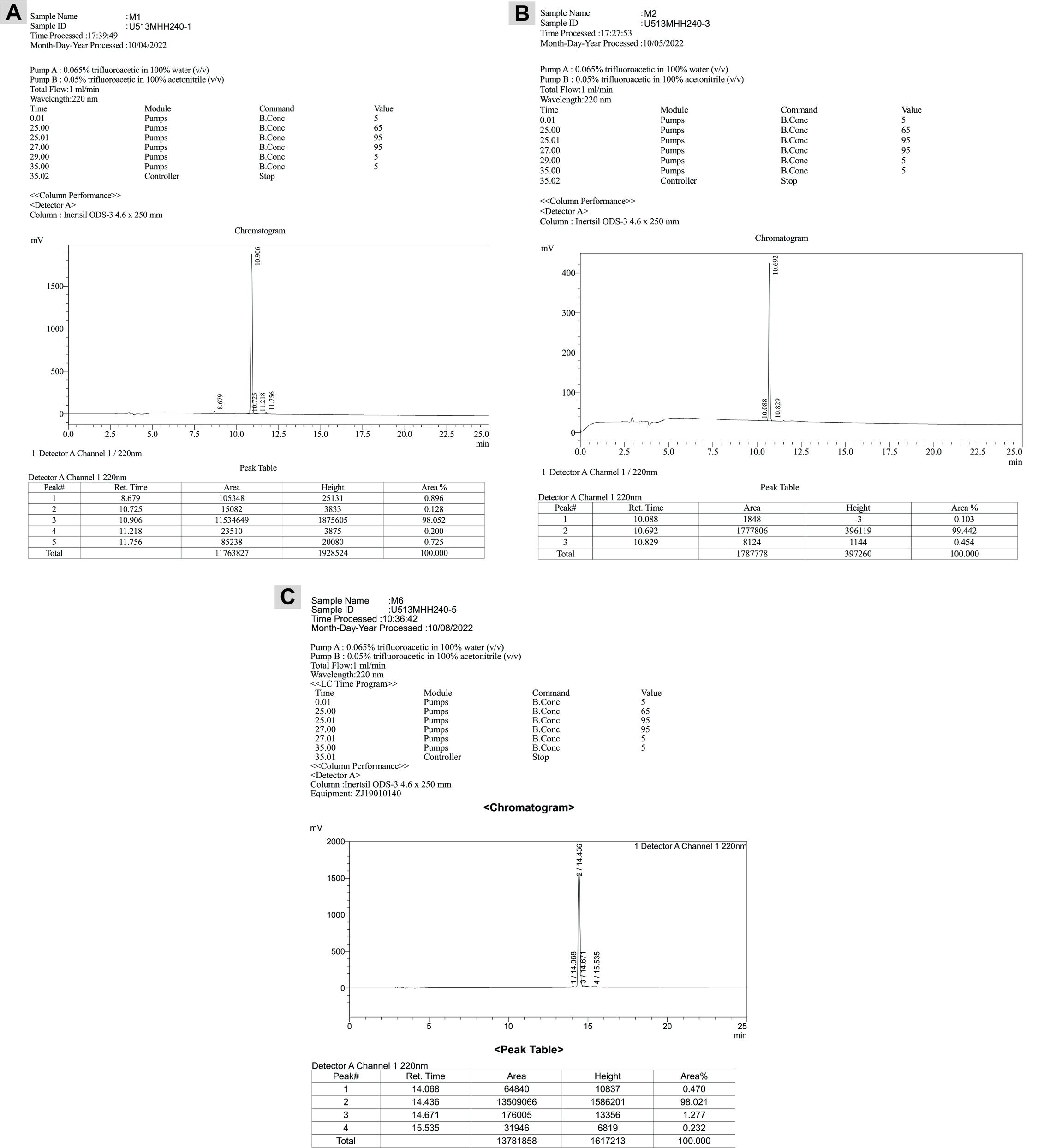

Supplement: S2 Fig — (A) Ib-M1. (B) Ib-M2. (C) Ib-M6. M1, M2, and M6 correspond to each one of the analyzed peptides. Retention time (tR) was determined by analytical HPLC using buffer A (0.065% trifluoroacetic acid (TFA) in 100% water (v/v)), and elution was performed at a gradient of 5–95% in buffer B (0.05% TFA in 100% v/v acetonitrile) for 35 min at a flow rate of 1.0 mL/min. The total flow rate was 1 mL/min, and the measurements were performed at 220 nm. (TIF) [file pone.0334029.s004.tif]

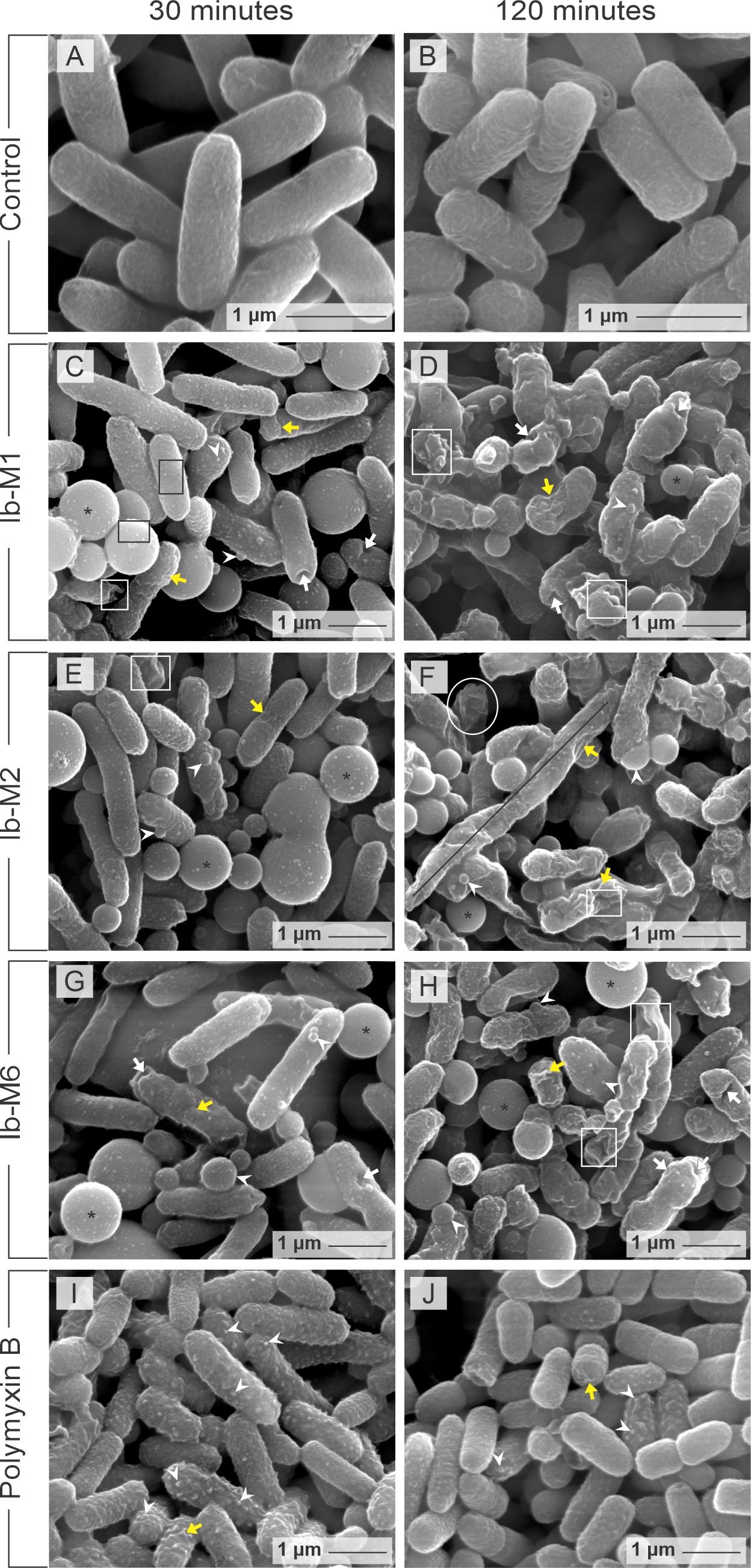

Supplement: S3 Fig — (30 and 120 minutes). (A, B) E. coli without treatment. (C, D) Ib-M1. (E, F) Ib-M2. (G, H) Ib-M6. (I, J) PMB. The alterations are indicated as follows: large bulges or spherical elements (asterisk), bulges in formation (white arrowhead), collapsed cell (white circle), pores (white arrow), wrinkles (yellow arrow), deep invaginations (white squares) and unchanged surface cells (black squares). All images are representative of three biological replicates with similar results. (JPG) [file pone.0334029.s005.jpg]

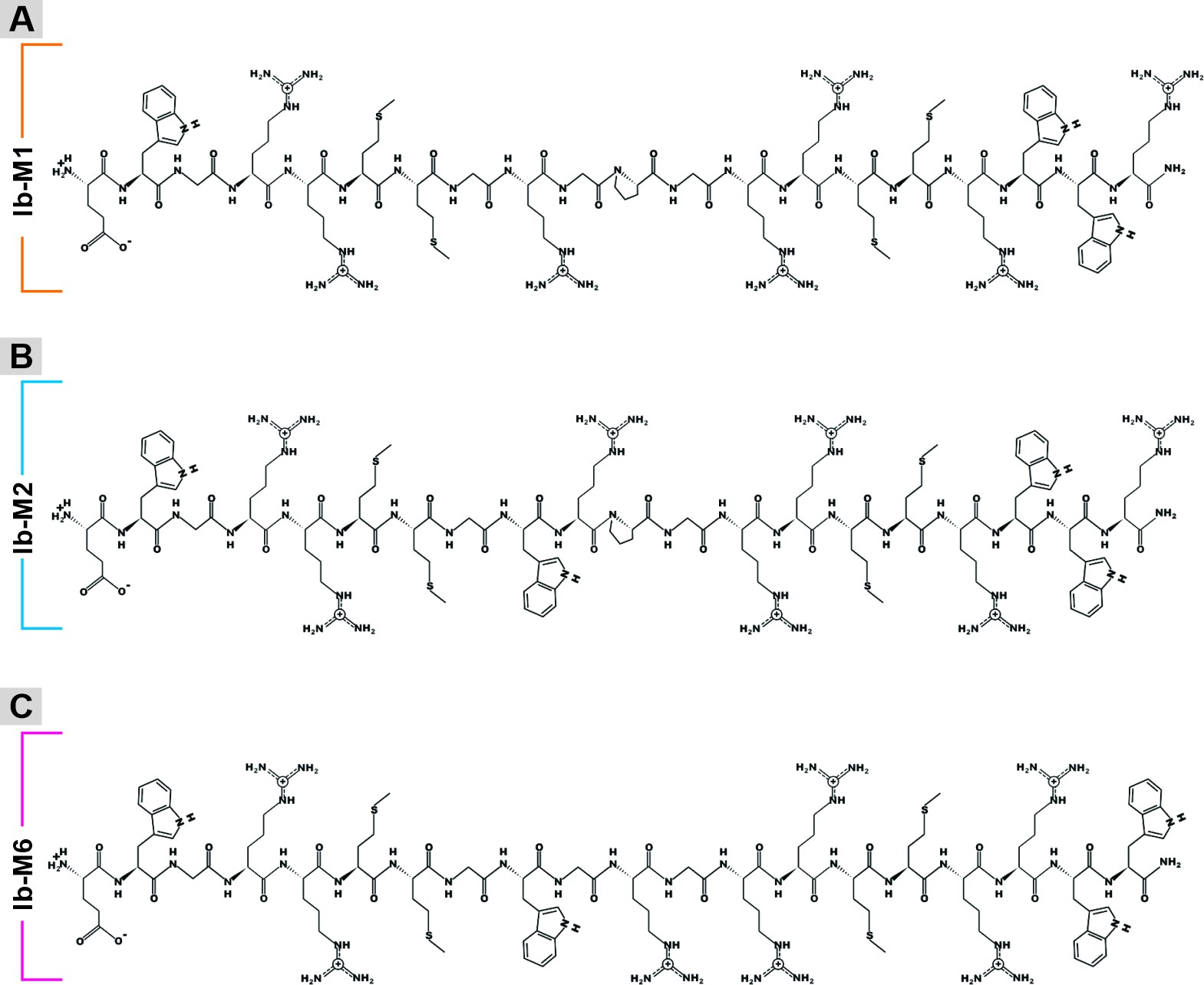

Supplement: S4 Fig — (A) Ib-M1. (B) Ib-M2. (C) Ib-M6. (JPG) [file pone.0334029.s006.jpg]

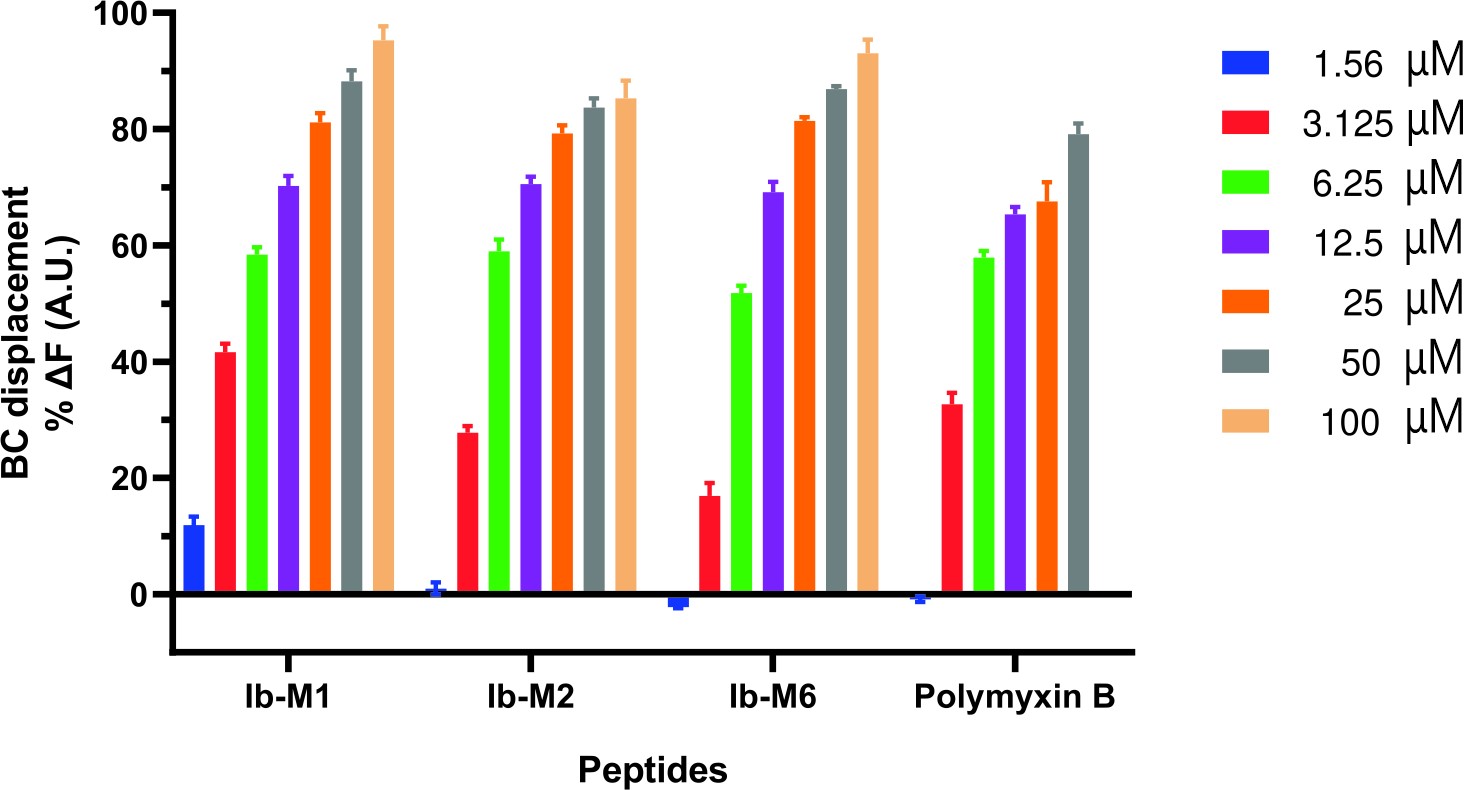

Supplement: S5 Fig — (JPG) [file pone.0334029.s007.jpg]

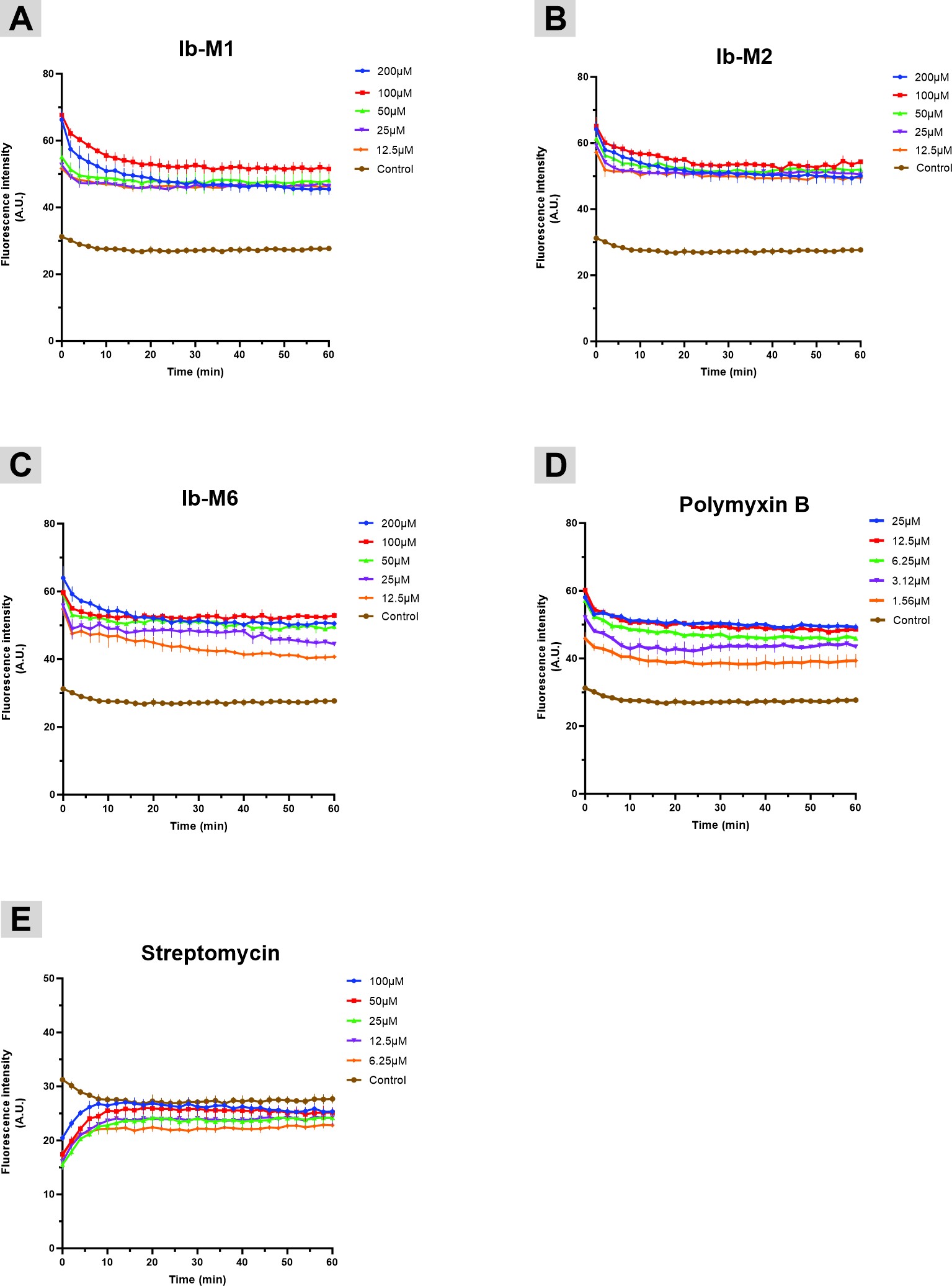

Supplement: S6 Fig — (A) Ib-M1. (B) Ib-M2. (C) Ib-M6. (D) PMB. (E) Streptomycin. (JPG) [file pone.0334029.s008.jpg]

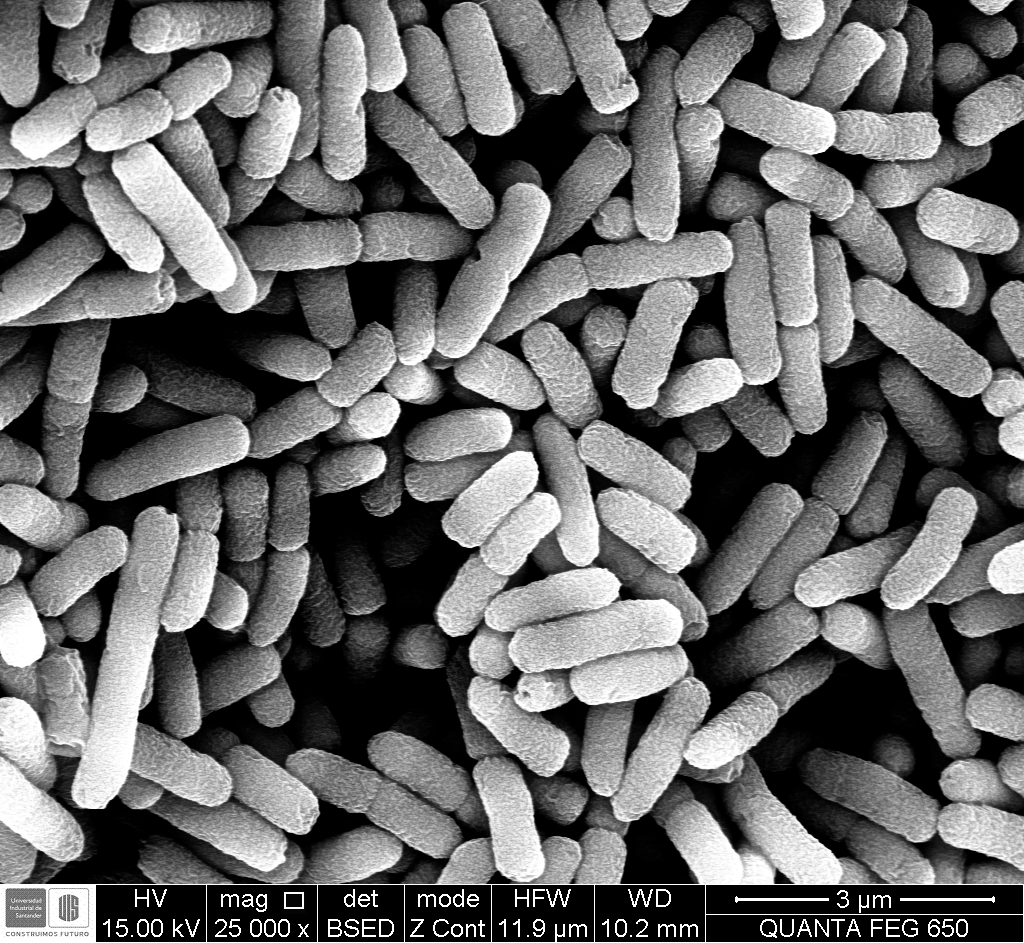

Supplement: S1 Raw Images — (ZIP) [file pone.0334029.s009.zip › S4Fig/2A_25X.tif]

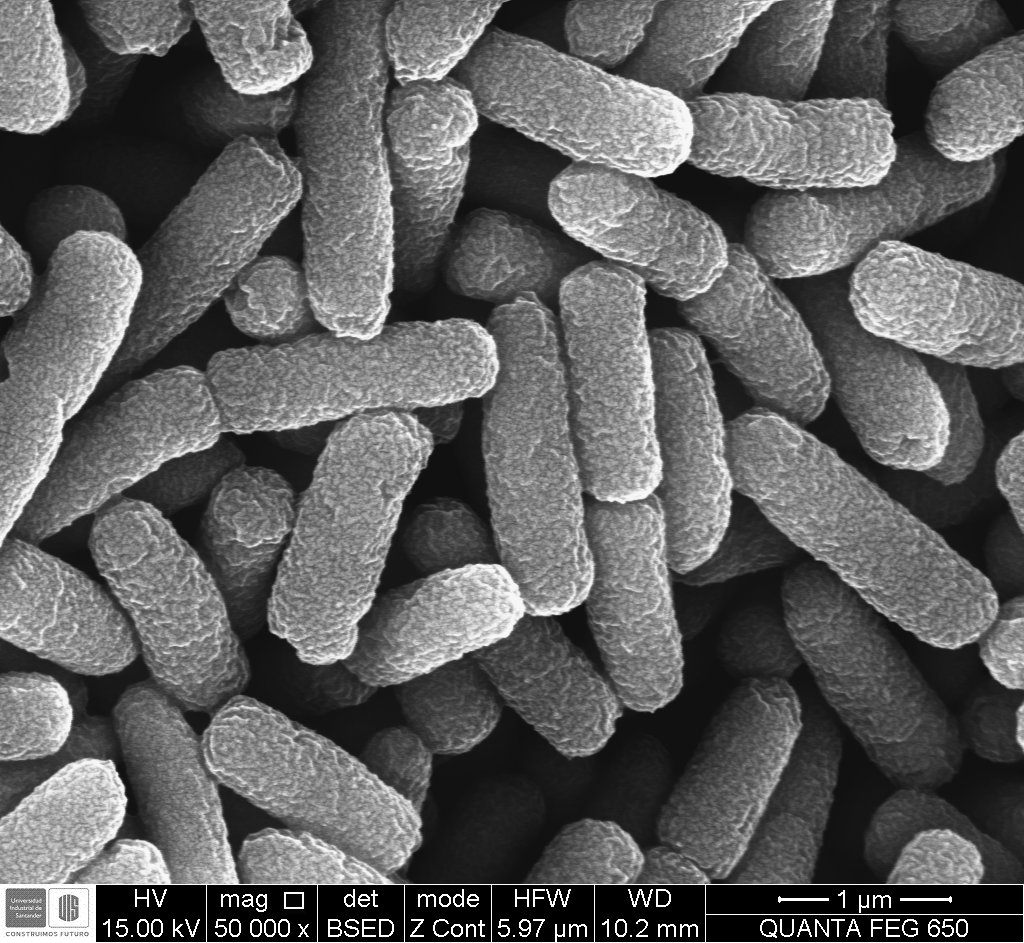

Supplement: S1 Raw Images — (ZIP) [file pone.0334029.s009.zip › S4Fig/2B_50X.tif]

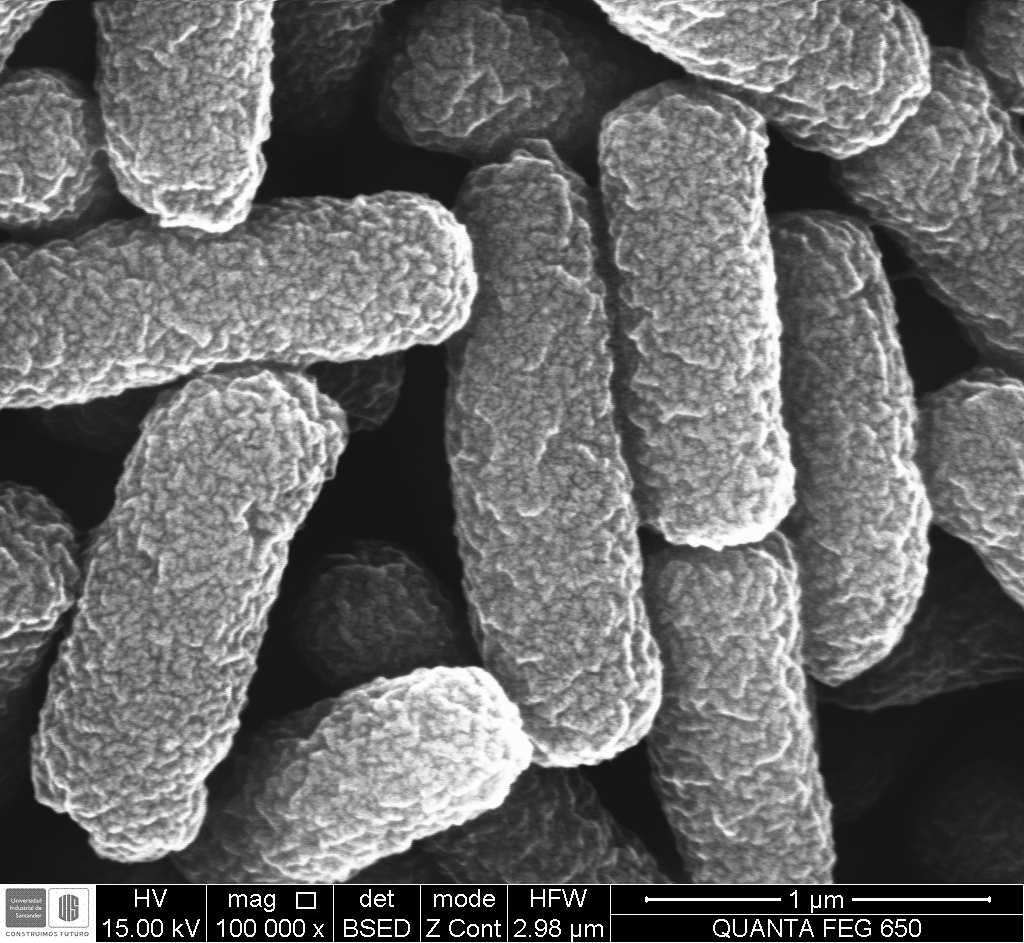

Supplement: S1 Raw Images — (ZIP) [file pone.0334029.s009.zip › S4Fig/2C_100X.tif]

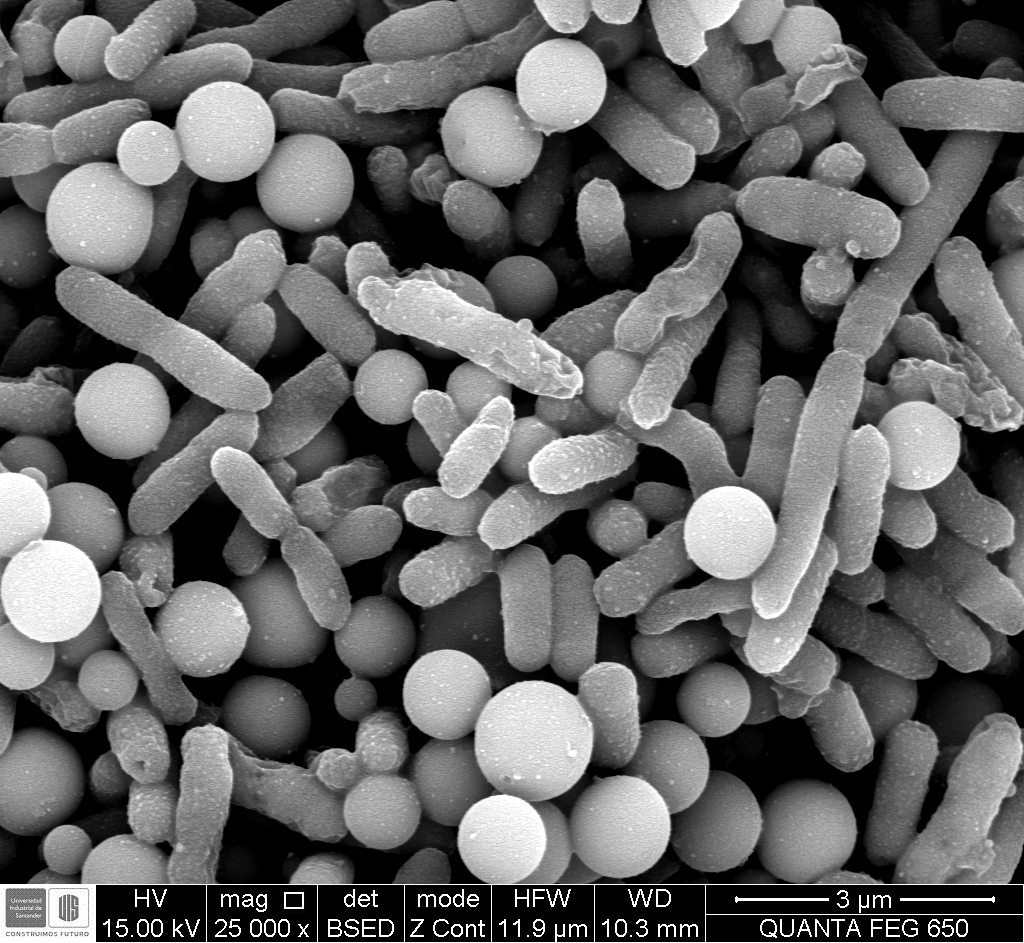

Supplement: S1 Raw Images — (ZIP) [file pone.0334029.s009.zip › S4Fig/2D_25X.tif]

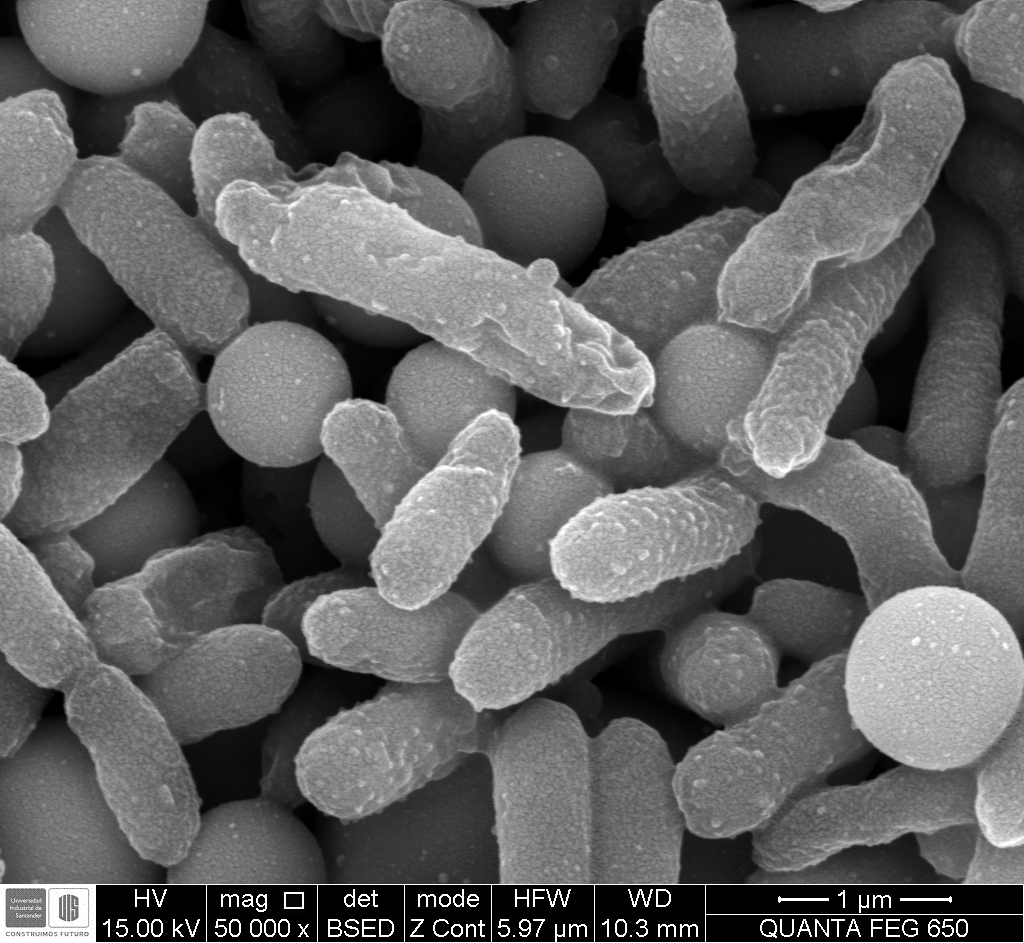

Supplement: S1 Raw Images — (ZIP) [file pone.0334029.s009.zip › S4Fig/2E_50X.tif]

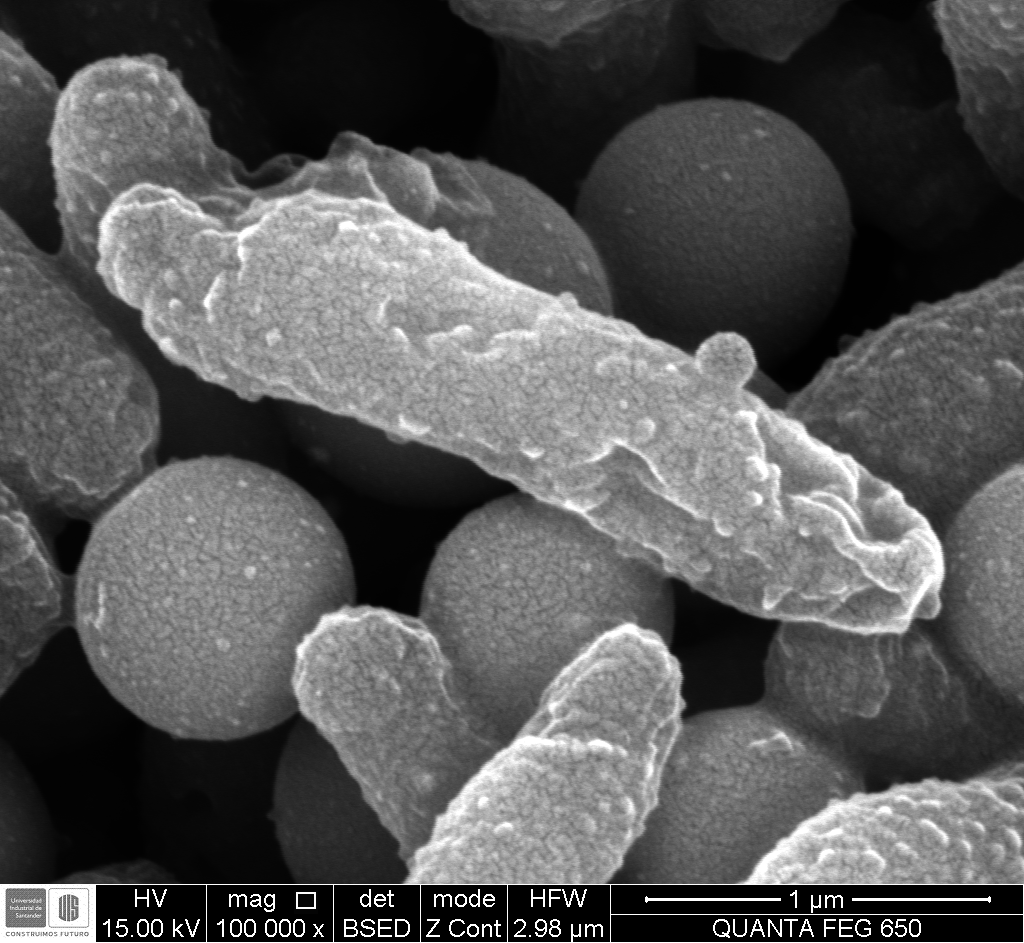

Supplement: S1 Raw Images — (ZIP) [file pone.0334029.s009.zip › S4Fig/2F_100X.tif]

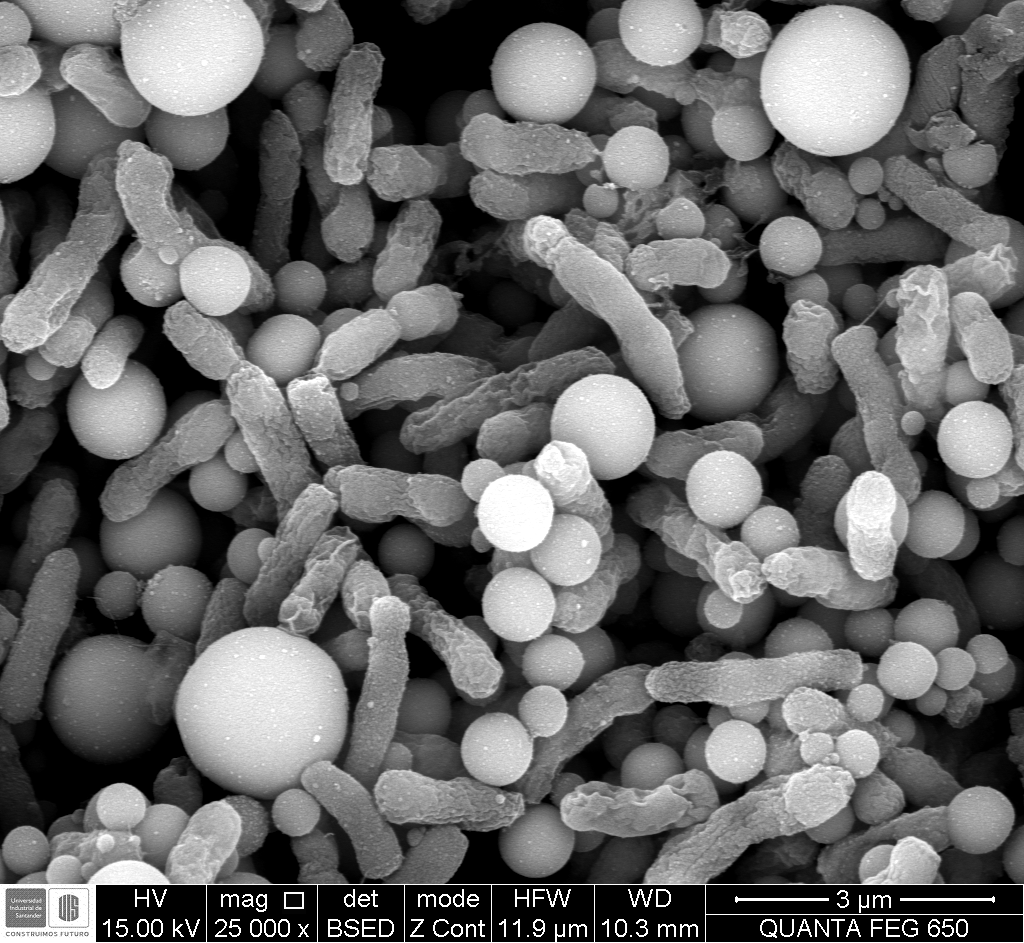

Supplement: S1 Raw Images — (ZIP) [file pone.0334029.s009.zip › S4Fig/2G_25X.tif]

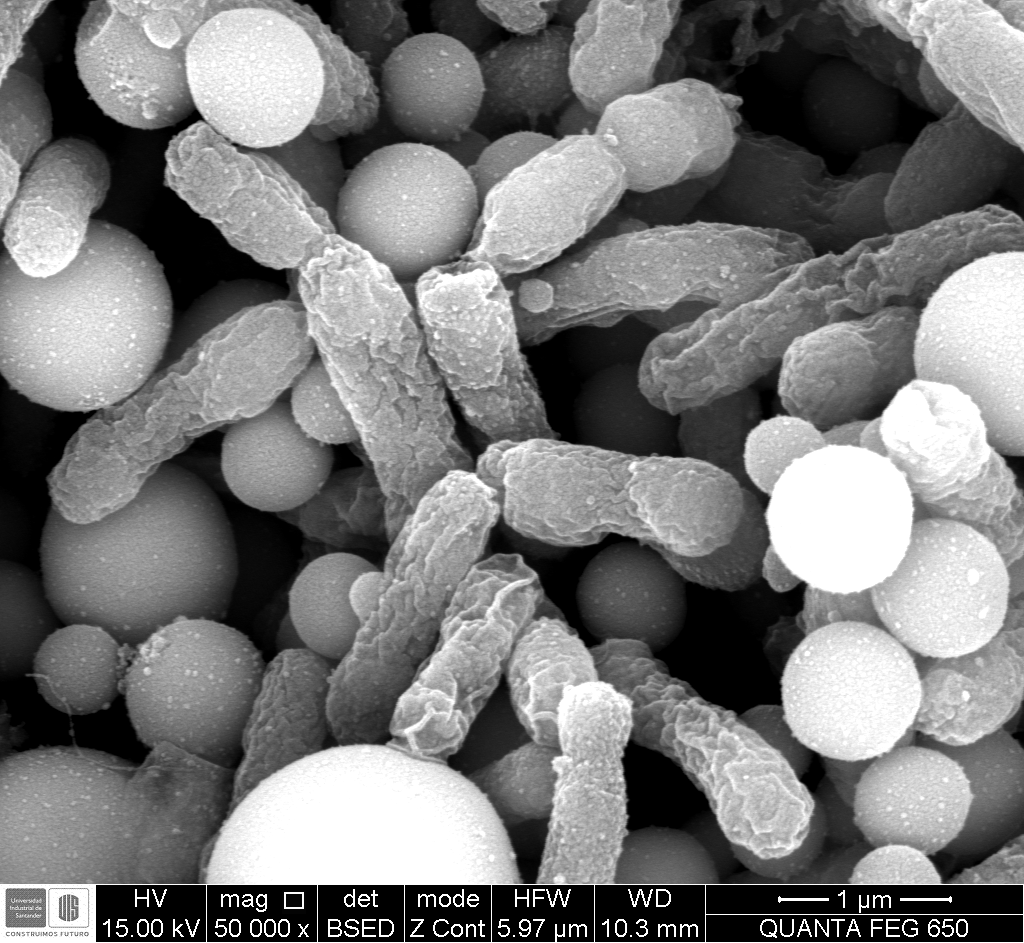

Supplement: S1 Raw Images — (ZIP) [file pone.0334029.s009.zip › S4Fig/2H_50X.tif]

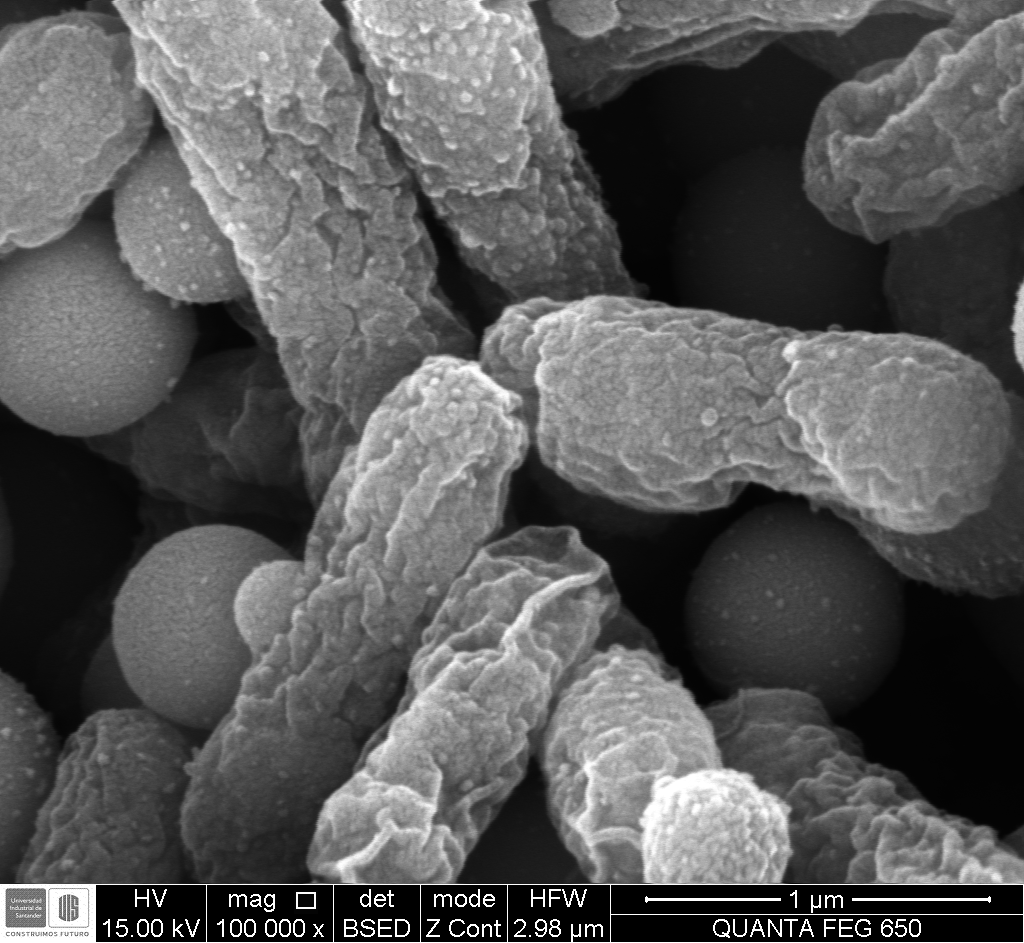

Supplement: S1 Raw Images — (ZIP) [file pone.0334029.s009.zip › S4Fig/2I_100X.tif]

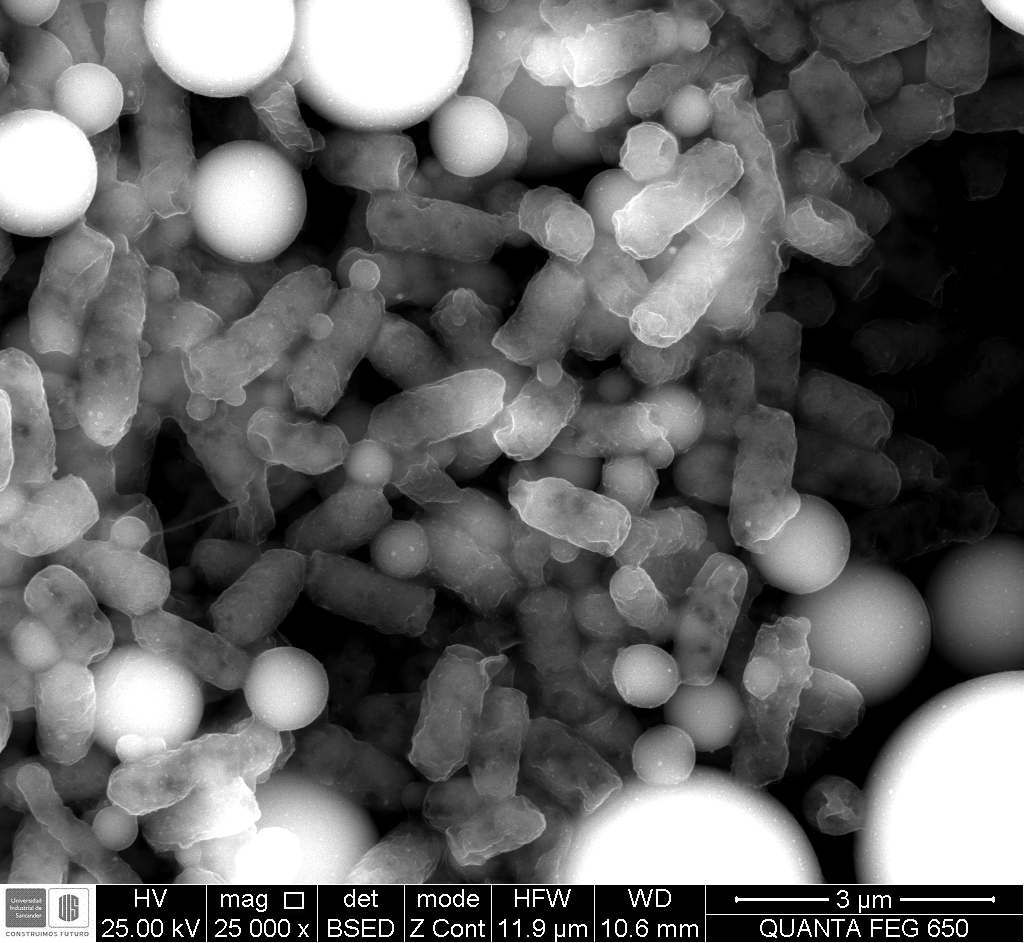

Supplement: S1 Raw Images — (ZIP) [file pone.0334029.s009.zip › S4Fig/2J_25X.tif]

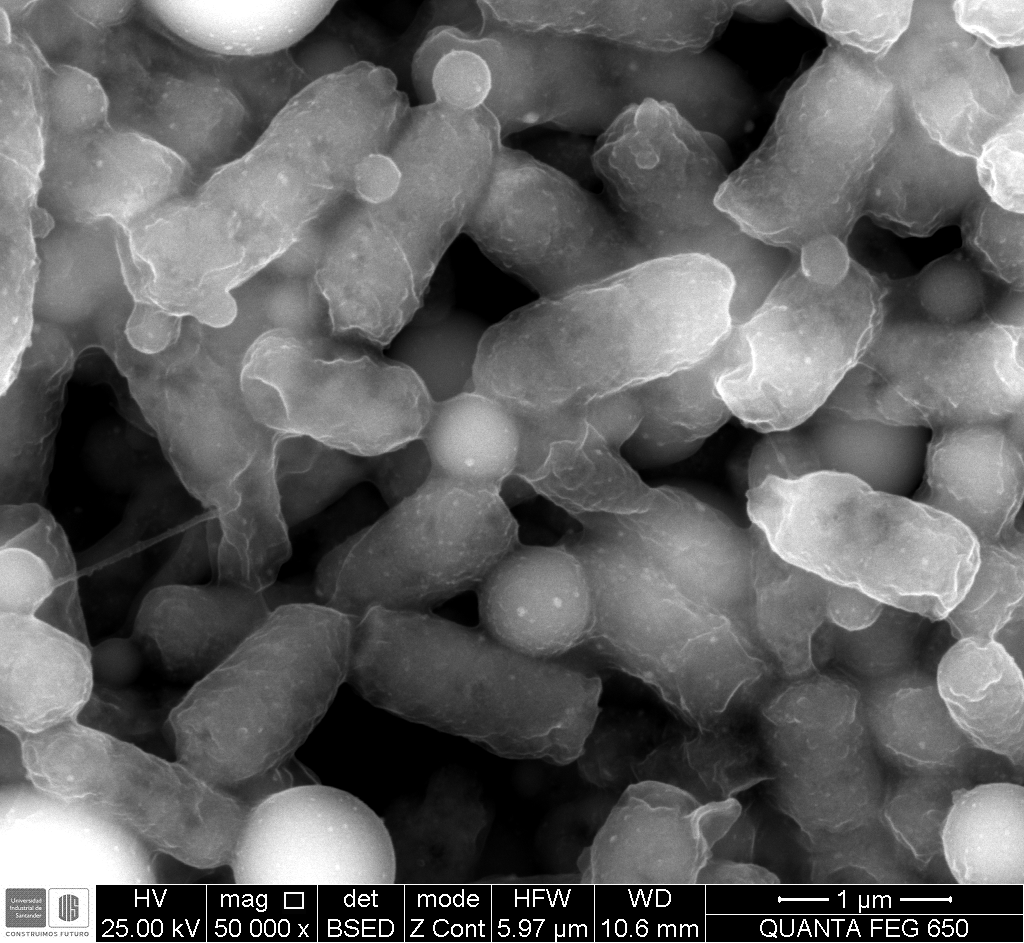

Supplement: S1 Raw Images — (ZIP) [file pone.0334029.s009.zip › S4Fig/2K_50X.tif]

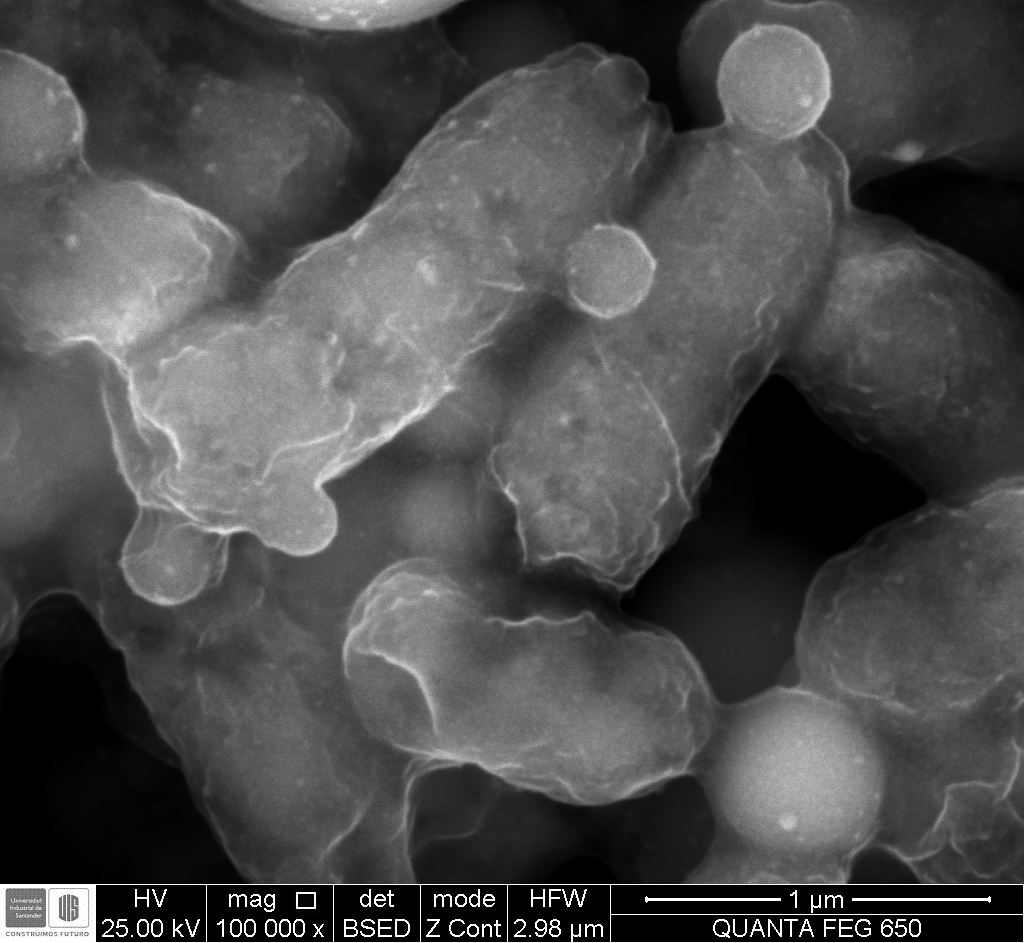

Supplement: S1 Raw Images — (ZIP) [file pone.0334029.s009.zip › S4Fig/2L_100X.tif]

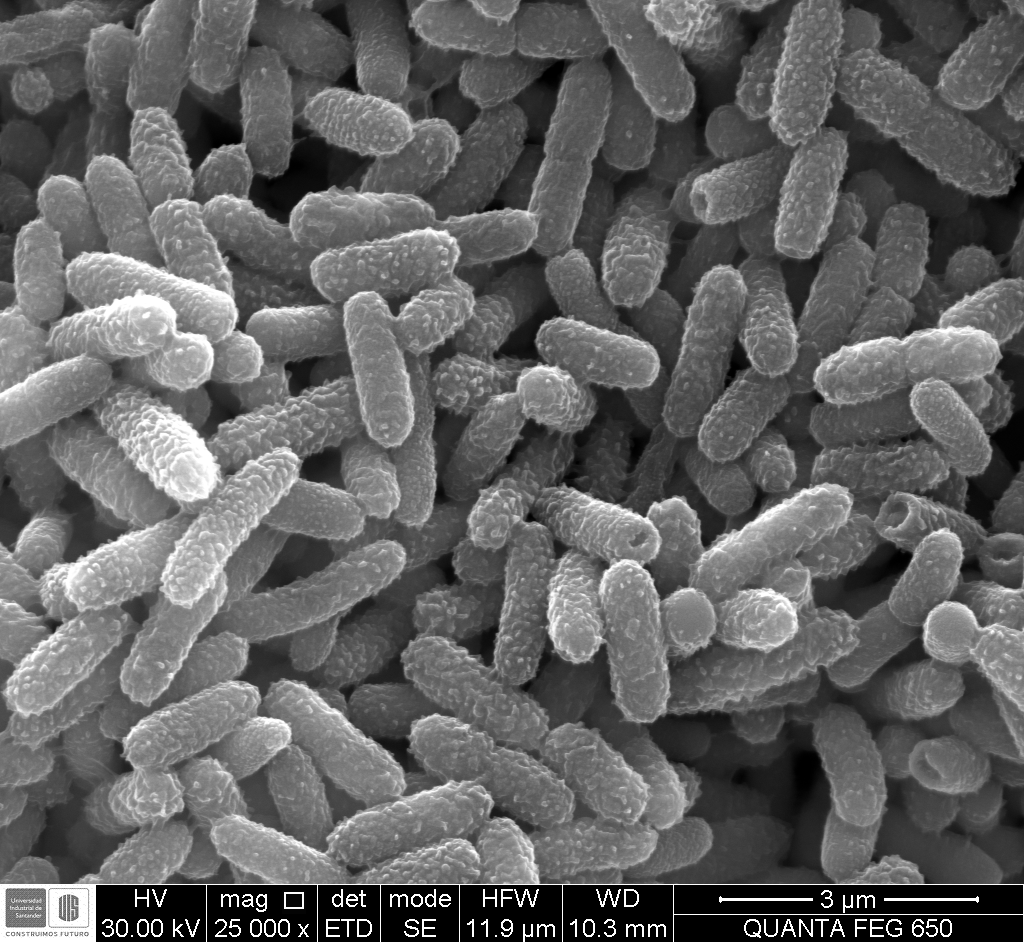

Supplement: S1 Raw Images — (ZIP) [file pone.0334029.s009.zip › S4Fig/2M_25X.tif]

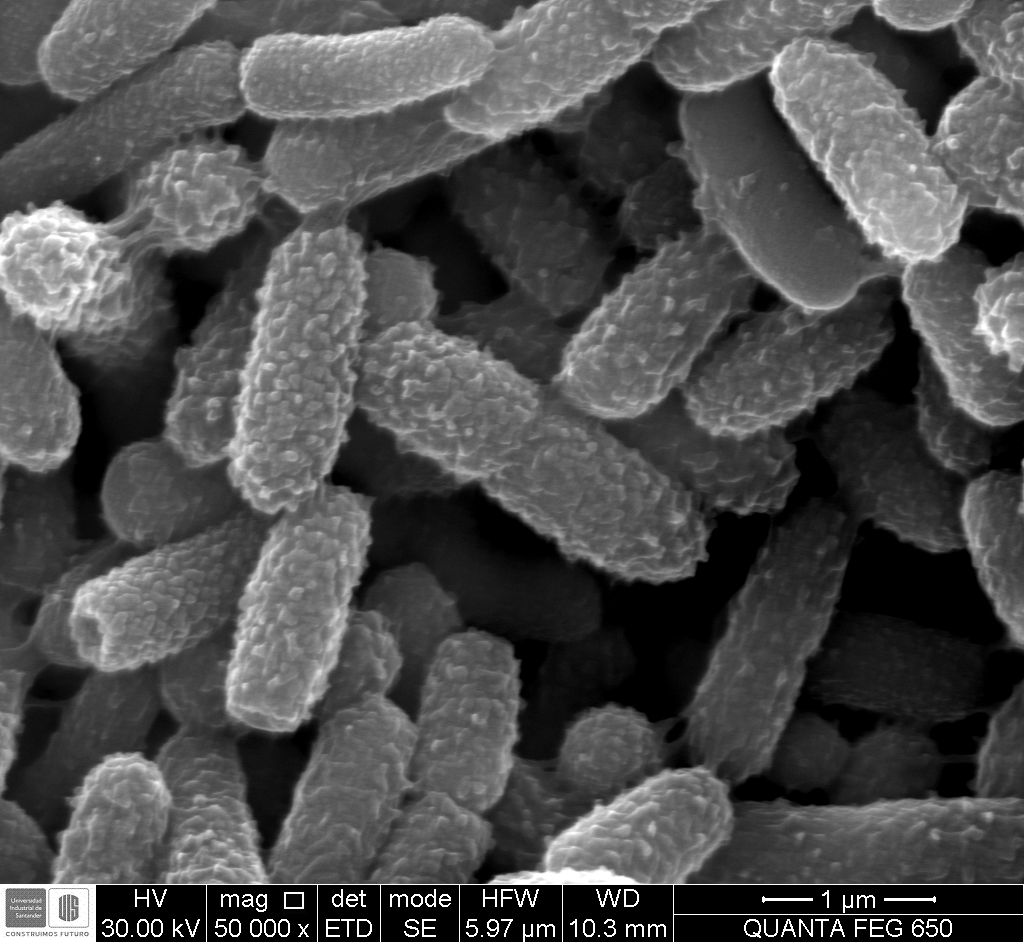

Supplement: S1 Raw Images — (ZIP) [file pone.0334029.s009.zip › S4Fig/2N_50X.tif]

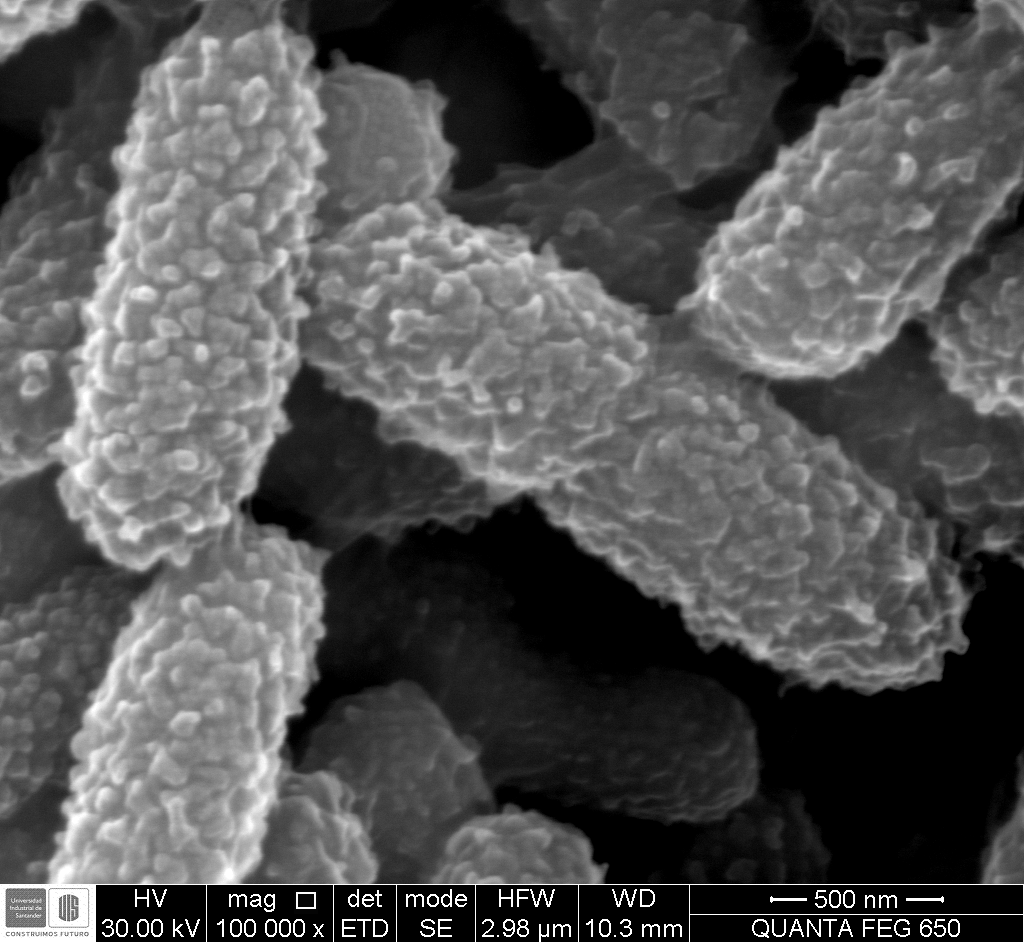

Supplement: S1 Raw Images — (ZIP) [file pone.0334029.s009.zip › S4Fig/2O_100X.tif]

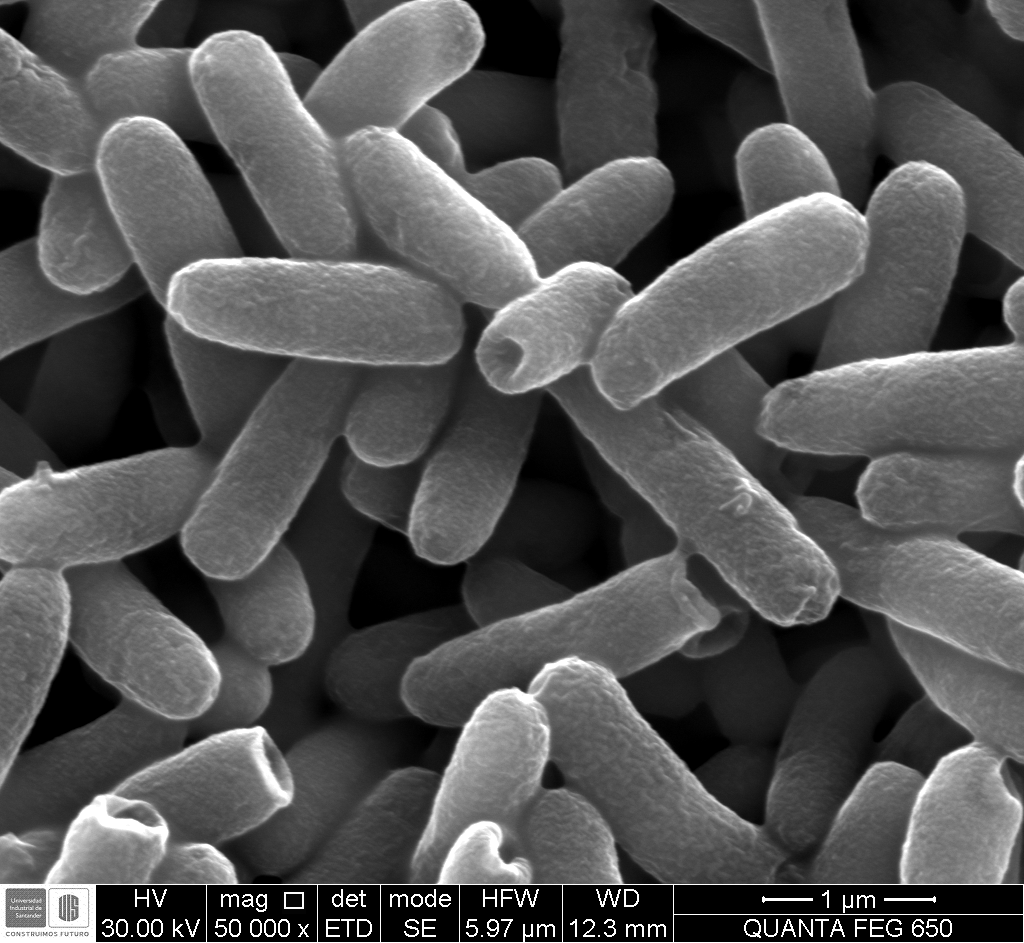

Supplement: S2 Raw Images — (ZIP) [file pone.0334029.s010.zip › S5Fig/S3A_50X.tif]

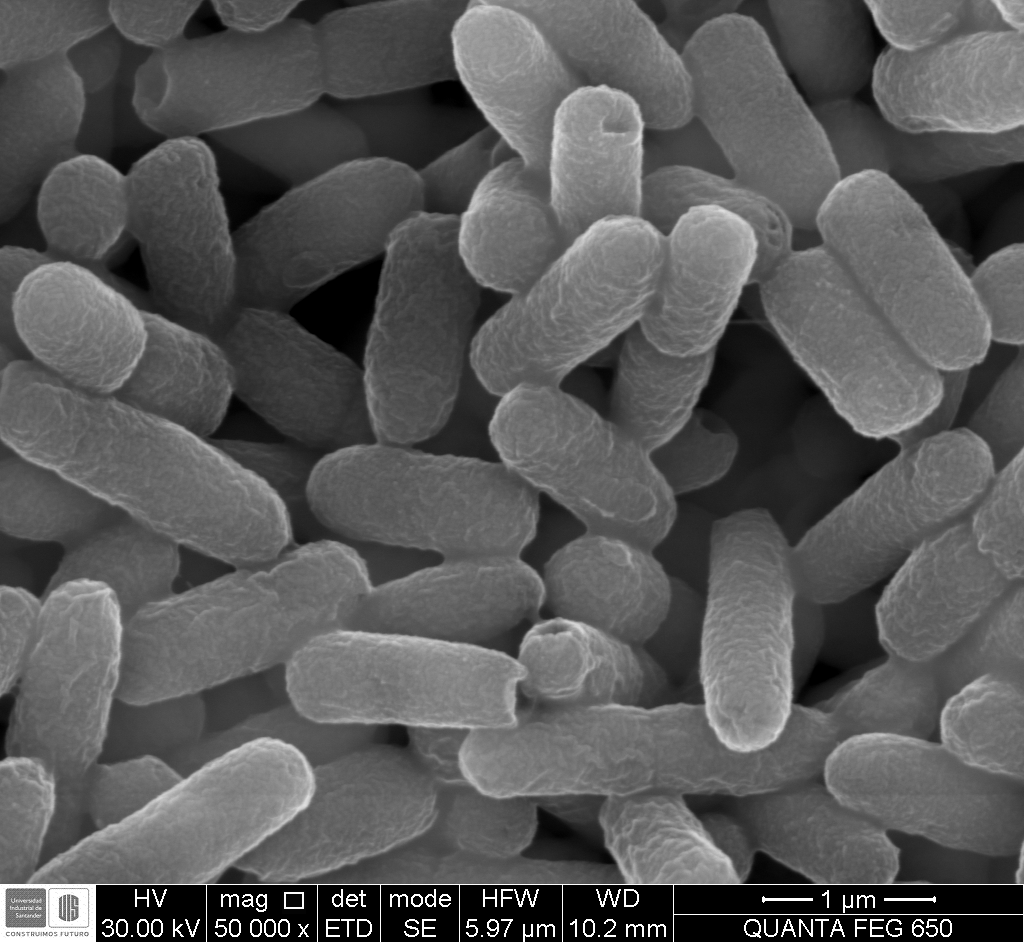

Supplement: S2 Raw Images — (ZIP) [file pone.0334029.s010.zip › S5Fig/S3B_50X.tif]

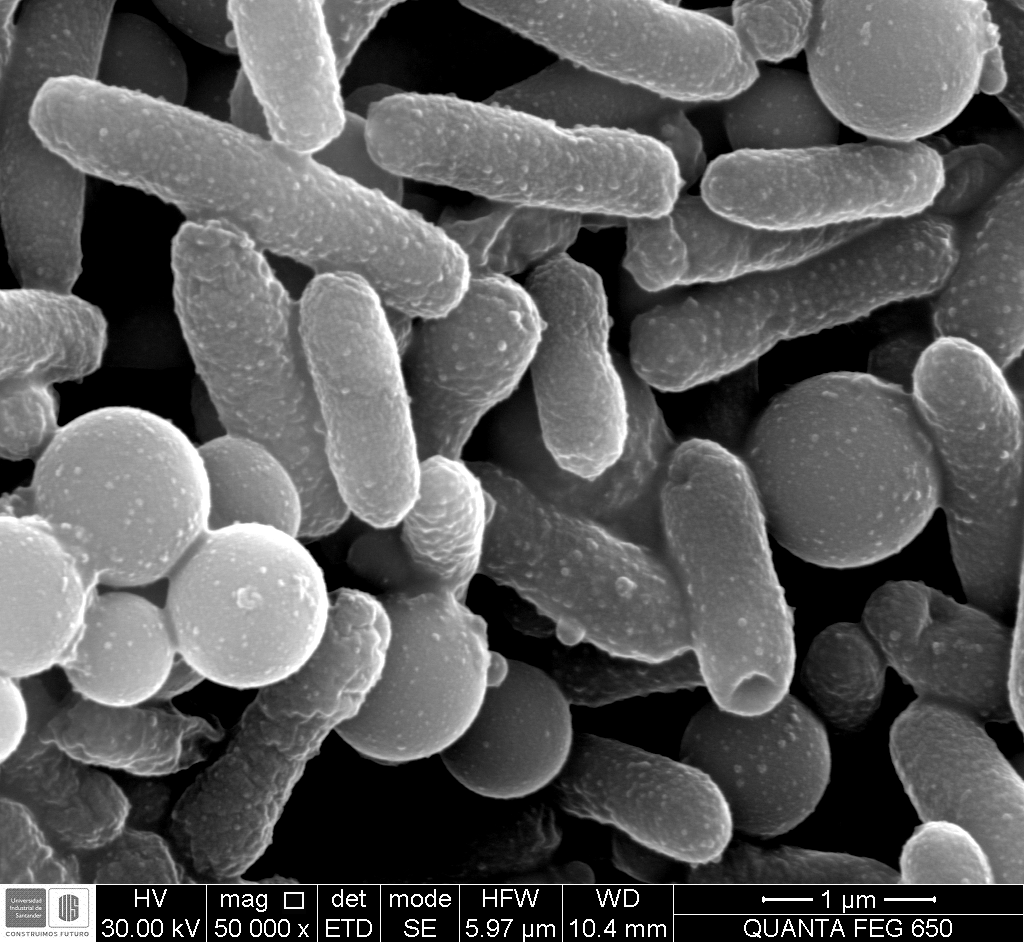

Supplement: S2 Raw Images — (ZIP) [file pone.0334029.s010.zip › S5Fig/S3C_50X.tif]

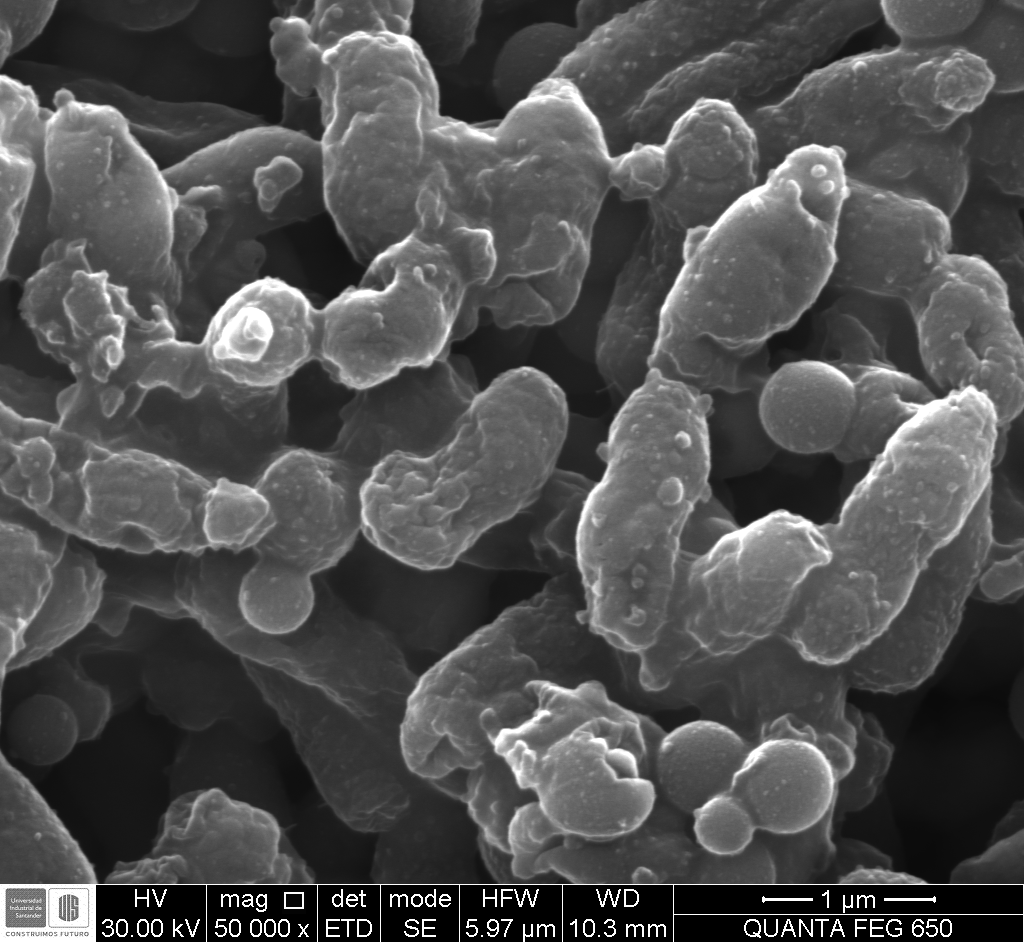

Supplement: S2 Raw Images — (ZIP) [file pone.0334029.s010.zip › S5Fig/S3D_50X.tif]

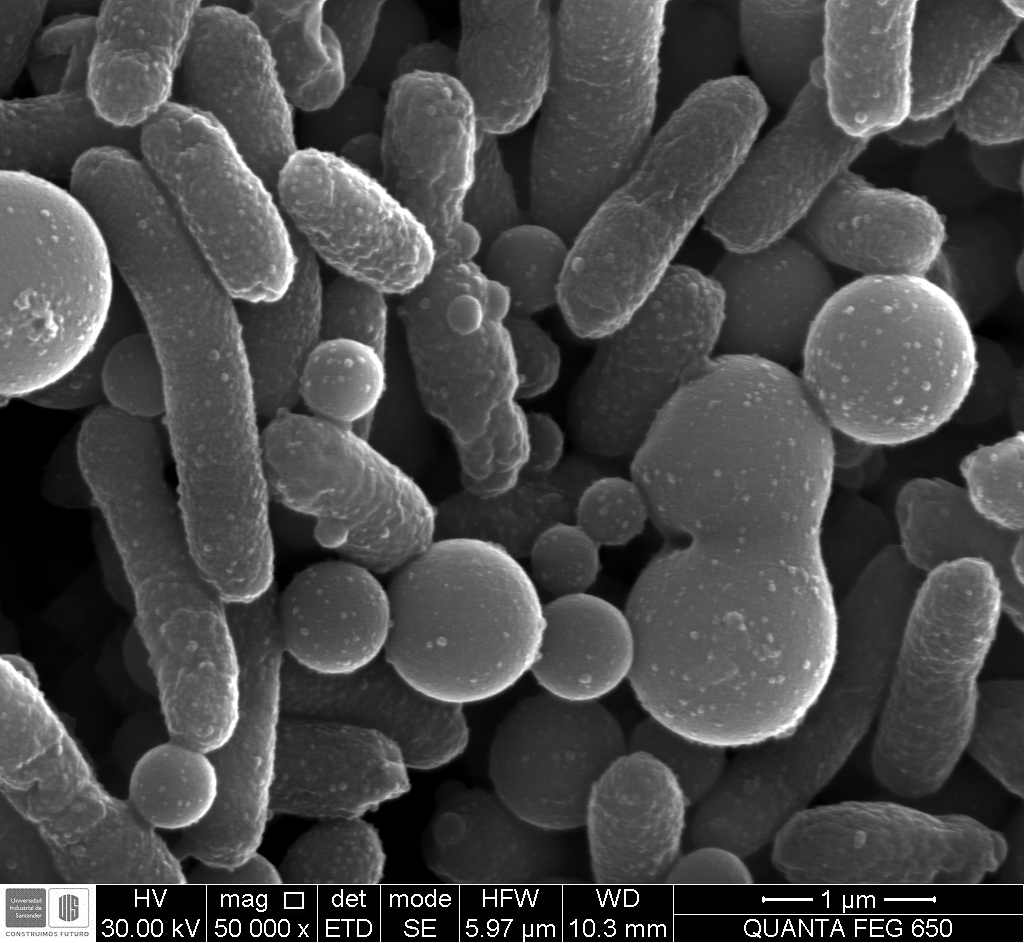

Supplement: S2 Raw Images — (ZIP) [file pone.0334029.s010.zip › S5Fig/S3E_50X.tif]

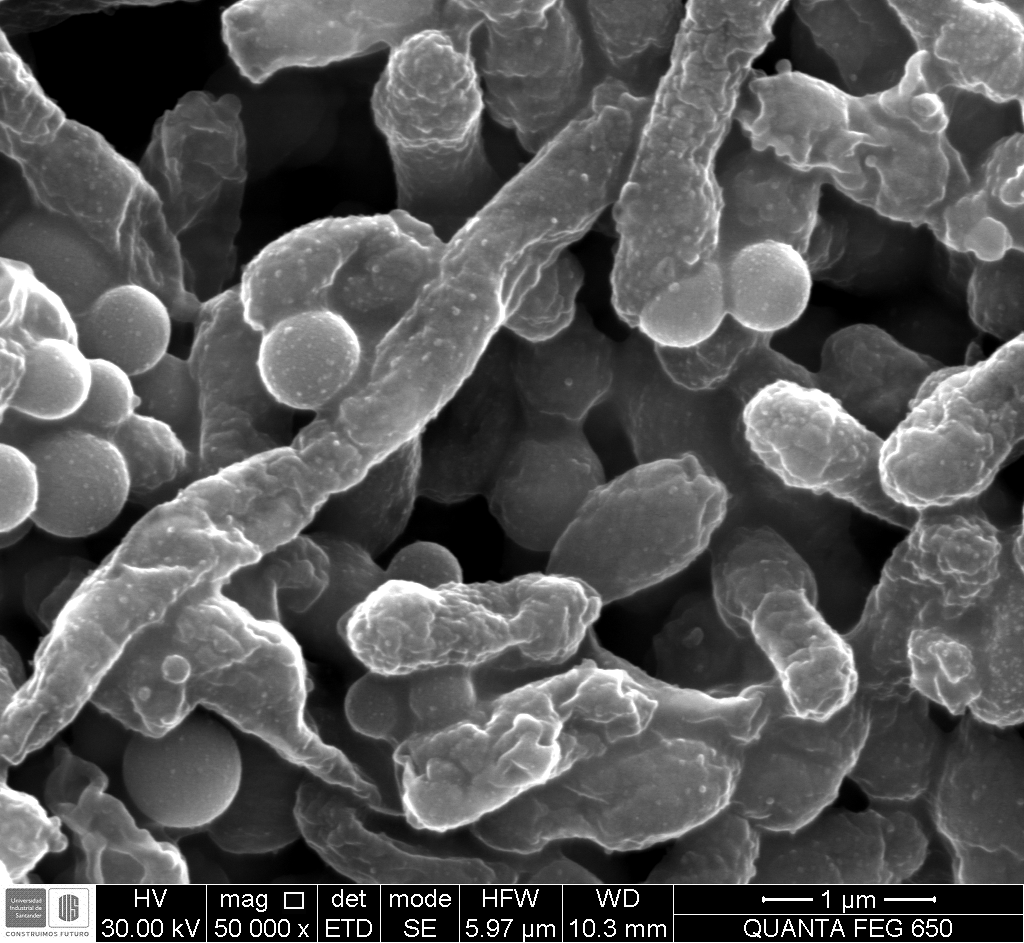

Supplement: S2 Raw Images — (ZIP) [file pone.0334029.s010.zip › S5Fig/S3F_50X.tif]

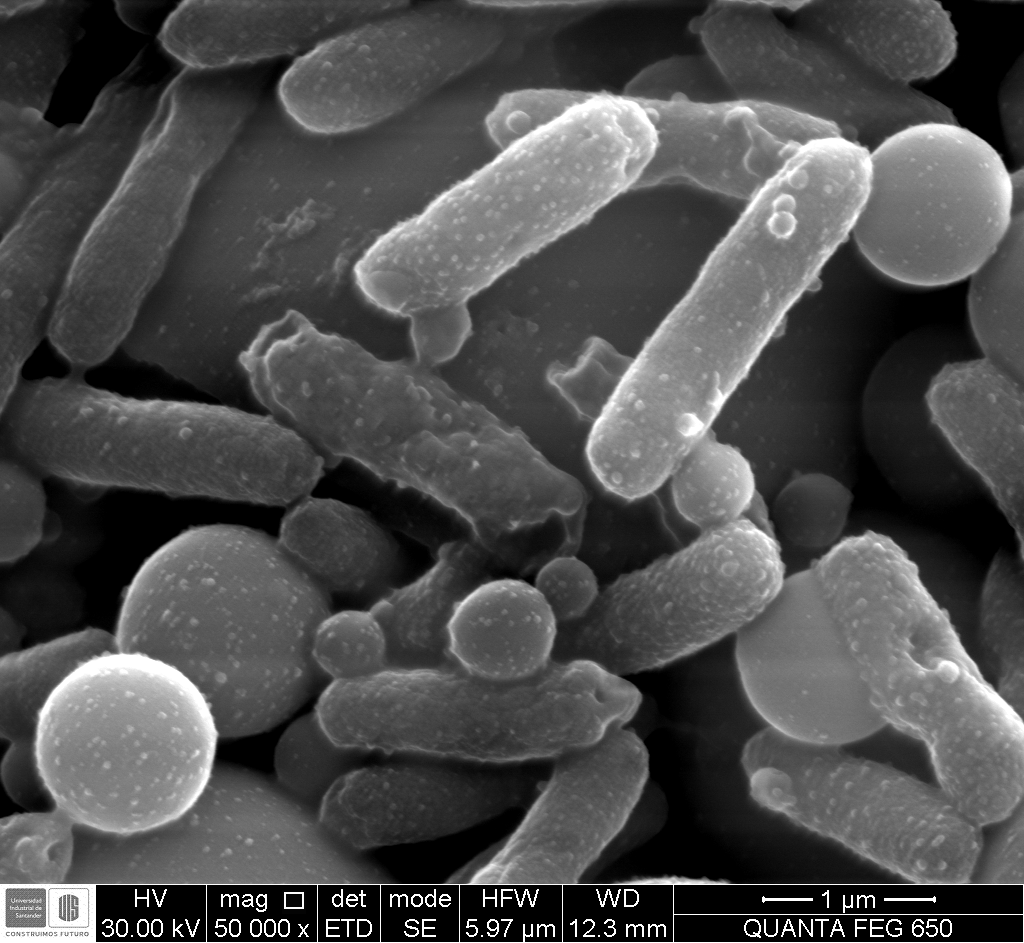

Supplement: S2 Raw Images — (ZIP) [file pone.0334029.s010.zip › S5Fig/S3G_50X.tif]

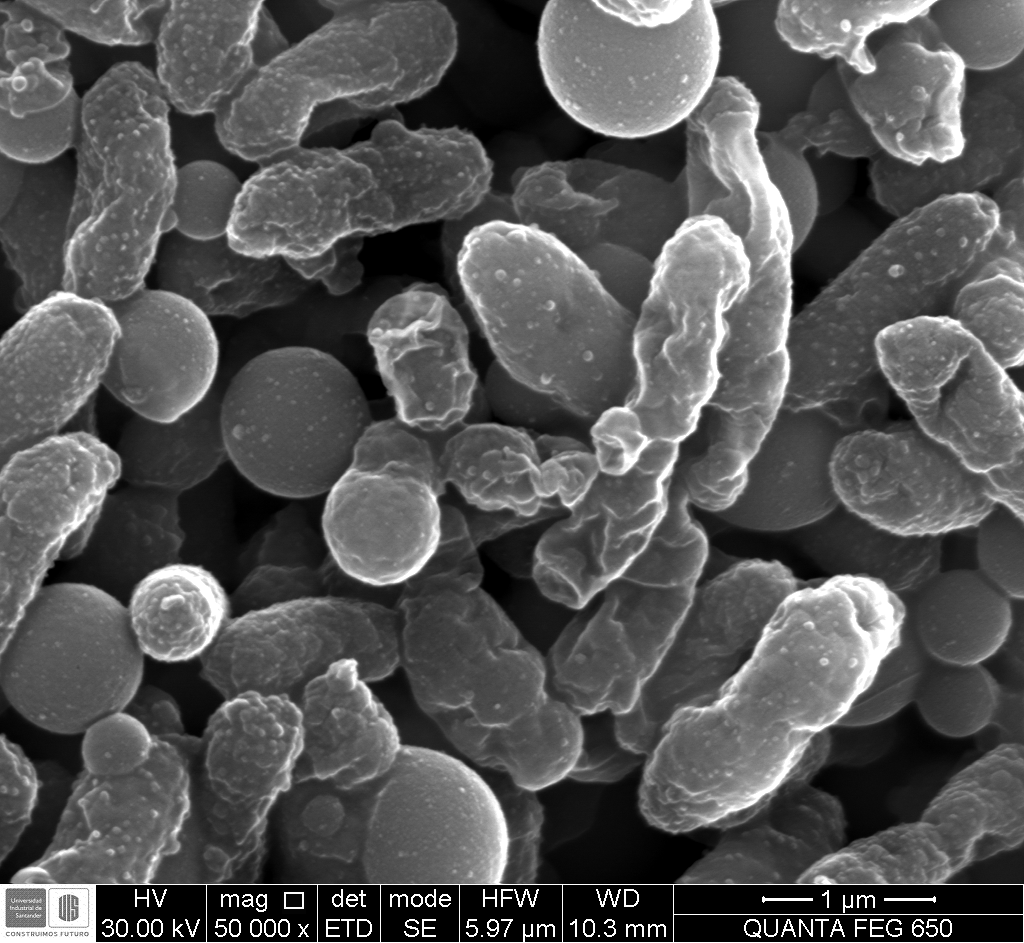

Supplement: S2 Raw Images — (ZIP) [file pone.0334029.s010.zip › S5Fig/S3H_50X.tif]

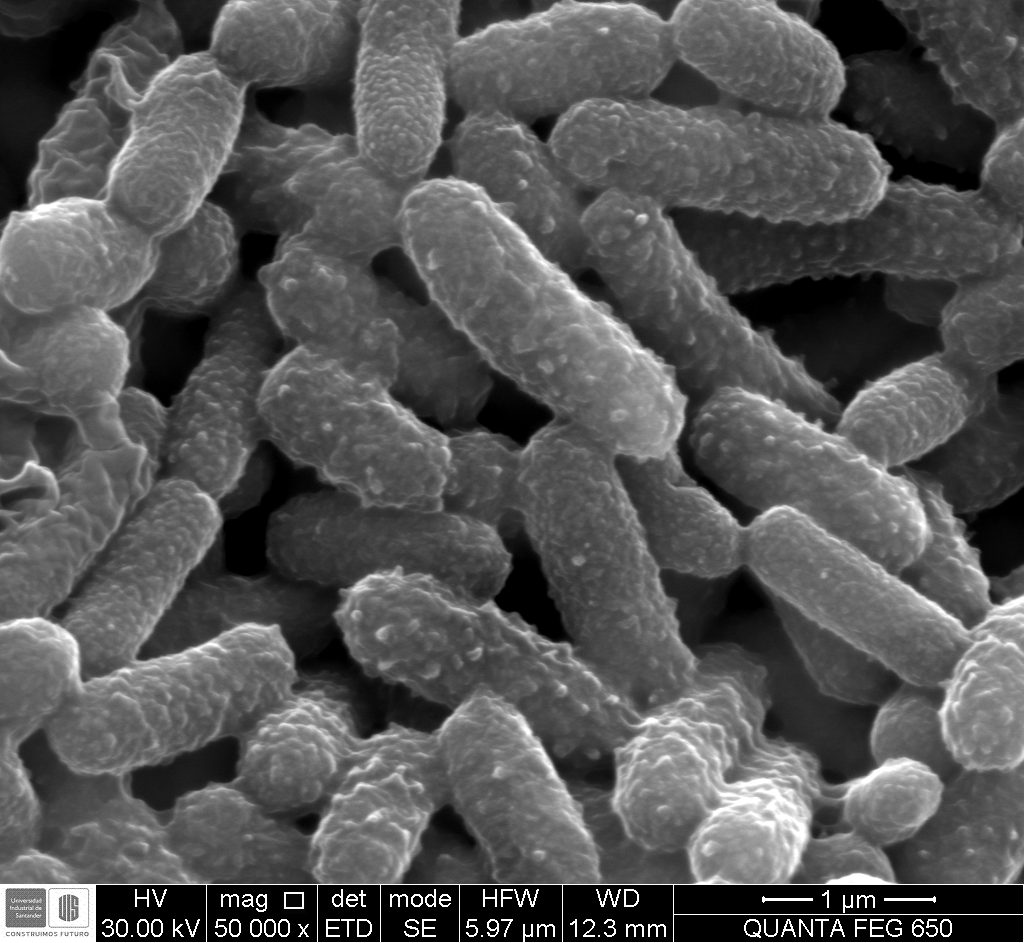

Supplement: S2 Raw Images — (ZIP) [file pone.0334029.s010.zip › S5Fig/S3I_50X.tif]

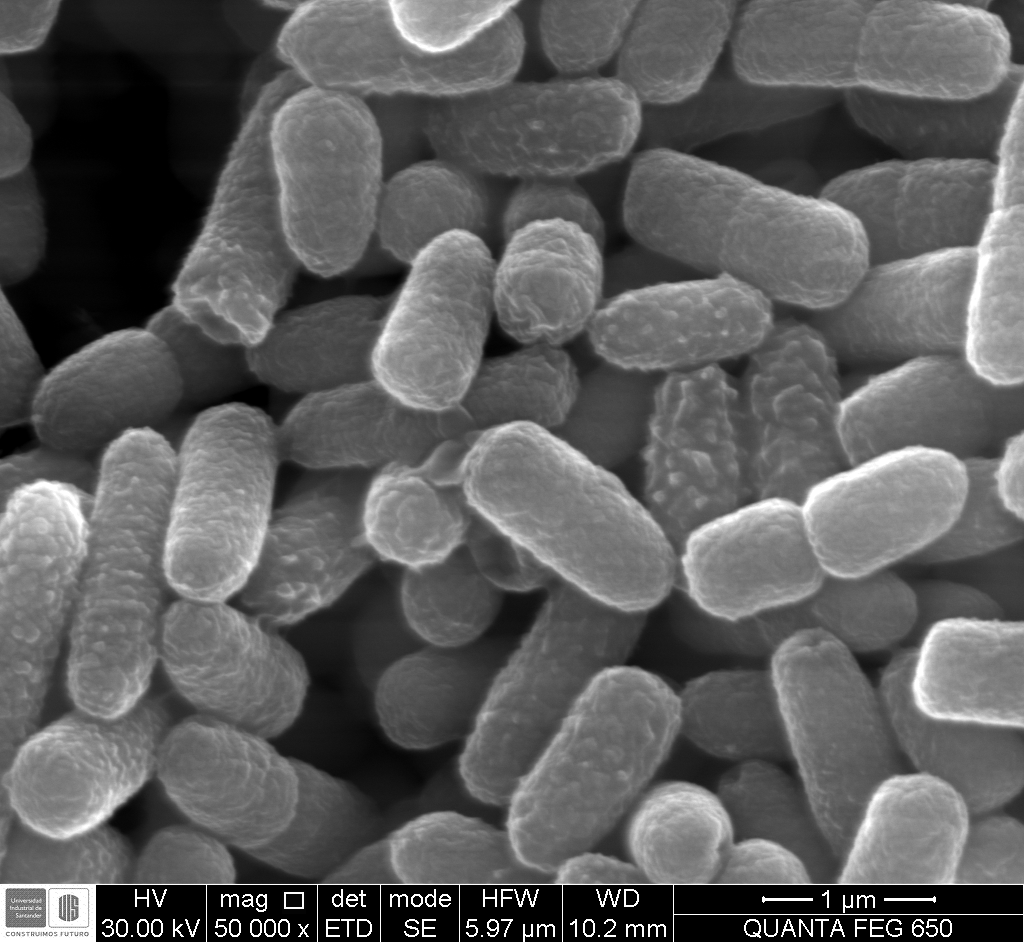

Supplement: S2 Raw Images — (ZIP) [file pone.0334029.s010.zip › S5Fig/S3J_50X.tif]

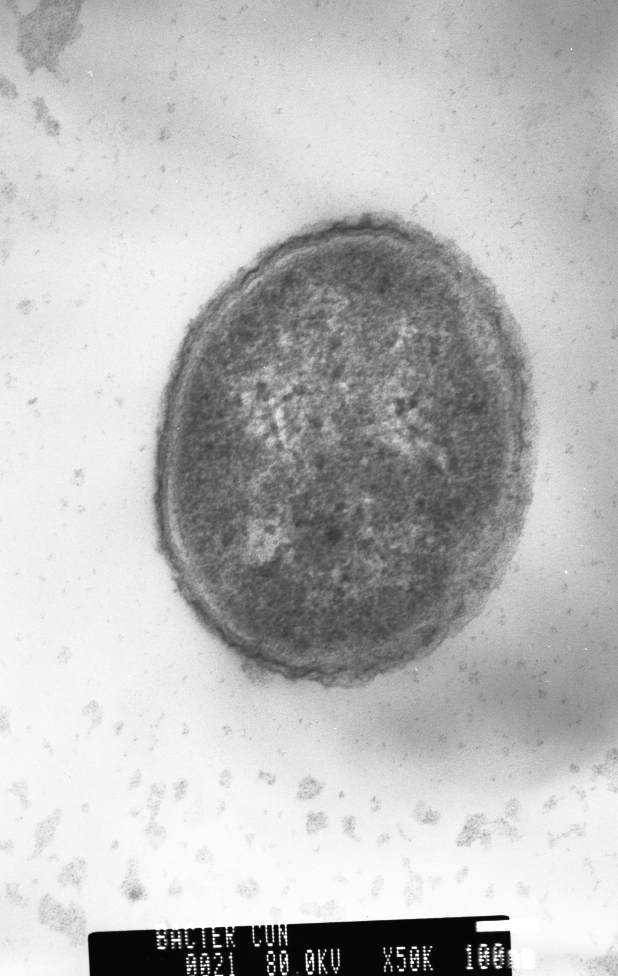

Supplement: S3 Raw Images — (ZIP) [file pone.0334029.s011.zip › S6Fig/3A_50X.tif]

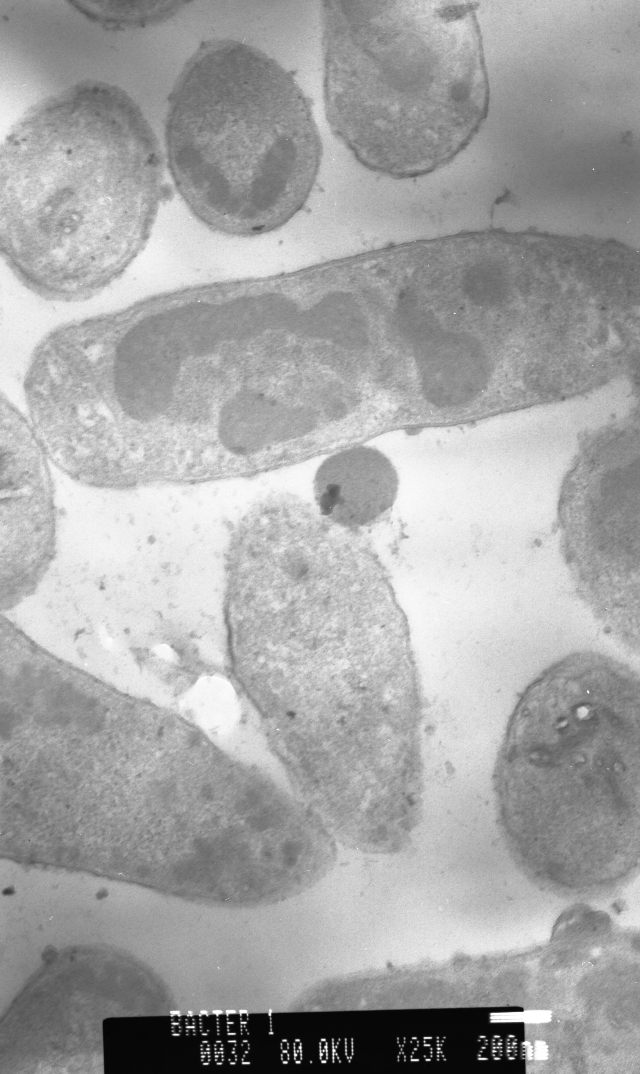

Supplement: S3 Raw Images — (ZIP) [file pone.0334029.s011.zip › S6Fig/3B_25X.tif]

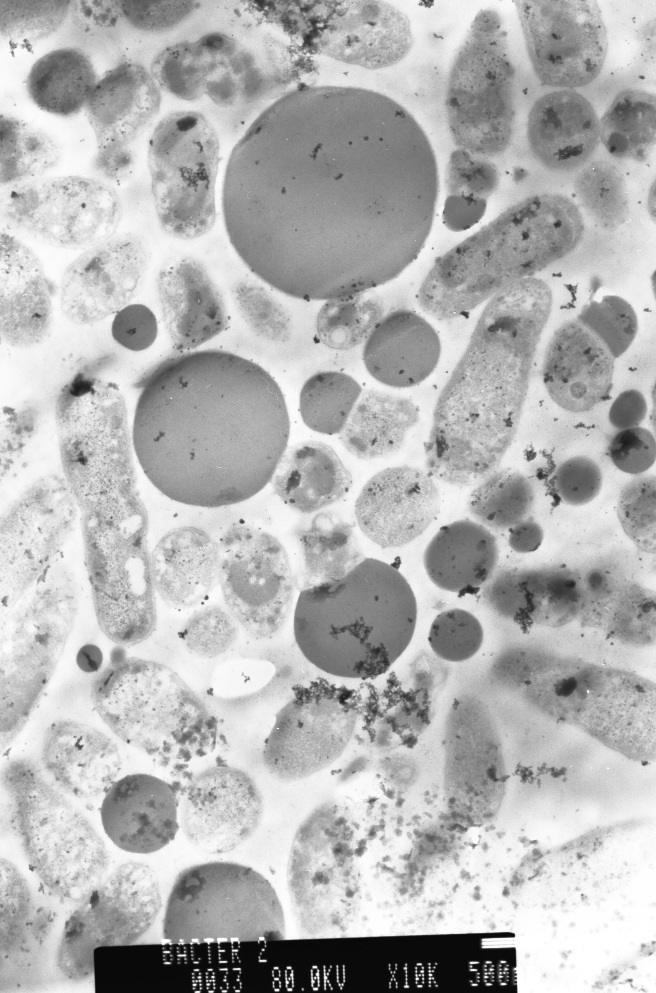

Supplement: S3 Raw Images — (ZIP) [file pone.0334029.s011.zip › S6Fig/3C_10X.tif]

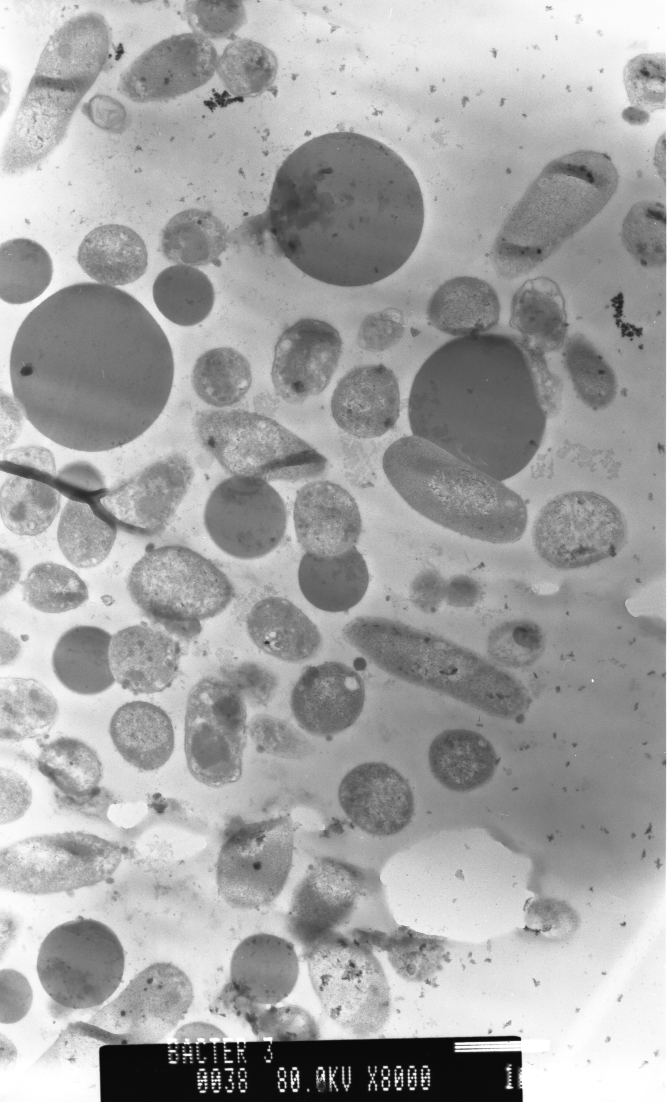

Supplement: S3 Raw Images — (ZIP) [file pone.0334029.s011.zip › S6Fig/3D_8X.tif]

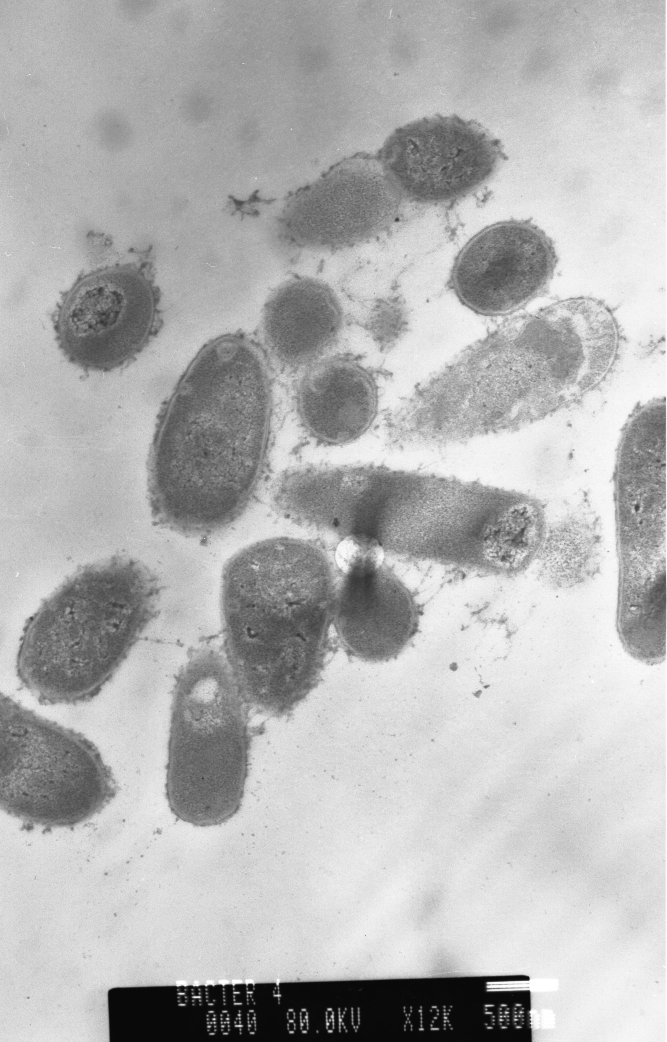

Supplement: S3 Raw Images — (ZIP) [file pone.0334029.s011.zip › S6Fig/3E_12X.tif]

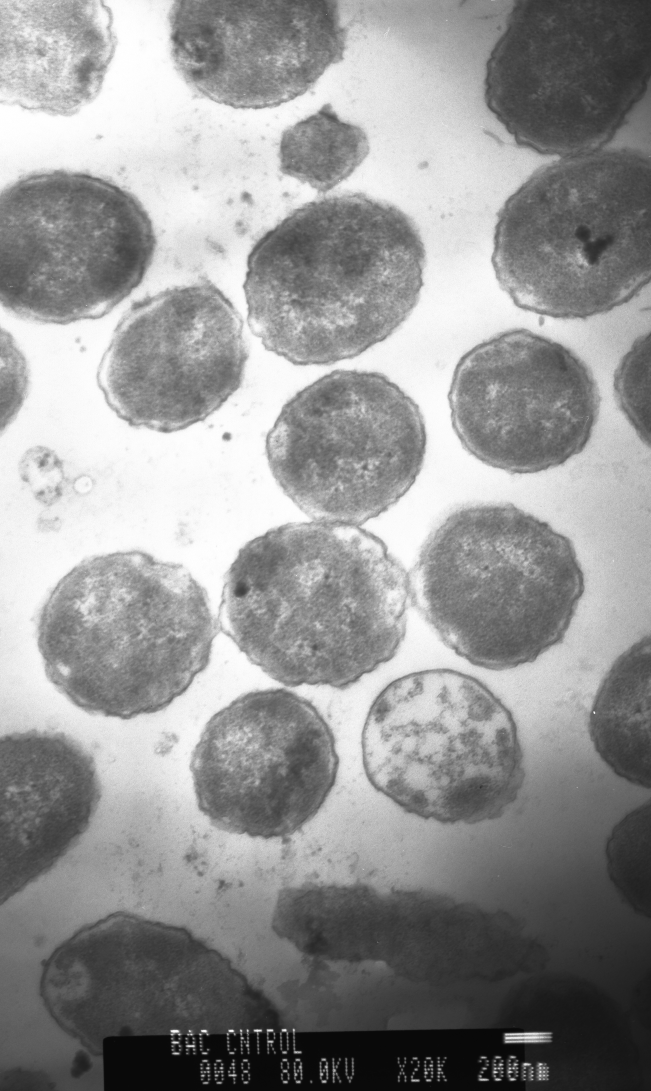

Supplement: S3 Raw Images — (ZIP) [file pone.0334029.s011.zip › S6Fig/3F_20X.tif]

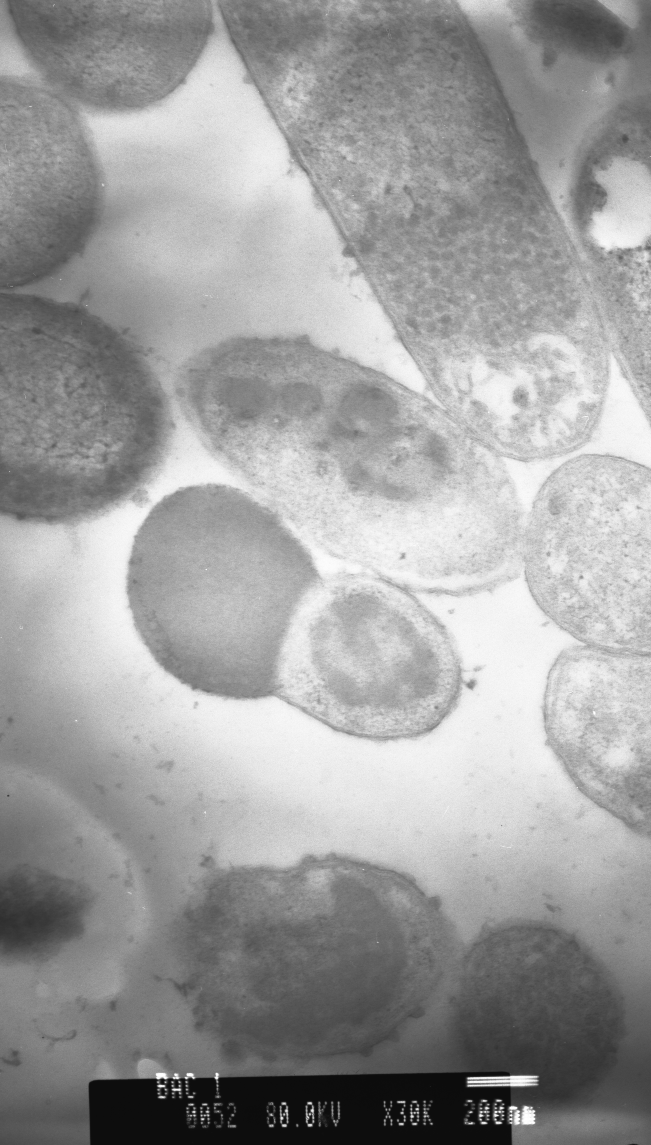

Supplement: S3 Raw Images — (ZIP) [file pone.0334029.s011.zip › S6Fig/3G_30X.tif]

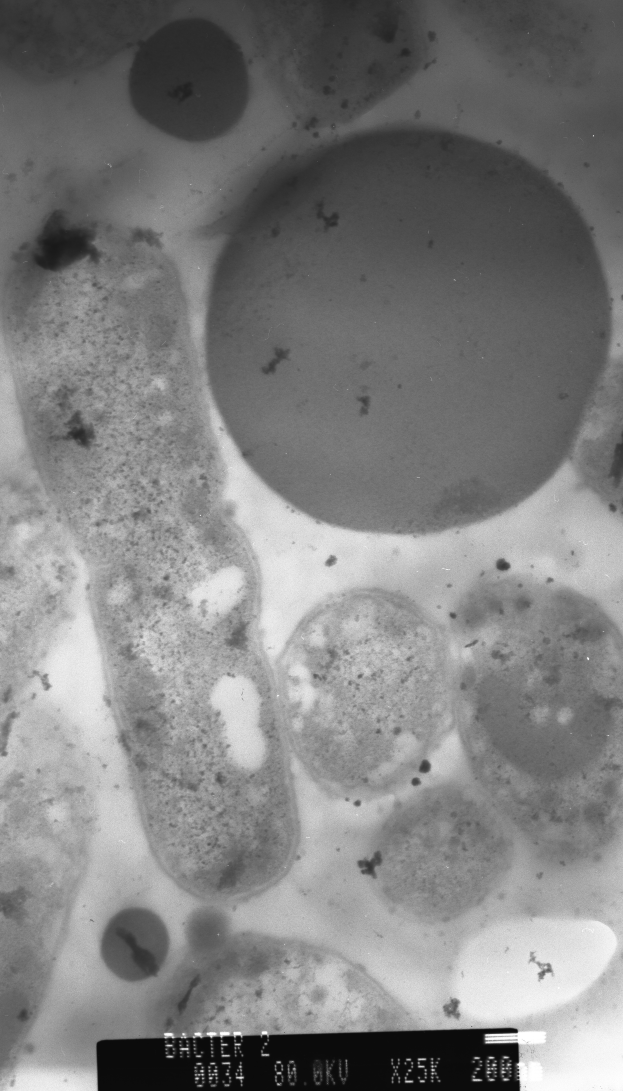

Supplement: S3 Raw Images — (ZIP) [file pone.0334029.s011.zip › S6Fig/3H_25X.tif]

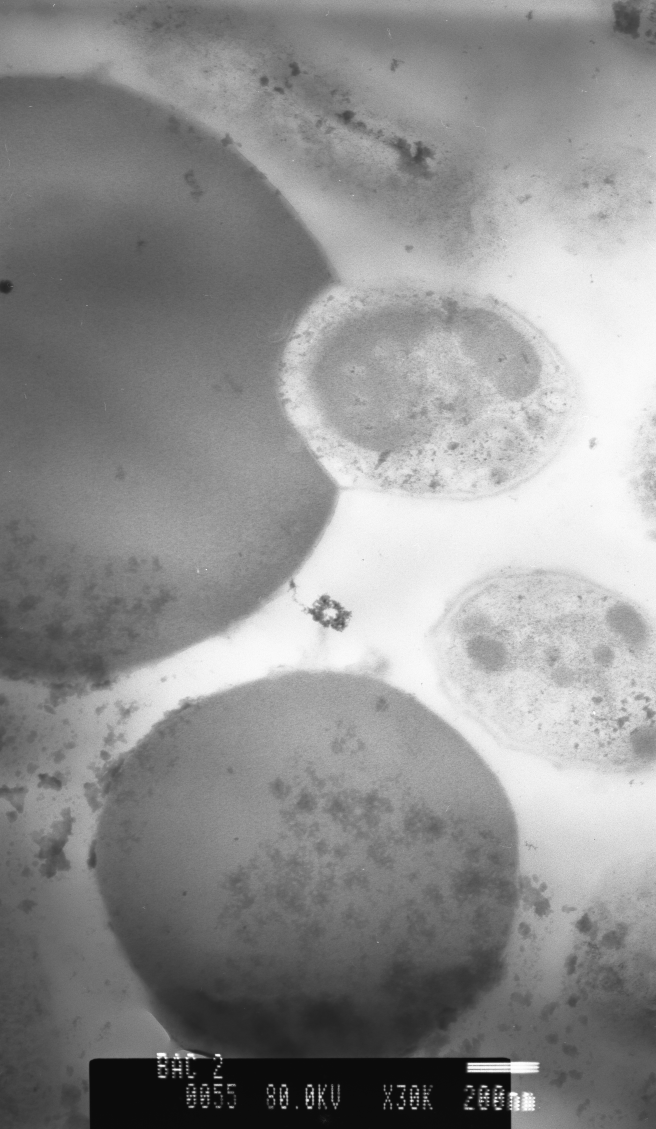

Supplement: S3 Raw Images — (ZIP) [file pone.0334029.s011.zip › S6Fig/3I_30X.tif]

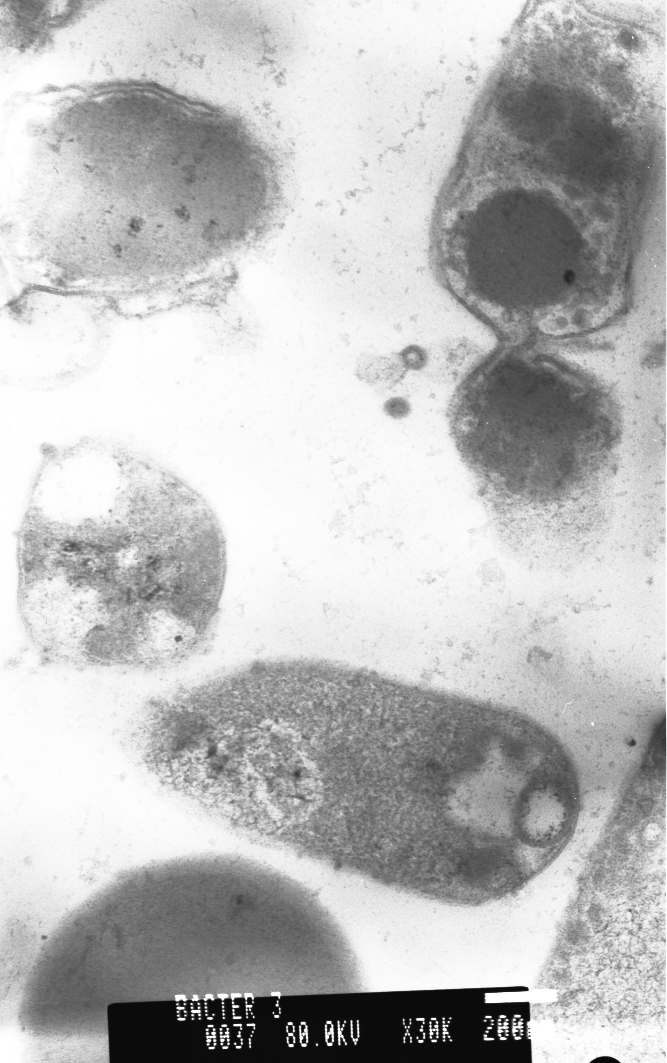

Supplement: S3 Raw Images — (ZIP) [file pone.0334029.s011.zip › S6Fig/3J_30X.tif]

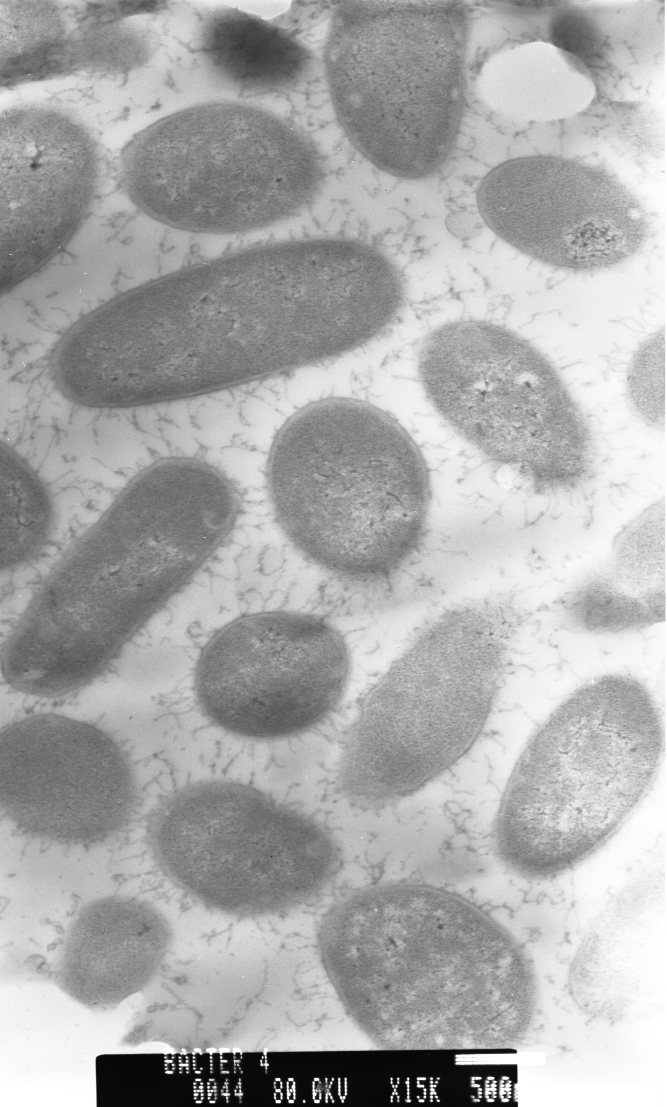

Supplement: S3 Raw Images — (ZIP) [file pone.0334029.s011.zip › S6Fig/3K_15X.tif]
